# Lipidomes of lung cancer and tumour-free lung tissues reveal distinct molecular signatures for cancer differentiation, age, inflammation, and pulmonary emphysema

**DOI:** 10.1038/s41598-017-11339-1

**Published:** 2017-09-11

**Authors:** Lars F. Eggers, Julia Müller, Chakravarthy Marella, Verena Scholz, Henrik Watz, Christian Kugler, Klaus F. Rabe, Torsten Goldmann, Dominik Schwudke

**Affiliations:** 1 Research Center Borstel, Bioanalytical Chemistry, Parkallee 1-40, 23845 Borstel, Germany; 2 Pathology of the University Hospital of Lübeck and the Research Center Borstel, Location Borstel, Clinical and Experimental Pathology, 23845 Borstel, Germany; 3 Pulmonary Research Institute at LungenClinic Großhansdorf, Wöhrendamm 80, 22927 Großhansdorf, Germany; 4 Airway Research Center North, German Center for Lung Research, Wöhrendamm 80, 22927 Großhansdorf, Germany; 5 LungenClinic Großhansdorf, Wöhrendamm 80, 22927 Großhansdorf, Germany

## Abstract

Little is known about the human lung lipidome, its variability in different physiological states, its alterations during carcinogenesis and the development of pulmonary emphysema. We investigated how health status might be mirrored in the lung lipidome. Tissues were sampled for both lipidomic and histological analysis. Using a screening approach, we characterised lipidomes of lung cancer tissues and corresponding tumour-free alveolar tissues. We quantified 311 lipids from 11 classes in 43 tissue samples from 26 patients. Tumour tissues exhibited elevated levels of triacylglycerols and cholesteryl esters, as well as a significantly lower abundance of phosphatidylglycerols, which are typical lung surfactant components. Adenocarcinomas and squamous cell carcinomas were distinguished with high specificity based on lipid panels. Lipidomes of tumour biopsy samples showed clear changes depending on their histology and, in particular, their proportion of active tumour cells and stroma. Partial least squares regression showed correlations between lipid profiles of tumour-free alveolar tissues and the degree of emphysema, inflammation status, and the age of patients. Unsaturated long-chain phosphatidylserines and phosphatidylinositols showed a positive correlation with a worsened emphysema status and ageing. This work provides a resource for the human lung lipidome and a systematic data analysis strategy to link clinical characteristics and histology.

## Introduction

The anatomical organisation of the human lung enables the exchange of gases between blood and air. Alveoli represent the basic units for gas exchange within the lung. Pulmonary surfactant, which lines the inner surface of the lung, prevents alveolar collapse at the end of expiration^1^. It comprises a complex mixture of mainly phospholipids and surfactant proteins. The main components are saturated phosphatidylcholine (PC) species such as PC 16:0/16:0, PC 16:0/14:0 and PC 16:0/16:1^2^. Phosphatidylglycerol (PG) is, with 7 to 15% of total phospholipids^3^, the second most abundant surfactant lipid class and plays a crucial role as immune regulator^3–5^.

The functional connection between the lipidome and tissues, as well as cell types, has been investigated by using biological model organisms such as *Drosophila melanogaster* ^6^ and *Mus musculus* ^7^. A recent study showed that widespread alterations of phospholipid profiles occurred in lung cancer tissues^8^. These alterations especially affect pulmonary surfactant components, but also sphingomyelin (SM)^8^. Further investigation by mass spectrometric imaging showed that lipidomes of cancer cells comprise specific alterations within surfactant lipids.

In this study, we analysed tissues from non-small cell lung cancer (NSCLC) and corresponding tumour-free alveolar control tissues by applying shotgun lipidomics screens^9, 10^. In addition to the abundant major phospholipid classes PC, phosphatidylethanolamine (PE), phosphatidylinositol (PI), phosphatidylserine (PS) and SM, we included the neutral lipids diacylglycerol (DAG), triacylglycerol (TAG) and cholesteryl ester (CE). We also included the sphingolipids ceramides (Cer) and hexosylceramides and the glycerophospholipids including PG and phosphatidic acid (PA). Clinical characteristics such as age, gender, body mass index (BMI), history of smoking, and quantitative scores for histological phenotypes were also assessed. Using this information, we systematically evaluated the multivariate statistical methods suitable for relating lipidomics data with individual clinical and histological characteristics. Omics data were explored by hierarchical clustering, principal component analysis (PCA) and partial least squares (PLS) regression^11–14^.

It is known that cells undergo a significant reprogramming of their metabolic networks during carcinogenesis, which is mirrored in systematic lipidome changes^8, 15^. Therefore, it is possible to evaluate several data interpretation strategies in terms of the physiological impact of a phenotype. From this perspective, we expected to find the most prominent alterations in the comparison between lipidomes of tumour and tumour-free control tissue, because of the profound metabolic reprogramming of cancer cells. On the other hand, we expected the influence of ageing on the lipidomes of alveolar tissues to be much more elusive. Hence, we followed this logic to test the application range of certain data analysis strategies. These range from objectively large histopathological changes that affect the composition of the lipidome itself, to subtly nuanced alterations in clinical parameters such as age, BMI, and gender. This study enabled us to compile a resource for the human lung lipidome that shows how histological diversity and clinical data are reflected in distinct molecular features.

## Results

### Customised screening improves lipidome coverage

To gain insight into the general lipidome composition of lung tissues, we used a customised lipidomics screening approach^9, 10, 16, 17^. We devised sample preparation and extraction procedures that had small sample size requirements and could be performed in a typical laboratory. Mass spectrometric conditions for negative ionisation were optimised to improve sensitivity for negatively charged lipids such as PI, PG, PA and PS (Supplement 1, Supplementary Fig. S1). Using ammonium chloride as an additive for electrospray ionisation, we circumvented the overlapping of mass spectrometric signals of PC with PS (Supplementary Fig. S2). In this way, unambiguous quantification of lipids from both classes was achieved. We determined the optimal ammonium chloride concentration to be 0.05 mM, which yielded the highest responses for negatively charged lipids (Supplementary Fig. S2, Supplement 2). We estimated a dynamic range of 3 orders of magnitude for the lipid quantitation of our mass spectrometric system. To this end, 311 lipids from 11 classes were quantified: CE, Cer, cardiolipins, DAG, PC, PE, PG, PI, PS, SM, and TAG (Supplement 3).

### Lipidomes of tumour-free alveolar tissues and tumours show distinct molecular compositions

**Figure 1:**
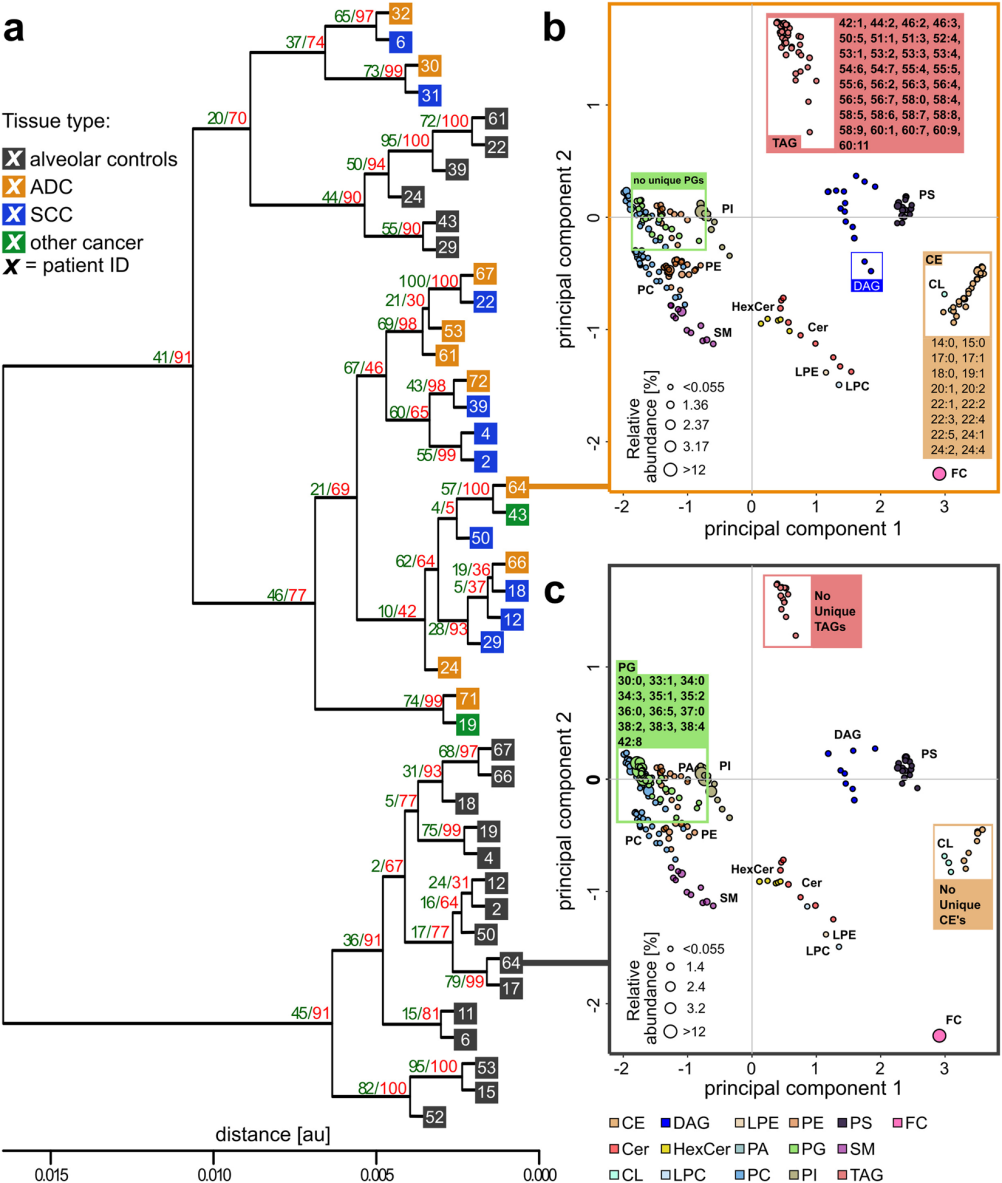
Tumour and alveolar control tissue lipidomes have distinct compositions. (**a**) Hierarchical clustered tree of 43 tissue lipidomes based on the LUX score (Supplement 4). The frequency of reoccurrence of each branch after error modelling using 100 iterations is indicated with green numerals for t  = 0.005, SD = 0.002 and red numerals for t = 0.003, SD = 0.001. (**b**) Lipidome map of patient ID64 tumour tissue. (**c**) Lipidome map of the matched alveolar control tissue of patient ID64. Each point on the lipidome map represents a lipid, and distances between lipids represent their structural similarity. The size of the data point corresponds to the quantity. Uniquely identified lipids in either tumour or alveolar tissues belonging to TAG, CE and PG classes are highlighted. ADC: adenocarcinoma; SCC: squamous cell carcinoma; FC: free cholesterol.

Several characteristic lipid species were only present in either alveolar control tissues or tumour tissues. For example, in the tumour lipidome of patient ID64, we quantified 33 TAG and 16 CE species that were not detected in the matched alveolar control tissue (Fig. 1b,c). We also identified 13 PG species that were exclusively detected in the alveolar tissue lipidome. These remarkable changes were observed in a number of paired tissue samples, indicating that widespread compositional changes occurred in tumour lipidomes. For a systematic characterisation of these changes, we applied an earlier reported method to determine lipidome homologies based on the LUX (Lipidome Juxtaposition) score^18^ (Supplement 4). The hierarchical clustered tree based on the LUX score showed that tumour and alveolar tissue lipidomes have distinct compositional features (Fig. 1a). We observed a robust tree morphology consisting of two main branches. The first branch exclusively comprised alveolar control tissues, and the second was populated by all tumours plus a cluster of six alveolar tissues. Significantly, tumour and control samples from the same patient were never clustered together. This finding supports the notion that a patient-independent tumour lipidome comprises structural entities that are not present in alveolar controls. When PCA was applied to LUX scores, a clear separation of tumour tissues and alveolar control tissues along principal component 1 (PC1) was observed (Supplementary Fig. S3). The distribution of individual lipidomes along PC1 was used for tissue classification and showed high sensitivity and specificity in a receiver operating characteristics (ROC) curve, with an area under the curve (AUC) of 0.983 and p < 0.0001 (Supplementary Fig. S3). Our data showed that the large histopathological differences between alveolar control tissues and tumour tissues caused distinct compositional perturbations in lipidomes. However, we did not observe a difference between main tumour subtypes of NSCLC—adenocarcinomas (ADC) and squamous cell carcinomas (SCC)—by analysing LUX scores. No other clinical characteristics were linked by using the unsupervised LUX score methodology.

### Lipidome alterations of NSCLC tissues revealed by correlation analyses

**Figure 2:**
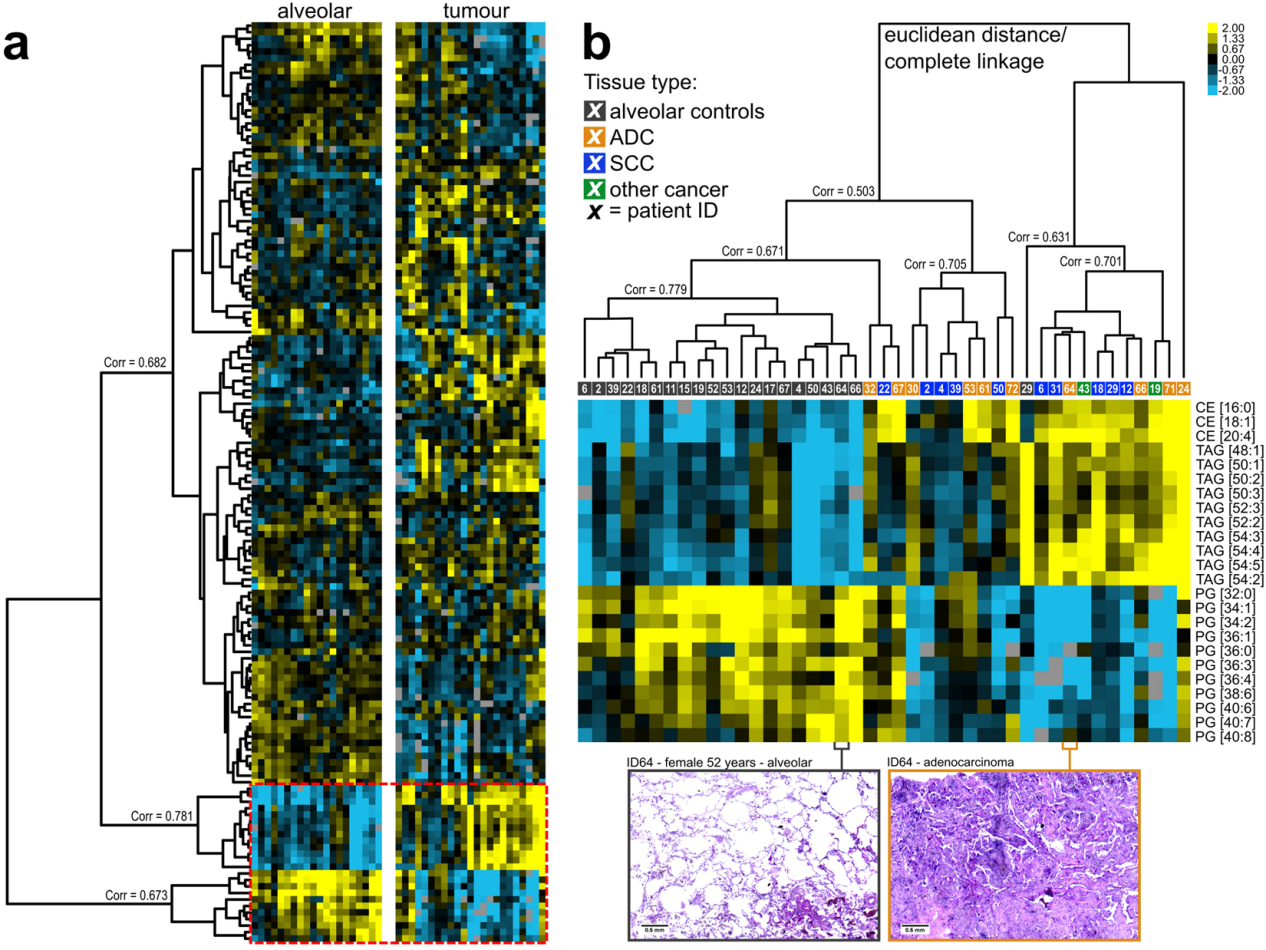
Tumour and alveolar control tissues can be distinguished by cluster analysis of lipid profiles. (**a**) Heat-map of 141 lipid quantities identified in 43 lung tissues (Supplement 11). The corresponding hierarchical clustered tree is shown in panel b. (**b**) Individual lipidomes are colour coded according to the tissue type. Scans of histology slices of tumour and alveolar control tissue of patient ID64 are shown at the bottom.

To gain further insight into lung lipidome characteristics, we applied unsupervised hierarchical cluster analysis using lipid quantities as input data. For this analysis, 141 lipids that were present in at least 90% of all samples were considered (Supplement 3). Hierarchical clustering revealed a clear-cut separation between tumour and alveolar control tissues (Fig. 2). All alveolar control tissues clustered together with a correlation coefficient of 0.779 (Fig. 2b, left cluster), with the exception of ID29, a patient suffering from Crohn’s disease. In general, neutral lipids such as CE and TAG were more abundant in tumour tissues (Fig. 2b). This trend was observed in all samples, with a correlation coefficient of 0.781 (Fig. 2a). Furthermore, DAG [34:1], DAG [34:2], DAG [36:3] and DAG [36:1] showed the same trend (Supplementary Fig. S4). The opposite trend was observed for surfactant main components, most prominently for PG lipids. PG lipids were less abundant in tumours (Fig. 2b), and samples showed high correlation, clustering together with a correlation coefficient of 0.673 (Fig. 2a). The surfactant main components PC [32:0], PC [30:0] and PC [28:0] were also less abundant in tumours (Supplementary Fig. S4), which further supported the hypothesis that a loss of surfactant components is a general feature of tumorigenesis. We further observed that a number of lipids, mostly long-chain SM, PG and PS, increased in abundance in alveolar control tissues (Supplementary Fig. S4). We did not observe a difference between the main NSCLC types ADC and SCC in these analyses. Interestingly, the ADC ID24 was clearly different from all other cancer tissues (Fig. 2b), most likely owing to the high proportion of necrosis. The clear differentiation between tumour and alveolar tissues was confirmed with a complementary PCA of the same input data (Supplementary Fig. S5). Furthermore, distinguishable clusters for ADC and SCC were observed for the PC1/PC3 factor map (Supplementary Fig. S5).

Both hierarchical clustering and PCA indicated that there is greater heterogeneity among lipidomes of tumour tissues than among those of alveolar control tissues. Using hierarchical clustering, we observed a complex branching structure of the tumour lipidomes, which resulted in a number of subclusters that showed low correlation with each other (Fig. 2b). Using PCA, we observed that the heterogeneity was apparently greater for tumour tissues than for alveolar control tissues, as deduced from the computed confidence areas (Supplementary Fig. S5).

### Tissue classification based on lipid panels

**Figure 3:**
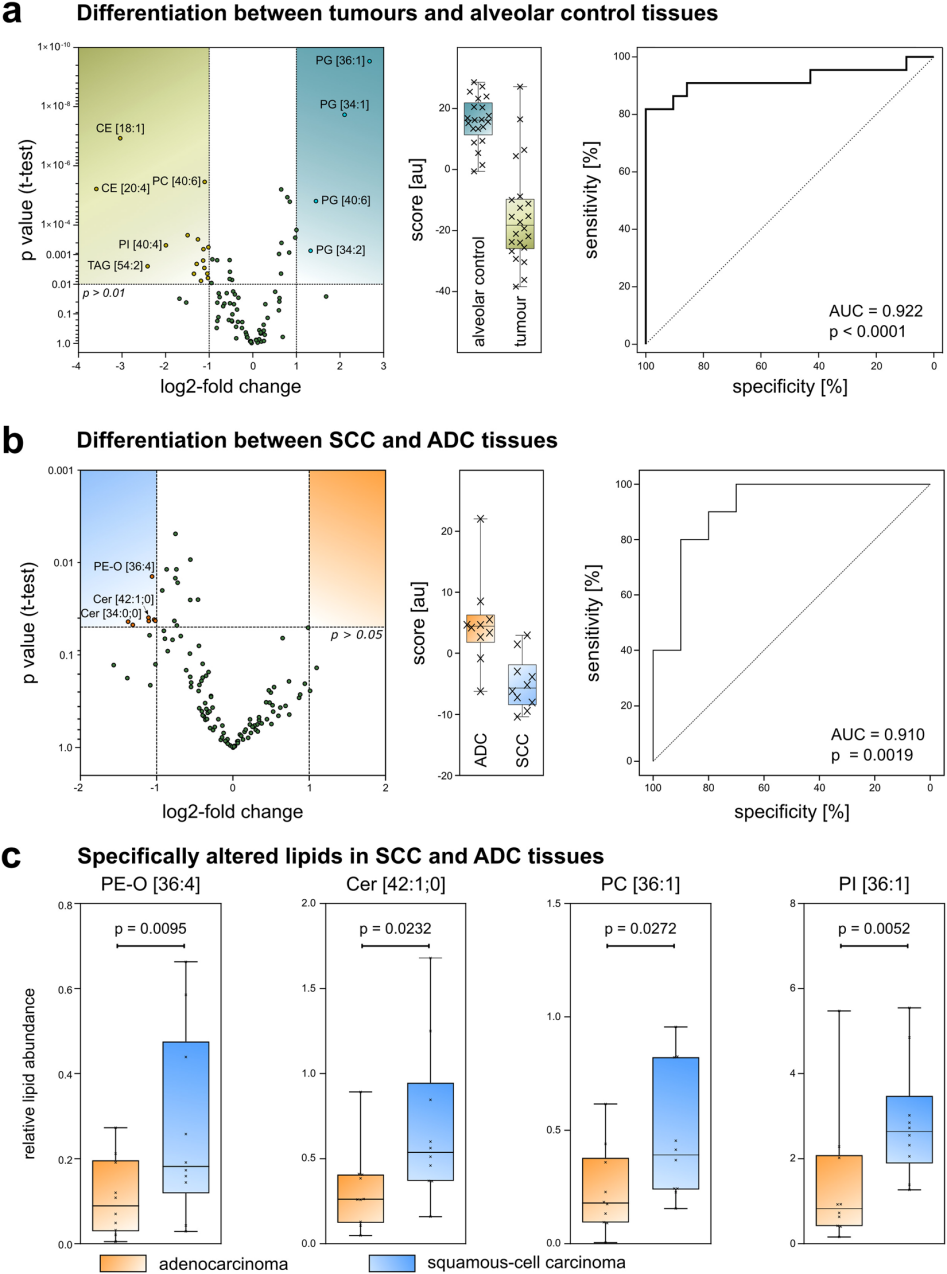
Tumour tissue types can be differentiated by a lipid panel. (**a**) Identification of tumour tissue based on its lipidome. Volcano plot showing significantly changing lipid quantities in tumour tissues and alveolar control tissues. Boxplot of the resulting score and ROC curve for the differentiation between tumour tissues and alveolar control tissues (au: arbitrary unit). (**b**) Volcano plot, boxplot and ROC curve to distinguish ADC and SCC tissue samples. (**c**) Boxplots of selected lipid quantities differing between ADC (n = 10) and SCC (n = 10). The p value was calculated using the Mann-Whitney U test.

Next, we asked if the observations made by cluster analysis and PCA could be confirmed by a scoring system to classify tissues. For this investigation, 92 lipids that were detected in every sample were evaluated for their discriminative power (Fig. 3a). Twenty lipids that were significantly changed, with a p value < 0.01 and at least a twofold change between the groups (mostly PGs, CEs and TAGs), were chosen as a panel to discriminate between tumour tissues and alveolar control tissues (Supplement 3). The resulting score, calculated from the abundance of these lipids, classifies a sample as tumour tissue if it has a value of about −20 au (boxplot, Fig. 3a). A score of about +20 au would identify a sample as alveolar tissue. The ROC curve indicated that the score works well to differentiate between tumour and alveolar control tissues (AUC = 0.922; p < 0.0001). However, the ROC curve based on LUX scores performed even better, with an AUC = 0.983 (Supplementary Fig. S3). We concluded that lipidome data are sufficient to distinguish tumour tissue from alveolar tissue through the extensive changes in the lipidome that occur during tumorigenesis, as reported previously^8, 19^.

We then investigated the possibility of distinguishing between ADC and SCC tissues. In a similar manner, 113 lipids of the 20 ADC and SCC samples were screened for their discriminative power (Fig. 3b). Seven lipids with significantly increased quantities (p < 0.05) in SCC tissues compared to those in ADC tissues were selected for the score calculation (Fig. 3b and Supplement 3). The resulting score indicates ADC if a value of ~5 au is computed, and SCC for a value of about −5 au. The ROC curve indicates good sensitivity and specificity with AUC = 0.910 and p = 0.0019 (Fig. 3b). As examples for the lipid panel components, the abundance of PE-*O* [36:4], Cer [42:1;0], PC [36:1] and PI [36:1] is shown in Fig. 3c. Individual marker lipids alone did not achieve the high specificity of the panel (Supplementary Table S6).

Numerous factors potentially affect the composition of the human lung lipidome. From this perspective, we clearly reached the limit of unsupervised multivariate approaches to associate functional and phenotypical data with the lung lipidome. Besides the large perturbations in lipidomes caused by carcinogenesis, no other phenotype was associated by using hierarchical clustering and PCA. Our next step was to determine if clinical characteristics (Table 1) influenced the lipidome composition using PLS regression. Because of the notable differences between tumour and alveolar control tissues, PLS regression analyses were performed separately for tumours and alveolar tissues. This also allowed us to study the effect of tissue-specific histopathological phenotypes on the lipidome composition.

**Table 1:** Clinical characteristics of the analysed cohort (26 patients).

Patient ID	Gender	Tumour-free tissue*	Tumour tissue*	Cancer diagnosis	Age (years)	BMI	PY	GOLD stage
ID2	Male	+	+	SCC	71	35	60–70	1
ID4	Male	+	+	SCC	66	25	>100	1
ID6	Male	+	+	SCC	70	28	50	1
ID11	Male	+	−	ADC	48	NA	NA	NA
ID12	Male	+	+	SCC	70	21	50	2
ID15	Male	+	−	SCC	45	40	70	2
ID17	Male	+	−	SCC	68	NA	NA	2
ID18	Female	+	+	SCC	47	32	50	No COPD^#^
ID19	Female	+	+	other	54	28	35	1
ID22	Male	+	+	SCC	57	25	25	1
ID24	Male	+	+	SDC	55	26	40	No COPD^#^
ID29	Male	+	+	SCC	59	29	40	No COPD^#^
ID30	Male	−	+	ADC	52	27	30	3
ID31	Male	−	+	SCC	68	33	100–150	2
ID32	Male	−	+	ADC	45	23	30	1
ID39	Male	+	+	SCC	60	25	40	2
ID43	Male	+	+	other	60	23	30	1
ID50	Male	+	+	SCC	63	25	47	1
ID52	Female	+	−	other	44	27	10	Restriction with pneumonia
ID53	Male	+	+	ADC	48	29	30	No COPD^#^
ID61	Male	+	+	ADC	70	18	60	2
ID64	Female	+	+	ADC	51	27	35	1
ID66	Male	+	+	ADC	62	28	45	1
ID67	Female	+	+	ADC	52	30	50	2
ID71	Female	−	+	ADC	57	21	30	1
ID72	Male	−	+	ADC	62	34	40	2

NA, not available; BMI, body mass index; PY, pack-years; GOLD, Global Initiative for Chronic Obstructive Lung Disease; COPD, chronic obstructive pulmonary disease; ADC, adenocarcinoma; SCC, squamous cell carcinoma; other, tumour diagnosis was either large-cell carcinoma (ID19), sarcomatoid large cell carcinoma (ID43) and carcinoid tumour (ID52).

^*^Tissue sample availability for the analysis is indicated; for all lipidomics analysis, 14.4 mg wet-weight was extracted.

^#^For the diagnosis ‘No COPD’, the GOLD stage was set to zero for all data analysis strategies.

### The histopathology of NSCLC is reflected in the lipidomes

We performed PLS regression analysis on tumour tissue lipidomes to find the main factors that influence lipidome organisation. In this analysis, we included 113 lipids that were present in all tumour samples, as well as all clinical and histopathological data (Table 1 and Supplement 3).

**Figure 4:**
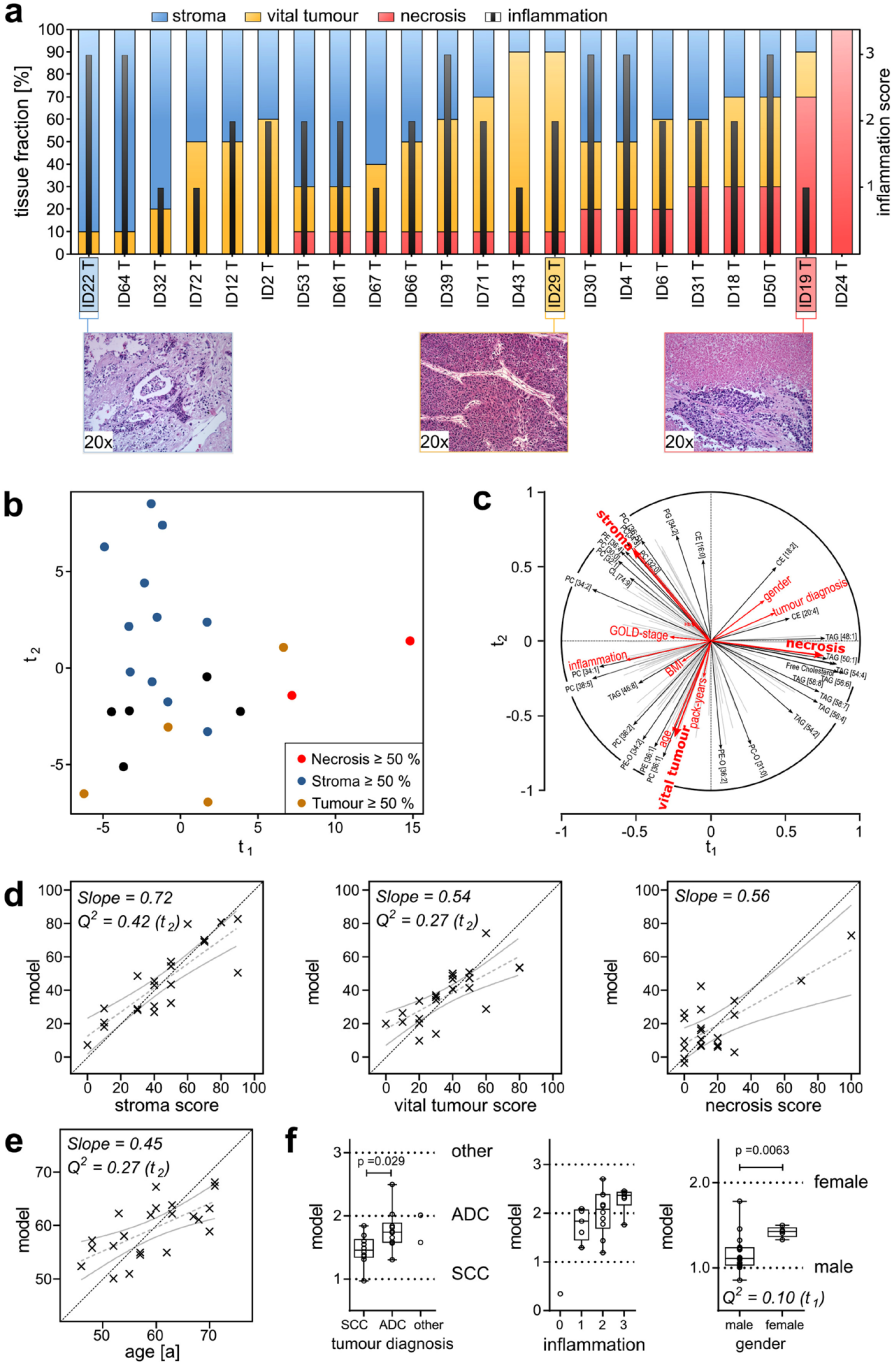
Factors influencing lung tumour lipidomes analysed by PLS regression. (**a**) Results of histological scoring. Sections were histologically characterised according to their tissue composition, using percentages of necrotic areas, vital tumour cells and stroma content. The infiltration by immune cells was scored in categories (0, 1, 2, 3). (**b**) Factor map of individual tumour lipidomes, colour coded according to the dominant tissue fraction (stroma ≥50%, blue; vital tumour cells ≥50%, yellow; necrotic areas ≥50%, red; none of the tissue fractions ≥50%, black). (**c**) Correlation of lipid quantities, histology scores, and clinical data to the t-components. The vectors indicate how strong the variables correlate to the t-components and show the correlations between the lipidome data (black arrows) and the histology scores and clinical data (red arrows). (**d**) Correlation of original histology scores to scores computed from the PLS regression model for stroma, vital tumour and necrosis. Linear regressions with 95% confidence bands are shown in grey. The specific slope is noted on the plot. The dotted black line indicates the location of the ideal correlation. (**e**) Correlation for the clinical parameter age. (**f**) Evaluation of the PLS regression model for tumour diagnosis, inflammation score, and gender. Boxplots show computed scores versus the original categorical values. Q^2^ values are the results of the cross-validation of the model. A model is considered significant for a response if Q^2^ ≥ 0.0975. The associated t-component is noted in brackets after the Q^2^ value. All p values were calculated using the Mann-Whitney U test. Q^2^ values and p values are noted only when significance was reached. See also Supplementary Fig. S7.

Tissue composition was assessed by determining the percentage of vital tumour, stroma and necrosis; inflammation status was also characterised (Fig. 4a). The histology scores of tumour samples indicated great heterogeneity in tissue composition. This indicates that extensive histological changes occur, which is also indicated by the examples showing 90% stroma (ID22), 80% vital tumour (ID29) and 70% necrosis (ID19). We expected such substantial morphological changes to be reflected in the lipidome structure. Indeed, PLS regression analysis revealed that tissue composition is the most influential factor shaping the lipidome of tumour biopsy samples (Fig. 4b,c and Supplement 5). Samples with high stroma content were separated from samples with high vital tumour content along the component t_2_, positioning high stroma samples in the top left corner of the factor map and high vital tumour samples at the bottom (Fig. 4b). We also noted that samples with high necrosis content were located at high t_1_ values. Stroma, vital tumour and necrosis content had a very distinct influence on the lipidome, characterised by the directional divergence of their vectors (Fig. 4c). Accordingly, a set of lipids could be specified that showed the best positive correlation to one of the tissue fractions; however, this would make marginal contributions to the others. Stroma content, PC [36:5], PC [34:3], and saturated surfactant-related lipids PC [32:0] and PC [30:0] showed the strongest correlation. Necrosis was mostly associated with TAGs and free cholesterol, whereas vital tumour content correlated with PC [36:1] and PE [36:1]. The predictive power of the PLS model was then evaluated using cross-validation. We found strong correlations for stroma and vital tumour, with Q^2^ values of 0.42 for stroma and 0.27 for vital tumour (Fig. 4d). For necrosis, cross-validation was not significant, most likely because only two samples had a content >50%. We further found a valid cross-validation for the age of patients with Q^2^ = 0.27 (Fig. 4e). However, at this point we could not exclude a dependence between age and vital tumour content, even though on the t_1_/t_3_ plane a nearly orthogonal behaviour was observed (Supplementary Fig. S7). For the categorical phenotypes, we compared the values computed by the model with those of the original categories and tested if computed values would reflect the original phenotype using the Mann-Whitney-U test. In this case, the PLS regression model had sufficient predictive power to distinguish ADC from SCC tissues (p = 0.029) and to distinguish gender (p = 0.0063) (Fig. 4f). However, tumour diagnosis and gender are most likely dependent parameters because of a bias for ADC for female patients (3 ADC, 1 SCC, 1 other) in the current data set. For inflammation stage 1 and stage 3, the computed values were significantly different (p = 0.03). Other parameters recorded in this study—e.g., haemoglobin content, pack-years of smoking (PY), BMI and severity of chronic obstructive pulmonary disease (COPD) according to the Global Initiative for Chronic Obstructive Lung Disease (GOLD)^20^— showed no significant correlations in the tumour tissues (Supplementary Fig. S7).

### The lipidomes of tumour-free alveolar tissues are mostly affected by emphysema, age and inflammation

**Figure 5:**
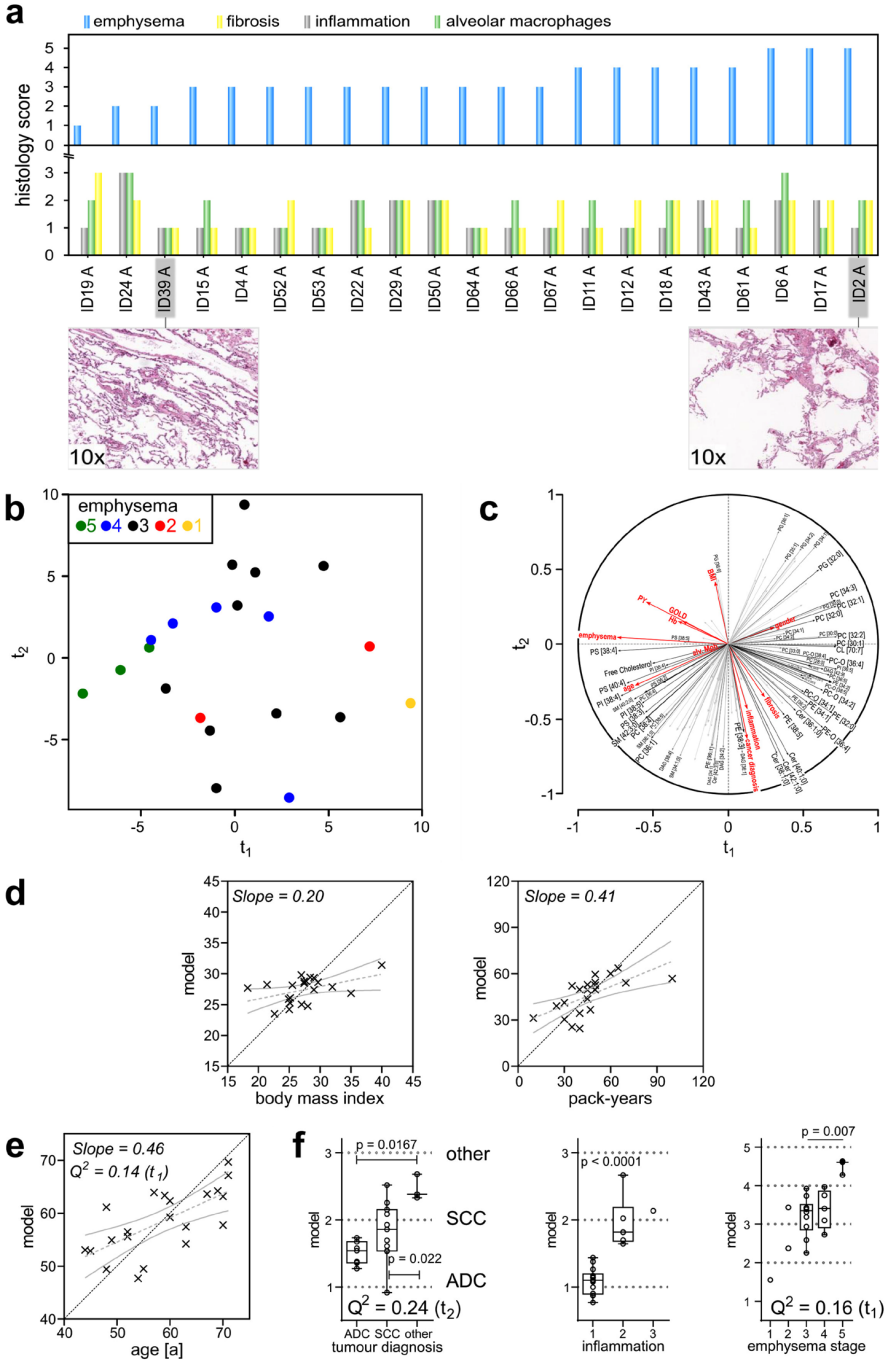
Lipidomes of alveolar tissues correlate with patient data and histological scores. (**a**) Histology scores for alveolar control tissues. Alveolar control tissues were scored by inflammation (stages 0, 1, 2, 3), alveolar macrophages (stages 0, 1, 2, 3), emphysema according to Nagai *et al*.^37^ and fibrosis (stages 0, 1, 2, 3). The bar graphs show scoring results for every individual tissue, sorted according to increasing emphysema score. (**b**) PLS regression factor map for alveolar tissue lipidomes. Individuals were colour coded according to their emphysema score. (**c**) Correlation of lipid quantities, histology scores and clinical data to the t-components. Predictor variables (X) are indicated by black vectors and responses (Y) by red vectors. (**d**) Correlations of the PLS regression model between original values and computed values for BMI and PY of patients. Grey dotted lines represent the linear fit, and solid grey lines indicate 95% confidence bands. The dotted black line indicates the location of the ideal correlation. (**e**) Evaluation of the association between age and lipidome. (**f**) Evaluation of categorical parameters: tumour diagnosis, inflammation status and emphysema stage. Q^2^ values are the results of the cross-validation of the model. A model is considered significant for a response if Q^2^ ≥ 0.0975. The associated t-component is noted in brackets after the Q^2^ value. All p values were calculated using Mann-Whitney U test. Q^2^ values and p values are only noted when significance was reached.

The results for the PLS regression analysis of tumour tissues showed that histological phenotypes are mirrored in the lipidomes. We then applied the same method to investigate the influence on lipidomes in alveolar lung tissues. Alveolar control tissues exhibited a much more homogeneous histology. The content of cell types did not change to the extent observed in the tumours. Specific attention was given to the emphysema score because of its specific role in COPD. Accordingly, the emphysema stage was used to align all other tissue phenotypes (Fig. 5a). The extent of histological changes, an increase in the size of alveoli, is clearly visible from the examples shown for stage 2 (ID 39 A) and stage 5 (ID 2 A) (Fig. 5a). Although inflammation status, fibrosis and presence of alveolar macrophages were also scored, we did not observe a correlation between progressing emphysema and the other histological scores (Fig. 5a).

We calculated the PLS regression model using 139 lipids that were present in every alveolar tissue sample and included all histopathological and clinical parameters (Supplement 3). First, we focused on the impact of the emphysema grade and ageing on the lung lipidome. The PLS regression factor map showed an inverse relationship between the emphysema score and component t_1_ (Fig. 5b and Supplement 6). However, a closer look at the correlation circle revealed that emphysema and age correlated with each other (Fig. 5c). In these samples, the abundance of glycerophospholipids PS [38:4], PI [38:4], PI [38:5] and PS [40:4] was strongly correlated with increasing emphysema scores and ageing. We also observed that the distribution of standard coefficients of the model, linking lipid data and phenotypes, was very similar for emphysema and age (Supplementary Fig. S8 and Supplement 6). Furthermore, the cross-validation for both the influence of emphysema (Q^2^ = 0.16) and ageing (Q^2^ = 0.14) on the lipidome was significant (Fig. 5e,f). Although the potential effects of emphysema and age on the lipidomes could not be completely separated, we showed that a systematic change in the lipidome is detectable for both. This finding is in line with earlier reports that ageing and progression of emphysema are connected^21^. In contrast, the influence of BMI and PY on the lipidome was rather limited (Fig. 5d). Furthermore, it was possible to distinguish inflammation stage 1 from stage 2 using lipidome data (Fig. 5f). Here, we observed the strongest correlation for DAG [34:2] and DAG [34:1], and for 10 TAG species, with positive standard coefficients (Supplement 6). On the other hand, strong negative coefficients were computed for long-chain SMs such as SM [38:1], SM [40:1] and SM [40:2], as well as for PG [35:1], PG [36:1] and PG [33:1].

Our data further indicated a connection between the histological subtype of cancer and the lipidome of the corresponding tumour-free control tissues (Fig. 5f). This result was somewhat unexpected and might indicate that there is a limit to using residual tissue material of cancer patients as a model. In particular, the three patients diagnosed with large-cell carcinoma and carcinoid tumour (for this study grouped under ‘other’) were distinguished from those with ADC (p = 0.0167) and SCC (p = 0.022) using the PLS regression model. However, a potential overlapping effect of tumour diagnosis and inflammation and/or fibrosis cannot be excluded. Another possible association could be smoking behaviour. SCC is predominantly linked to smoking^22^, whereas ADC has a high prevalence in never-smokers^23^. The impact of gender was relatively low but could indirectly influence this finding because of the relatively small number of female patients included and the underlying bias toward ADC (Supplementary Fig. S7).

For the remaining categorical variables—fibrosis, haemoglobin content (as measure of remaining blood in the tissue) and GOLD stage—no further trends were observed (Supplementary Fig. S7).

## Discussion

Previous studies describing lung tissue lipidomes strictly focused on phospholipids accessible by triple quadrupole specific scans and did not describe PGs and PAs or neutral lipids^8, 19^. In contrast, the lipidomic screens we applied overcame the problem that PG and PA do not form characteristic fragments or neutral losses related to the lipid head group^24, 25^. Our analytical approach covered a wide range of phospholipid classes and enabled quantification of neutral lipids. As a result, we improved coverage of the human lung lipidome, and we provide these findings as a general resource (Supplement 3).

Our research shows that the major abundant structural lipids alone are sufficient to distinguish tumour tissues from alveolar control tissues. By showing the remarkable changes of PG, TAG and CE lipids, we have significantly improved the understanding of lipid metabolic perturbations of NSCLC. In particular, PG molecules are of special interest, especially in alveolar tissues, as they are the main constituents of pulmonary surfactant^4^. Among the single lipids most characteristic of NSCLC, we identified CE [18:1], CE [20:4] and TAG [54:4], which are all involved in energy storage and cholesterol metabolism. These nicely reflect the enhanced energy consumption by malignant tumours. It is little wonder that we found a high abundance of PG [36:1], PG [34:1], and PG [34:2] in tumour-free alveolar lung tissues, all of which are constituents of pulmonary surfactant. Hence, lipid profiles of alveolar lung tissues comprised elevated levels of surfactant lipids, including saturated PC and a variety of PG species that are generally decreased in NSCLC. At the same time, tumour tissues showed an increased abundance of neutral lipids such as TAG and CE, which further correlated with the fraction of necrotic areas. With the help of PLS regression, we correlated specific lipid panels to the percentage of vital tumour, necrosis and stroma. This shows that the cellular composition of tumour tissues should be considered to improve the diagnostic and functional value for such analyses. It is likely that metabolic active tumour cells induce a large proportion of lipidome perturbations, which allows the clear differentiation between tumour and alveolar control tissues. In almost all cases, the loss of surfactant lipids indicated pathological changes, as well as a loss of function for the alveoli. Using this perspective, we showed that scores derived from lipidome data are not only suitable to discriminate between tumour tissues and tumour-free alveolar control tissues, but also between the major NSCLC subtypes ADC and SCC. This not only opens the opportunity to provide a novel diagnostic approach for cancer differentiation, but may also enable the study of functional aspects of different carcinogenic mechanisms. It is also noteworthy that the metabolic reprogramming of tumour cells leads to systematic differences in lipidome composition, which we highlighted with the LUX score as a measure of lipidome homology^18^.

From our results, we can extrapolate that further improvements in lipid quantitation, as well as sampling technology for examining tissue lipidomes, will be necessary to gain further insights into the functional aspects of the observed histopathology. Quantitation procedures for many glycerophospholipids, major abundant sphingolipids and neutral lipids will improve because better suitable standards are increasingly commercially available. However, for now it remains a challenge to use quantitative tissue lipidomics for clinical applications because of the technological demands, costs for such analyses and lack of automation procedures.

Furthermore, the isolation of defined cell populations from tissues using fractionation techniques might provide a better opportunity to standardize sampling^26^. Another possibility would be to apply sophisticated localisation-resolving techniques to isolate cells and tissues, such as laser capture microdissection^27, 28^. Tissue substructures and cell populations that are the focus of pathological events such as tumorigenesis^8^, development of granulomas or chronic inflammation can be further analysed by mass spectrometric imaging^29, 30^.

The informatics and statistical methodology to correlate histological phenotypes and clinical parameters to lipidome data will be a central aspect for further investigations. Linear models based upon PLS regression will be a valuable tool. However, other approaches based upon machine learning and nonlinear models^31, 32^ should be evaluated, in conjunction with increased case numbers. Besides computational improvements, translational and interdisciplinary cooperation to incorporate other omics’ results will be intensified. Such comprehensive study designs will help to refine molecular phenotyping and aid the implementation of personalised medicine concepts for lung diseases.

Together with the observed histopathological phenotype, we assessed the information value and success of our data interpretation strategy upon the degree of expected lipid metabolic perturbations. Perturbations occurring in tumours have such a strong impact that lipid panels signifying these morphological and functional changes can be identified with ease. The differentiation of tumour subtypes required the application of multivariate analysis tools to identify diagnostic lipid markers, even in the background of other underlying histological differences. For the association of lipid metabolic consequences for ageing, emphysema stage and inflammation, model-based approaches are a necessity. We are convinced that the applied PLS regression methodology fosters such sophisticated investigations.

The molecular mechanisms behind the development and prevention of emphysema are not yet fully understood. A few studies in animal models^33–35^ have related emphysema to changes in Cer metabolism. We could not confirm this observation, but did find evidence for age dependency of the lung lipidome, in line with the hypothesis that ageing is a known risk factor for developing emphysema and COPD^21^.

In conclusion, we have described the lipidome of tumour-free alveolar lung tissues and shown the impact of reprogrammed lipid metabolism in tumours. With the help of PLS regression, a linear model can be created to relate lipidome alterations to histopathological phenotypes and clinical parameters. The resource presented here provides a first glimpse of the human lung lipidome in the context of disease development and natural variations.

## Methods

### Chemicals and lipid standards

All solvents, reagents and lipid standards were used in the highest available purity grade (see details in Supplementary Methods, Supplement 7).

### Cohort

Human lung tissue samples were collected from March to October 2013. Samples originated from lung cancer patients after surgical tumour removal at the LungenClinic Großhansdorf, Germany. This study was conducted using material from NSCLC resections and performed anonymously. The use of patient tissue and all experimental procedures were approved by the local ethics committee of the University of Lübeck (AZ 12–220). All procedures were carried out in accordance with respective guidelines and regulations. The clinical parameters of the 26 lung cancer patients are summarised in Table 1. Forty-three human lung tissue samples from the patients were analysed. Twelve patients had SCC, 11 had ADC, and patients with large-cell carcinoma (ID19), sarcomatoid large cell carcinoma (ID43) and carcinoid tumour (ID52) were grouped under ‘other cancer’. Age ranged from 44 to 71 years. Clinical characteristics such as PY, GOLD stage and BMI were included in our analysis.

### Sampling strategy

When available, representative biopsy samples of tumour-containing and tumour-free lung parenchyma were collected in the range of 0.5 to 1 g wet-weight. All samples were divided into two approximately equal-sized parts for processing for histopathology analyses and shotgun lipidomics. For shotgun lipidomics, samples were shock-frozen in liquid nitrogen and stored at −80 °C until analysis. For histological characterisation, tissue samples were fixed using the HOPE technique (HEPES-glutamic acid buffer-mediated organic solvent protection effect)^36^.

### Histopathology

Tissue sections of 1 µm were prepared from paraffin-embedded blocks and stained with haematoxylin and eosin. Slices were characterised by light-microscopy. Phenotypic features were scored according to the specific tissue type. For tumour-free tissues, emphysema, inflammation, alveolar macrophages and fibrosis were scored on a scale from 0 to 3. A score of 0 would represent no inflammation/fibrosis/macrophages and a score of 3 would indicate a high degree of inflammation/fibrosis/macrophages. The emphysema grade was determined, according to the method by Nagai *et al*.^37^ in grades between 0 (no emphysema) and 10 (complete loss of alveolar structure). Tumour-containing tissues were characterised by their relative content of vital tumour, stroma and necrosis, defined as tissue fraction given in percentage. Additionally, inflammation was scored in stages between 0 and 3. Tissue scans were recorded with a Plustek OpticsLab H850 scanner and the pictures can be found in Supplement 8.

### Lipid extraction

Tissue samples were homogenised in 50 mM KCl buffer using an Ultra-Turrax Tube Drive (IKA, Staufen, Germany). A constant ratio between tissue wet-weight and buffer volume of 1:20 (wt/vol) was applied for all samples. Accordingly, 300 μL tissue homogenate, containing 14.4 mg tissue, was extracted using the MTBE-based lipid extraction procedure^38^ (see details in Supplementary Methods, Supplement 7).

### Shotgun lipidomics screen

Lipid extracts were tenfold diluted in chloroform/methanol/2-propanol (1:2:4; v/v/v) using 3.7 mM ammonium acetate as additive for positive ion mode and 0.05 mM ammonium chloride for negative ion mode. Lipidomics screens^10^ were performed using an Apex Qe Fourier Transform Ion Cyclotron Resonance mass spectrometer (Bruker, Bremen, Germany) equipped with a TriVersa NanoMate (Advion BioSciences, Ithaca, NY, USA) as autosampler and ion source. Mass spectrometric acquisitions of 5 minutes’ time were separately recorded for the positive and negative ion mode (see details in Supplementary Methods, Supplement 7).

### Quantification of free cholesterol

Free cholesterol was quantified using derivatisation with acetyl chloride, as described earlier^39^, using deuterated cholesterol as internal standard (see details in Supplementary Methods, Supplement 7).

### Lipid identification and quantification

LipidXplorer^40^ was used to identify the lipid classes—TAG, SM, lysophosphatidylserine (LPS), PS, lysophosphatidylglycerol (LPG), PG, lysophosphatidylethanolamine (LPE), PE, PE-*O*, PA, lysophosphatidylcholine (LPC), PC, PC-*O*, lysophosphatidylinositol (LPI), PI, cardiolipin, CE, Cer and hexosylceramide—using peak lists as input (Supplement 9) and customised MFQL scripts (Supplement 10). Lipids were assigned only when the mass accuracy was ≤2.5 ppm and the signal intensity was at least 10 times higher than that of blank control samples. Afterwards, the lipid identification was revised in respect of isobaric species by evaluating isotopic distribution.

Lipid species were annotated as follows: lipid class [no. of carbon atoms in aliphatic chains:no. of unsaturations in aliphatic chains]. For sphingolipids: class [no. of carbon atoms in aliphatic chains:no. of unsaturations in aliphatic chains; no. of additional hydroxylations: 0 or 1]. Lipid species of PE and PC with one *O*-alkyl chain and one *O*-acyl link are indicated as PE-*O* and PC-*O*, respectively.

Lipids species were quantified based on responses of internal standards (Supplementary Table S10). LPC, PC, PE, SM and TAG species were quantified based on their respective internal standard from the same class. For other lipid classes, abundances were determined in relation to the sum-intensity of internal standards LPC, SM, PC, and TAG in positive ion mode as well as LPC, PC, and PE in negative ion mode (see details in Supplement 7 and Supplementary Table S11)^17^. Finally, the relative abundance for all lipids was determined as a percentage of total abundance of all lipids (Supplement 3).

### Multivariate data analysis methods

For the PCA and hierarchical cluster analysis presented in Figs 2, S4 and S5 lipid species were included that were present in at least 90% of the samples (Supplement 3). PCA was performed using the package FactoMineR^41^ of R (The R Project, version 3.2.0^42^). Hierarchical clustering was computed applying Euclidean distance and complete linkage using Gene Cluster 3.0^43^ and visualised by Java Treeview-1.1.6r4^44^ (Supplement 11).

Tissue discrimination scores based on lipid panels presented in Fig. 3 were calculated for (i) tumour tissues from alveolar control tissues; (ii) ADC from SCC.

(i) To determine the lipid panel for differentiation of alveolar control tissues and tumour tissues, only lipid species that were detected in all 43 samples were included. As a result, 20 lipids were chosen that were significantly changed between tumour and alveolar control tissues with p > 0.01 (two-tailed t-test) and comprised a log2-fold change of <−1 or >1 (Supplement 3).
(ii) For discriminating ADC and SCC tissues, a lipid panel was chosen from lipids present in each tumour tissue sample. A panel of 7 lipid species was chosen with p < 0.05 (two-tailed t-test), which showed a log2-fold change of <−1 or >1 between the two tumour types (Supplement 3).

To calculate both scores, the relative abundance of chosen lipids was log2 transformed and mean centred. Finally, both scores were calculated according to equation 1.

1 $$S=\sum _{i}A\cdot {c}_{i}$$

*S*: Score

*A*: constant: A = 1 if log2-fold change >1; A = −1 if log2-fold change <−1

*c*_*i*_: log2 transformed and mean centred abundance of lipid *i of the respective panel*.

The associated ROC curves and volcano plots were calculated and visualised by GraphPad Prism 6 (GraphPad Software, Inc., La Jolla, CA, USA).

To calculate a mathematical model that correlates histological and clinical data to the lipidome, we used PLS regression, as described by Wold *et al*. and Geladi and Kowalski^13, 14^. PLS regression was calculated using the *R* package *plsdepot* ^45^ using the function *plsreg2*. Prior to PLS regression, clinical data and histopathology scores (defined as responses; Y variables) and all lipid quantities (defined as predictors, X variables) were transformed to consist only of values between 0 and 1. Categorical variables, like gender or tumour diagnosis, were also scaled in a range of 0 to 1.

For the PLS regression model of tumour tissues presented in Fig. 4, the complete set of response variables was used as input. Furthermore, only lipids that were quantified in all tumour tissue samples were included in the analysis (Supplement 3).

The PLS regression model of alveolar lipidomes shown in Fig. 5 was computed using all available histological scores (emphysema, fibrosis, inflammation, alveolar macrophages) and clinical data (age, gender, tumour diagnosis, BMI, PY, GOLD stage) as response variables. Only lipids that were present in all alveolar control tissues were considered, as listed in Supplement 3.

Cross-validation was performed using the default *leave*-*out*-*one* algorithm of the R function *plsreg2* of the *plsdepot* R package^13^. Briefly, the dataset is split randomly into ten equally sized segments consisting of individual patients’ data. Nine of these segments are used as training set to compute the model, while the remaining segment is used for validation. This process is repeated so that all ten segments are once utilised for validation. The results of the cross-validation are expressed by the Q^2^ value. A model for a response variable is considered to be significant when at least one Q^2^ value for one principal component is ≥0.0975^45^.

### Chemical space model and LUX score

Chemical space model and LUX scores were calculated as described previously^18^. The source code to calculate LUX scores can be downloaded from the website http://lux.fz-borstel.de. The overall fatty acid composition of the lung lipidome was determined by tandem mass spectrometry, performed in negative ion mode on a Q Exactive Plus Orbitrap mass spectrometer (Thermo Scientific, Bremen, Germany). We used the resulting list of 35 fatty acids to infer isomeric species for all identified lipids and to choose a representative molecule (Supplementary Table S12 and Supplement 7). From the initially identified 311 lipids, 293 lipids were used for LUX score calculation (Supplement 3). Eighteen lipids could not be computationally generated using the presented fatty acids as minimum building blocks. Hierarchical clustering was performed using the R function *hclust* with Euclidian distance and complete linkage. PCA of lipids based on structural similarity was performed with R function *princomp*. Error modelling was performed using quantification threshold (d_t_) and standard deviation (SD) of (1) t = 0.005, SD = 0.002 and (2) t = 0.003, SD = 0.001.

## Electronic supplementary material

Supplement 1

Supplement 2

Supplement 3

Supplement 4

Supplement 5

Supplement 6

Supplement 7

Supplement 8

Supplement 9

Supplement 10

Supplement 11
