# Supplementary material for: Lipidomes of lung cancer and tumour-free lung tissues reveal distinct molecular signatures for cancer differentiation, age, inflammation, and pulmonary emphysema
Source: Sci Rep. 2017 Sep 11;7:11087. doi: 10.1038/s41598-017-11339-1 (PMC5594029; doi:10.1038/s41598-017-11339-1)
Supplement: Supplementary file 1 — Supplement 1 [file 41598_2017_11339_MOESM1_ESM.pdf]

# Supplement 1: Supplementary Figures and Tables

## Lipidomes of lung cancer and tumour-free lung tissues reveal distinct molecular signatures for cancer differentiation, age, inflammation, and pulmonary emphysema

Lars F. Eggers[1], Julia Müller[2], Chakravarthy Marella[1], Verena Scholz[1], Henrik Watz[3,4], Christian Kugler[5], Klaus F. Rabe[4,5], Torsten Goldmann[2,4,#] & Dominik Schwudke[1,4,#,*]

[1]Research Center Borstel, Bioanalytical Chemistry, Parkallee 1-40, 23845 Borstel, Germany.

[2]Pathology of the University Hospital of Lübeck and the Research Center Borstel, Clinical and Experimental Pathology, 23845 Borstel, Germany.

[3]Pulmonary Research Institute at LungenClinic Großhansdorf, Wöhrendamm 80, 22927 Großhansdorf, Germany.

[4]Airway Research Center North, German Center for Lung Research, Wöhrendamm 80, 22927 Großhansdorf, Germany.

[5]LungenClinic Großhansdorf, Wöhrendamm 80, 22927 Großhansdorf, Germany.

#Shared senior authorship.

*Corresponding author: dschwudke@fz-borstel.de

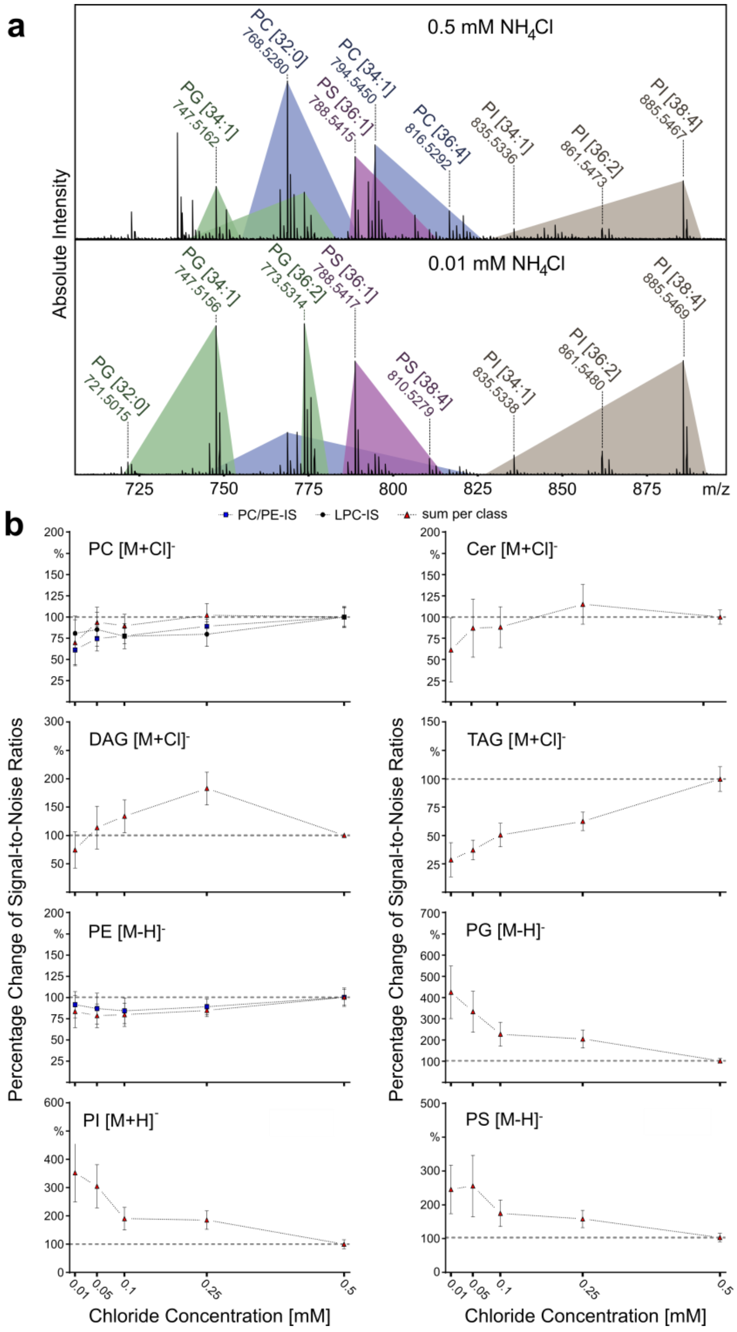


**Supplementary Figure S1.** Sensitivity changes with $NH_4Cl$ concentration. (**a**) Fourier Transform Ion Cyclotron Resonance (FT-ICR) mass spectra in negative ion mode using 0.5 mM (top) and 0.01 mM (bottom) $NH_4Cl$ as additive in the spray solution. Signals from PC ion species are illustrated in blue, PG in green, PS in purple, and PI in brown. (**b**) Plot of the percentage change of signal-to-noise ratios (S/N) as a function of the $NH_4Cl$ concentration as electrospray ionisation (ESI) additive. The percentage change of S/N was calculated as the sum of S/N of individual lipids for a given class. These values

were normalised to their average response at the additive concentration of 0.5 mM $NH_4Cl$. Lipid classes: PC (19 species), Cer (12 species), DAG (9 species), TAG (3 species), PE (9 species), PG (17 species), PI (11 species), and PS (10 species), including S/N for internal standards of PC, LPC and PE. The y-axis labels appear only once per column of the four subpanels.

We aimed to establish a rapid screening procedure to identify as many lipid species within one analytic run as possible. $NH_4Cl$ concentrations were optimized to increase sensitivities for lipid classes that poorly form positive ions (PI, PS, PG, and PA). As examples, negative ion mode ESI-FT-ICR mass spectra acquired with an additive concentration of 0.5 mM and 0.01 mM $NH_4Cl$ are provided in panel **a**. The most prominent changes were observed in the S/N between PC (blue) and PG (green). The sensitivities for certain lipid classes changed with the additive concentration. Specifically, the chloride adduct ions ($[M+Cl]^-$) of PC, DAG and TAG showed decreasing S/N at the low additive concentration. The opposite trend was observed for the deprotonated ions of PG, PI and PS. Hence, all analyses in the negative ion mode were performed with 0.05 mM $NH_4Cl$, because PG and PC lipids showed sufficient sensitivities.

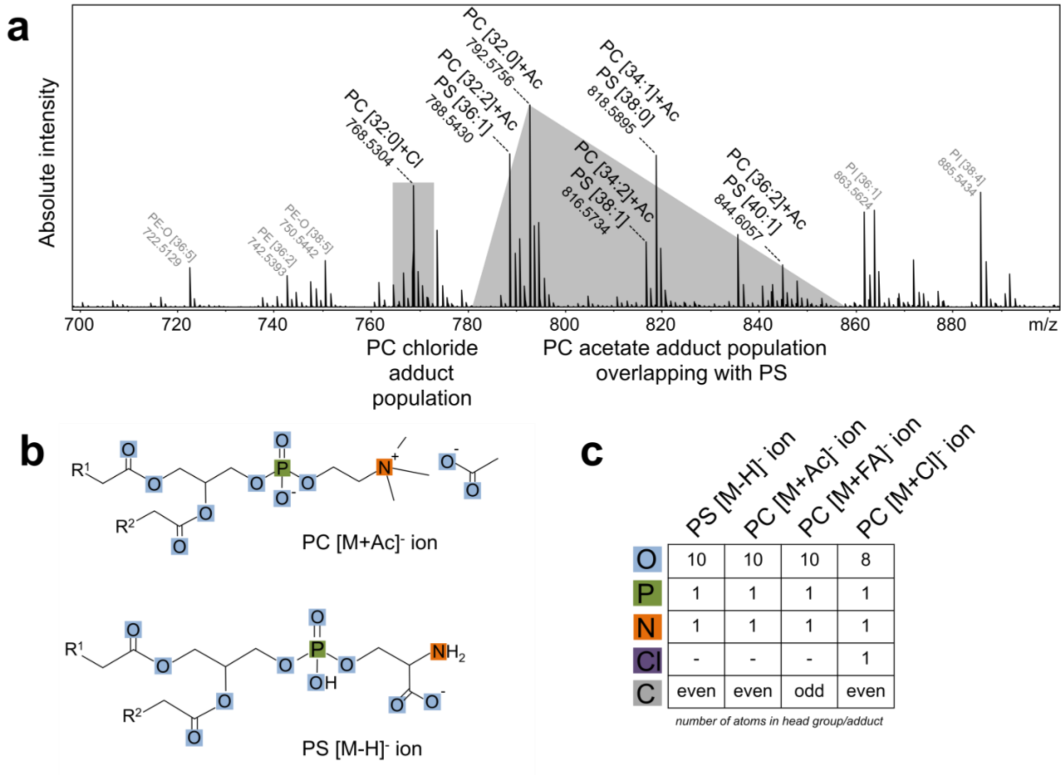


**Supplementary Figure S2.** Overlapping mass spectrometric signals of PC and PS using ammonium acetate as electrospray ionisation (ESI) additive. (**a**) Survey mass spectrum showing overlapping signals of PC acetate adducts with PS anions. (**b**) Chemical structures of PC and PS ions that have the same chemical sum composition. (**c**) Heteroatom sum composition of PS anions; PC acetate adducts, $[M+Ac]^-$; PC formate adducts, $[M+FA]^-$; and PC chloride adducts, $[M+Cl]^-$.

For mass spectrometric lipid analyses, ammonium acetate is a commonly used salt additive to support ionization processes.[1-3] In positive ESI, ammonium cations form adducts with neutral lipids (e.g., TAG, DAG, CE), which enables mass spectrometric detection of these species. In negative ESI, acetate adducts are formed with lipids containing a choline head group, such as SM and PC, as well as ceramides or neutral lipids (DAG, TAG). Nevertheless, there are disadvantages to use ammonium acetate in lipidomics screens. Here, lipid species are identified by their accurate mass, which permits calculation of the chemical sum composition of an ion species.[1] Many ion species of PC acetate adducts have the same mass as deprotonated PS, since they share the same heteroatom composition (panels **b** and **c**).

A salt additive with a different anion could avoid this problem. The use of formate adducts results in the same heteroatom composition as that for acetate adducts '$O_{10}PN$'.[4] However, the total number of carbon atoms would differ by one, allowing independent quantification of PC and PS in mammals under the assumption that only even-numbered fatty acids are present (panel **c**). When using $NH_4Cl$ as an additive, the heteroatom composition of PC changes to '$O_8PNCl$' (mass difference from PS = 2.979 Da), allowing independent quantification of all PC species. The stability of the quantitation can further be improved because competition between residual chloride anions from the extraction and the acetate additive is completely avoided. As an example, a spectrum in which the acetate adduct and the chloride adduct of PC [32:0] are simultaneously detected is shown in panel **a**.

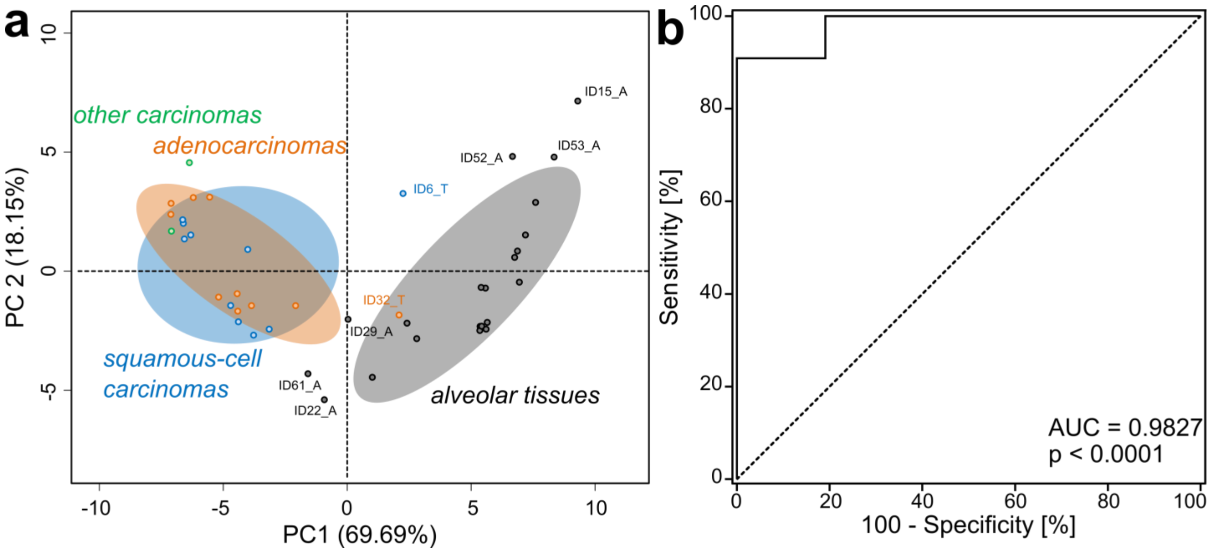


**Supplementary Figure S3.** Distinguishing tumour-free and tumour-containing tissues by LUX scores. (**a**) PCA factor map showing principal component 1 (PC1) and principal component 2 (PC2) computed with the R package *FactoMineR*. Tumour-free alveolar tissues are marked in grey, adenocarcinomas in orange, squamous cell carcinomas in blue and other carcinomas in green. The highlighted area includes all data points of a cluster with 68% confidence. (**b**) The ROC curve is based on PC1 as predictor. The data show a clear separation of tumour-free alveolar tissues and tumour tissues.

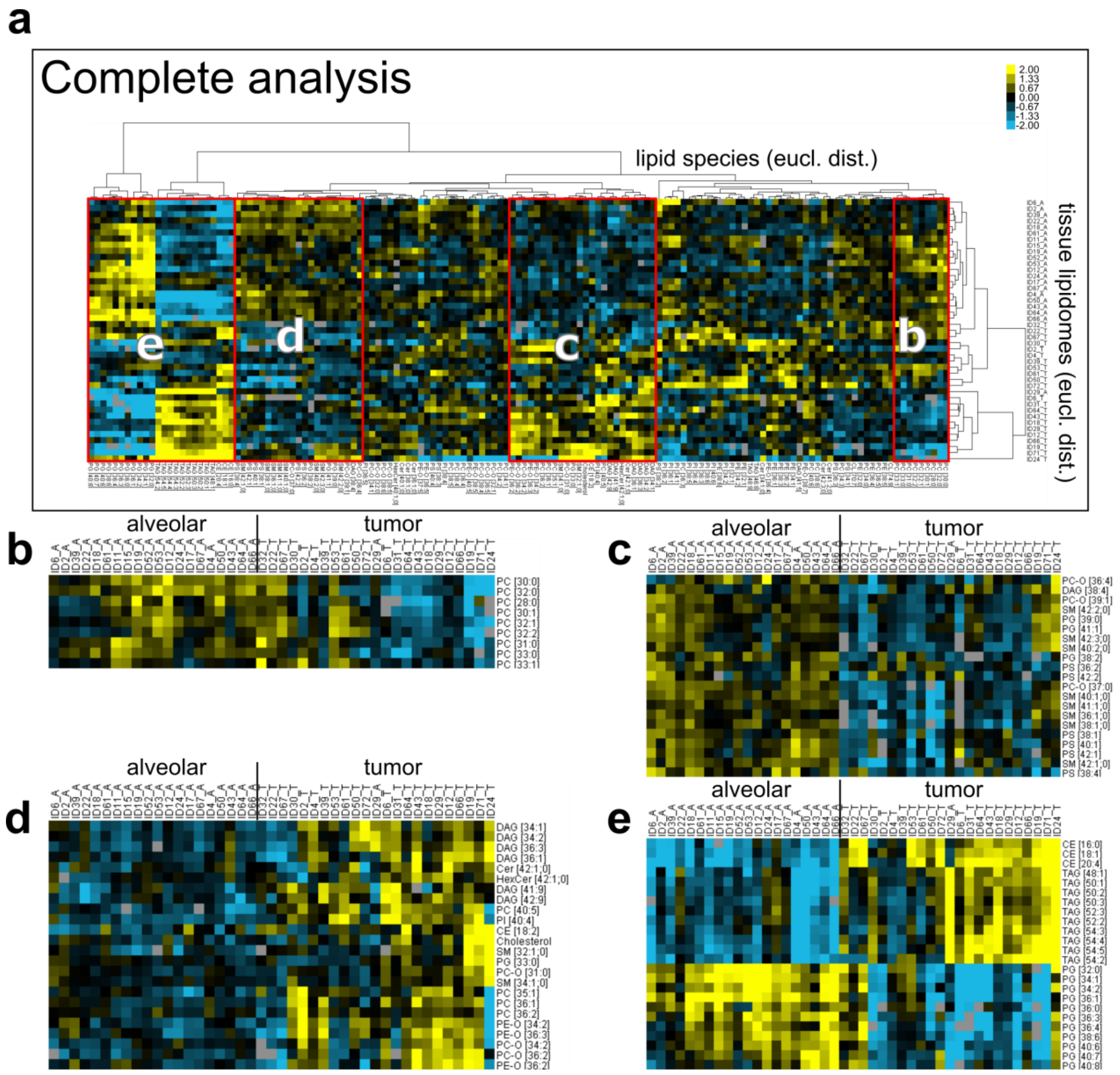


**Supplementary Figure S4.** Results of the hierarchical clustering of all lung tissue lipidomes. (**a**) Heat-map representation of 141 lipid species found in 43 lung tissues. Normalized lipid abundance is shown in the colour code. Yellow marks an increase compared to the overall mean; blue marks a decrease. Tissue samples and lipid species (tree not shown) were clustered using Euclidean distance and complete linkage. (**b – e**) Zoom in on selected lipid panels indicated in (**a**).

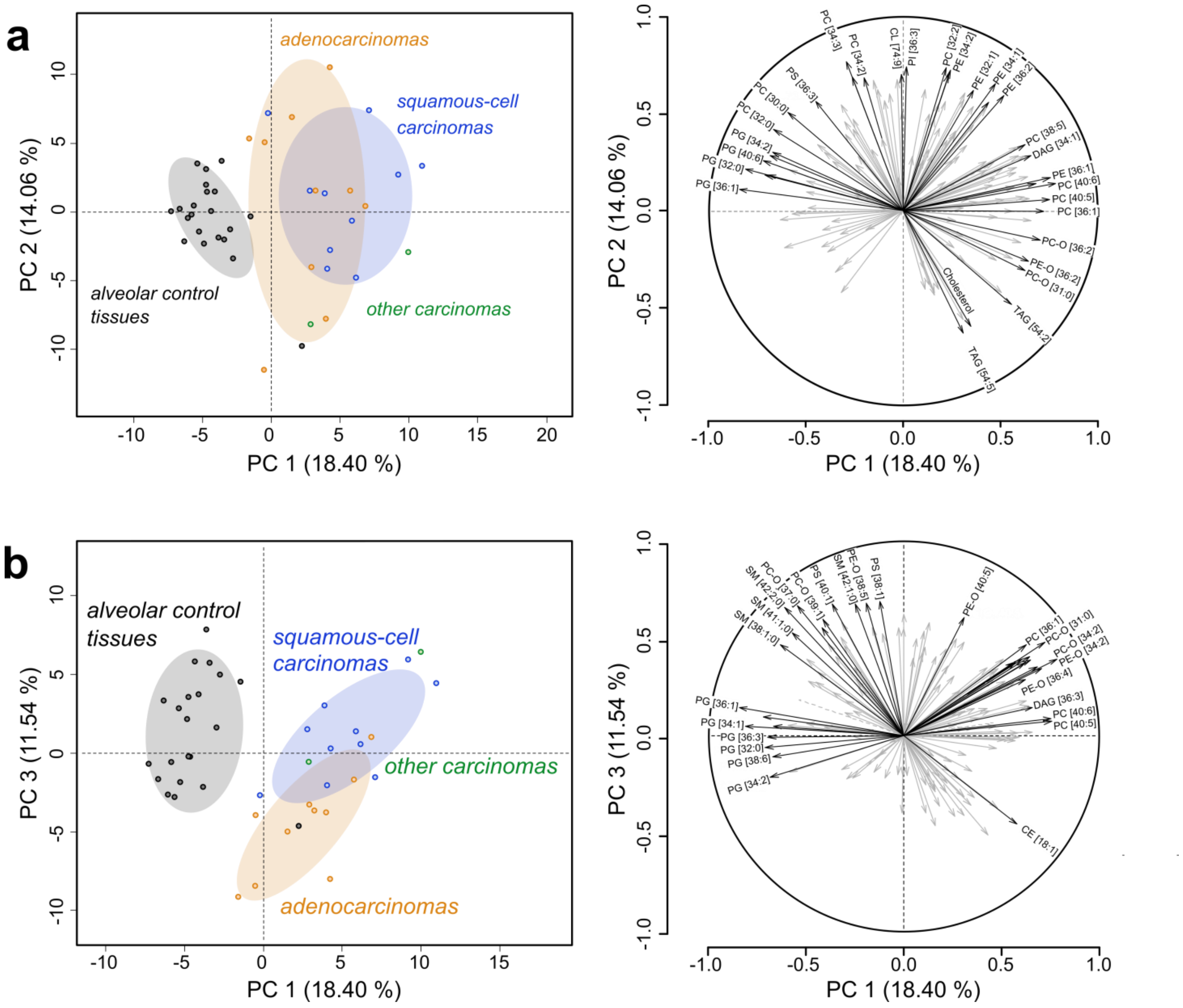


**Supplementary Figure S5.** PCA of all lung tissue lipidomes. (**a**) Factor map (PC 1, PC 2). The same set of 141 lipids was used as presented for hierarchical cluster analysis in Fig. 2. Each point on the map represents one tissue lipidome. Tumour-free tissues are marked in grey, adenocarcinoma tissues in orange, squamous cell carcinoma tissues in blue and other carcinoma tissues (e.g., large cell carcinoma) in green. The highlighted areas represent all data points of the cluster within 68% confidence. Correlation circles of variables (lipid abundance) to the principal components are shown. Only the longest vectors are assigned; all other lipid species are indicated as grey vectors in the background. (**b**) Factor map and correlation circle (PC 1, PC 3).

Supplementary Table S6: AUCs for lipids differentiating ADC and SCC.

| Lipid | AUC |
|---|---|
| Cer [34:0;0] | 0.855 |
| Cer [42:1;0] | 0.800 |
| PC [36:1] | 0.790 |
| PE-*O* [34:2] | 0.770 |
| PE-*O* [36:3] | 0.729 |
| PE-*O* [36:4] | 0.835 |
| PI [36:1] | 0.860 |

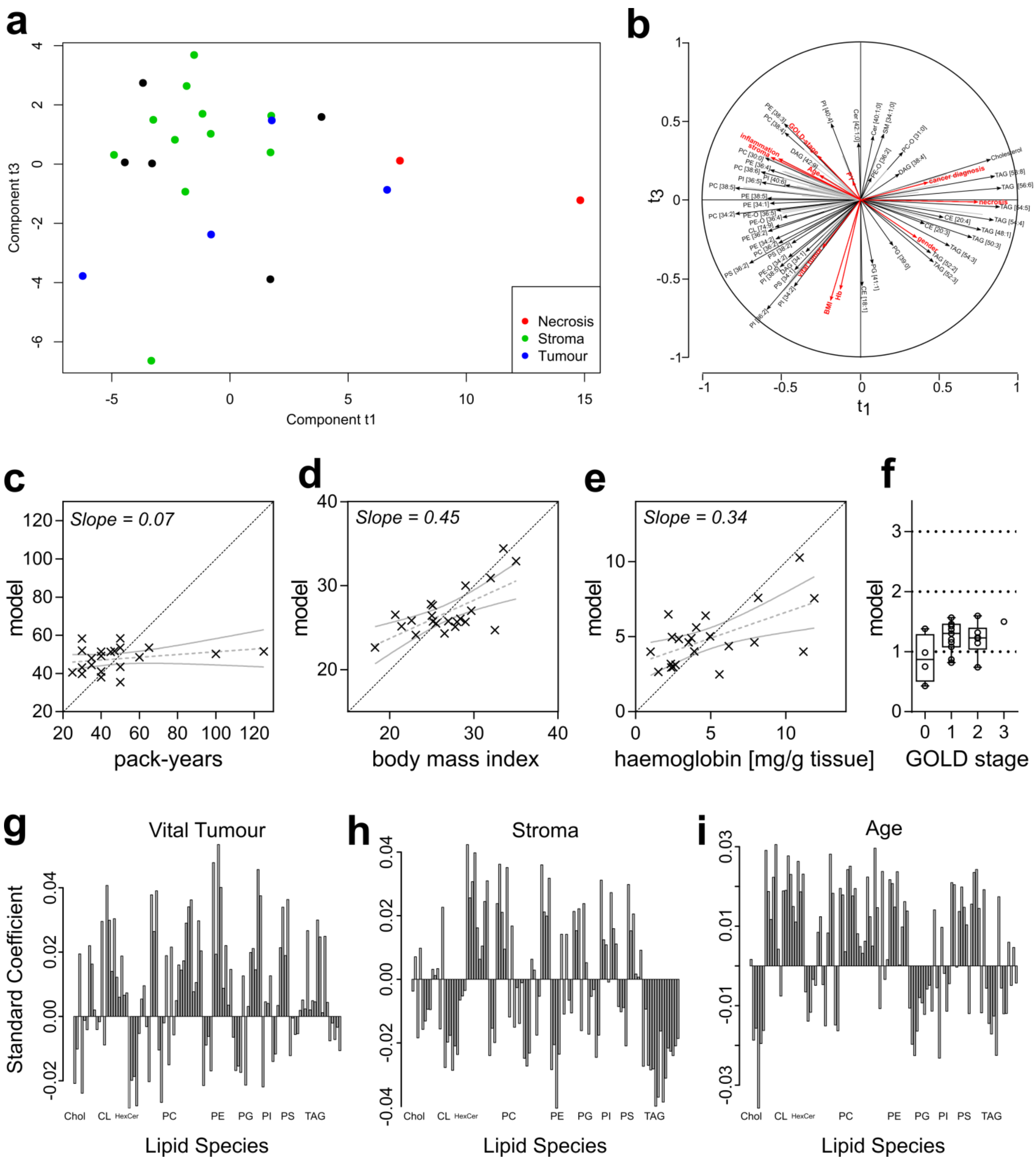


**Supplementary Figure S7.** Additional information for Figure 4. PLS regression of tumour tissue lipidomes. (**a**) Scores of components t1 vs. t3. (**b**) Correlation between the variables and the components t1 and t3. (**c–f**) Correlation of original scores to predicted scores by the PLS regression for pack-years (**c**), body mass index (**d**),

haemoglobin content (**e**),  and GOLD stage (**f**) from the analysis shown in Figure 4. (**g–i**) Standardised regression coefficients for the PLS regression models of vital tumour, stroma and age. The regression coefficients show the contributions of various lipid species to the model (see Supplement 6).

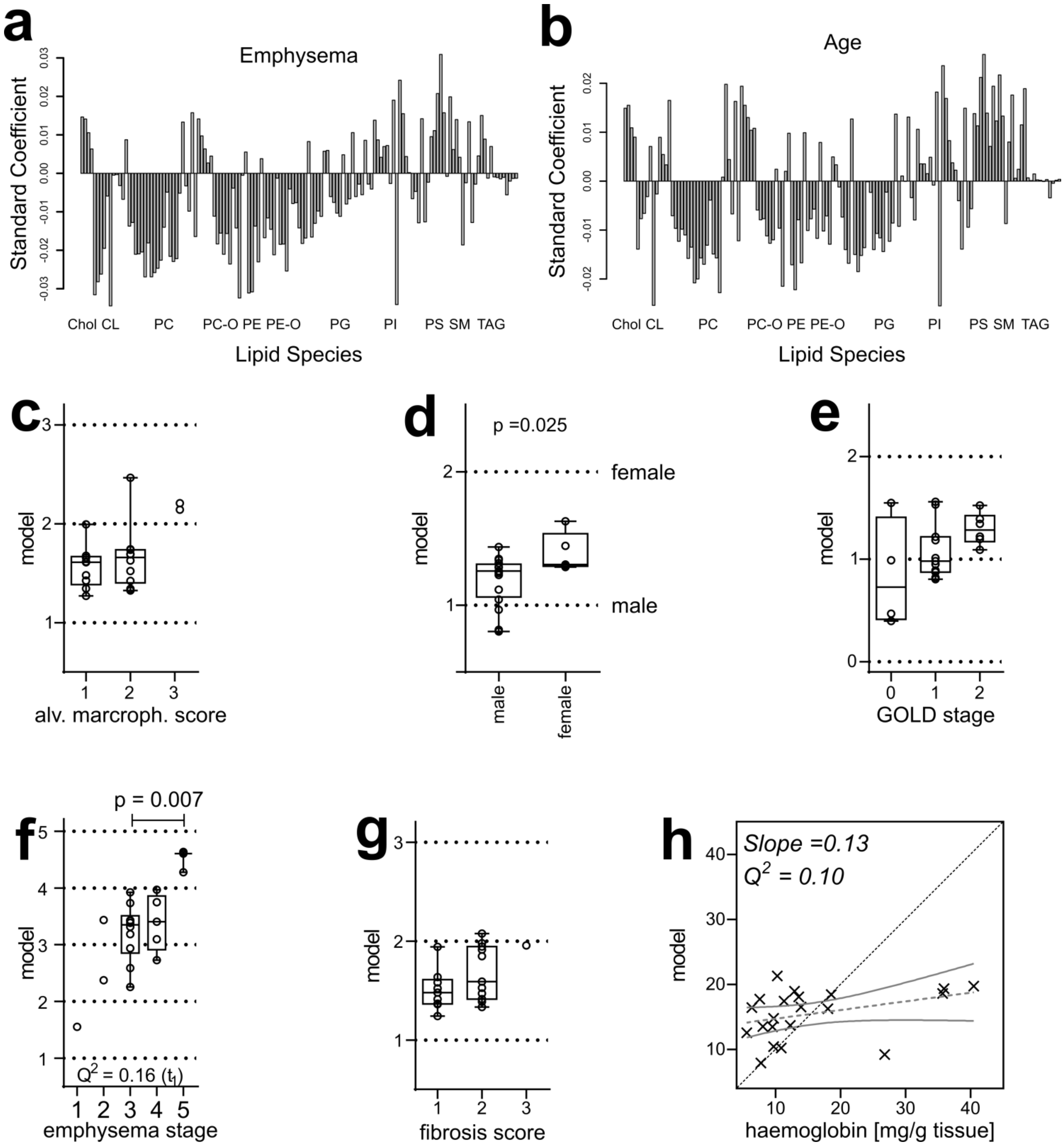


**Supplementary Figure S8.** Additional information for Figure 5. PLS regression of alveolar control tissue lipidomes. Bar plot of standardised regression coefficients for (**a**) emphysema and (**b**) age (see Supplement 7). The regression coefficients show the contribution of specific lipid species to the model. Correlations for original responses to

the PLS model for (**c**) alveolar macrophages, (**d**) gender, (**e**) GOLD stage, (**f**) emphysema, (**g**) fibrosis and (**h**) haemoglobin content determined from the homogenate.

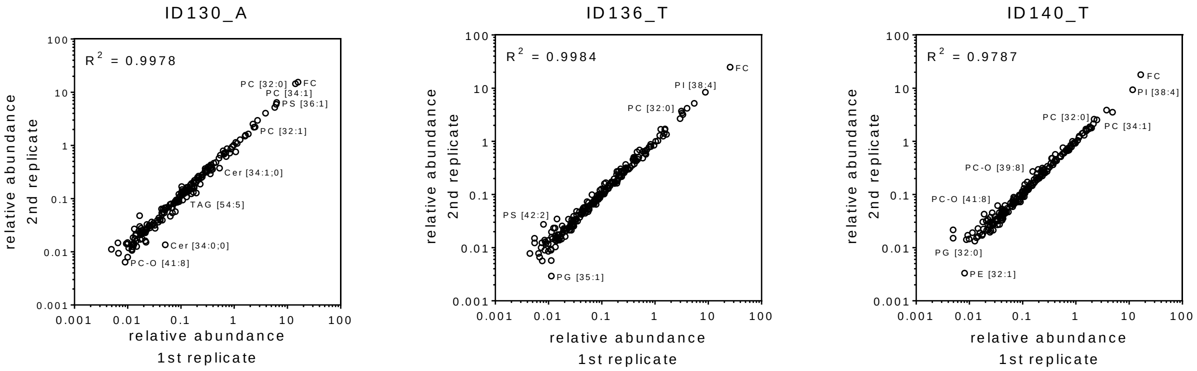


**Supplementary Figure S9.** Technical reproducibility of the lipidomics screen. Graphs show linear correlations of lipidome data from two independent technical replicates from each patient: ID130_A, ID136_T, and ID140_T. The complete extraction procedure was repeated starting at the tissue homogenates. Lipidomes of both independent extractions are compared in linear regressions. $R^2$ values are noted in the panels.

Supplementary Table S10: Internal standards used for lipid quantification.

| Abbreviation | Substance | Supplier/ID | Concentration standard mix (pmol/µL) | Amount added to sample (pmol) |
|---|---|---|---|---|
| SM-IS | 17:0 SM (d18:1/17:0) | Avanti/ 860585 | 465 | 1,860 |
| LPC-IS | 17:0 Lyso PC | Avanti/ 855676 | 654 | 2,616 |
| TAG-IS | Glyceryl triheptadecanoate | Sigma/ T2151 | 392 | 1,570 |
| PE-IS | 4ME 16:0 Diether PE | Avanti/ 999985 | 429 | 1,718 |
| PC-IS | 4ME 16:0 Diether PC | Avanti/ 999984 | 407 | 1,629 |
| Cholesterol-d7 | Cholesterol-d7 | Avanti/ 700041 | 847 | 3,387 |

Supplementary Table S11: Referencing of internal standards.

| Class | ISD positive ion mode | ISD negative ion mode | Dimension of quantitative value |
|---|---|---|---|
| PC | PC-IS | PC-IS | pmol |
| LPC | LPC-IS | LPC-IS | pmol |
| PE | PE-IS | PE-IS | pmol |
| SM | SM-IS | — | pmol |
| TAG | TAG-IS | — | pmol |
| Cer, HexCer, PG, PA, PS, PI, LPG, LPE, LPA, LPS, LPI, CL, DAG, CE | Sum of LPC-IS, SM-IS, PC-IS and TAG-IS | Sum of LPC-IS, PC-IS, and PE-IS | Normalized abundance |

Supplementary Table S12**:** Fatty acids used to draw lipid structures for LUX score calculation.

| 0 db | 1 db | 2 db | 3 db | 4, 5 and 6 db |
|---|---|---|---|---|
| 0:0 | 14:1:(9Z) | 16:2:(9Z,12Z) | 18:3:(9Z,12Z, 15Z) | 20:4:(9Z,12Z, 15Z, 18Z) |
| 14:0 | 16:1:(9Z) | 17:2:(9Z,12Z) | 20:3:(9Z,12Z, 15Z) | 22:4:(9Z,12Z, 15Z, 18Z) |
| 15:0 | 17:1:(9Z) | 18:2:(9Z,12Z) | 22:3:(9Z,12Z, 15Z) | 24:4:(9Z,12Z, 15Z, 18Z) |
| 16:0 | 18:1:(9Z) | 20:2:(9Z,12Z) | 24:3:(9Z,12Z, 15Z) | 20:5:(6Z, 9Z,12Z, 15Z, 18Z) |
| 17:0 | 19:1:(9Z) | 22:2:(9Z,12Z) | | 22:5:(6Z, 9Z,12Z, 15Z, 18Z) |
| 18:0 | 20:1:(9Z) | 24:2:(9Z,12Z) | | 22:6:(6Z, 9Z,12Z, 15Z, 18Z, 21Z) |
| 19:0 | 21:1:(9Z) | | | |
| 20:0 | 22:1:(9Z) | | | |
| 22:0 | 24:1:(9Z) | | | |
| 24:0 | | | | |

**References**


1. Schwudke, D. *et al.* Top-Down Lipidomic Screens by Multivariate Analysis of High-Resolution Survey Mass Spectra. *Analytical Chemistry* **79**, 4083-4093, DOI:10.1021/ac062455y (2007).

2. Matyash, V., Liebisch, G., Kurzchalia, T. V., Shevchenko, A. & Schwudke, D. Lipid extraction by methyl-tert-butyl ether for high-throughput lipidomics. *Journal of Lipid Research* **49**, 1137-1146, DOI:10.1194/jlr.D700041-JLR200 (2008).

3. Brown, S. H. J. *et al.* A Comparison of Patient Matched Meibum and Tear LipidomesA Tear and Meibum Sample Comparison. *Investigative Ophthalmology & Visual Science* **54**, 7417-7423, DOI:10.1167/iovs.13-12916 (2013).

4. Schuhmann, K. *et al.* Shotgun lipidomics on a LTQ Orbitrap mass spectrometer by successive switching between acquisition polarity modes. *Journal of Mass Spectrometry* **47**, 96-104, DOI:10.1002/jms.2031 (2012).
