# Supplementary material for: Lipidomes of lung cancer and tumour-free lung tissues reveal distinct molecular signatures for cancer differentiation, age, inflammation, and pulmonary emphysema
Source: Sci Rep. 2017 Sep 11;7:11087. doi: 10.1038/s41598-017-11339-1 (PMC5594029; doi:10.1038/s41598-017-11339-1)
Supplement: Supplementary file 7 — Supplement 7 [file 41598_2017_11339_MOESM7_ESM.pdf]

Supplement 7

Methods

Lipidomes of lung cancer and tumour-free lung tissues reveal distinct molecular signatures for cancer differentiation, age, inflammation, and pulmonary emphysema

Lars F. Eggers (1), Julia Müller (2), Chakravarthy Marella (1), Verena Scholz (1), Henrik Watz (3, 4), Christian Kugler (5), Klaus F. Rabe (4,5), Torsten Goldmann[#] (2,4) and Dominik Schwudke[#*] (1,4)

(1) Research Center Borstel, Bioanalytical Chemistry, Parkallee 1-40, 23845 Borstel, Germany.

(2) Pathology of the University Hospital of Lübeck and the Research Center Borstel, Clinical and Experimental Pathology, 23538 Lübeck and 23845 Borstel, Germany.

(3) Pulmonary Research Institute at LungenClinic Großhansdorf, Wöhrendamm 80, 22927 Großhansdorf, Germany.

(4) Airway Research Center North, German Center for Lung Research, Wöhrendamm 80, 22927 Großhansdorf, Germany.

(5) LungenClinic Großhansdorf, Wöhrendamm 80, 22927 Großhansdorf, Germany.

*Shared senior authorship.

[#]Corresponding author: dschwudke@fz-borstel.de

Chemicals and lipid standards

Methanol, methanol containing 0.1 % (w/v) ammonium acetate, water, and 2-propanol were purchased from Fluka (Buchs, Switzerland) in LC-MS quality. Chloroform, MTBE, acetyl chloride, ammonium chloride, glyceryl triheptadecanoate, and human haemoglobin were purchased in the highest available quality from Sigma-Aldrich (Munich, Germany). All other lipid standard substances (Supplementary Table S10) were purchased from Avanti Polar Lipids (Alabaster, AL, USA).

Lipid extraction

Directly before lipid extraction, 4 µL of the internal standard solution (Supplementary Table S10) were added to the homogenate and afterwards, 2.25 mL of methanol was added. After vortexing the mixture, 7.5 mL MTBE was added and the mixture was incubated for 30 min at room temperature with continuous shaking. Phase separation was induced by addition of 1.875 mL of water. The mixture was centrifuged for 10 min at 4,500 x *g* and the upper phase was collected in a separate tube. The lower phase was re-extracted with 2.1 mL freshly prepared upper phase obtained by mixing MTBE, methanol and water 10:3:2.5 (v/v/v). The combined organic phases were dried under nitrogen, dissolved in a mixture of chloroform, methanol and water (60/30/4.5; v/v/v), and stored at -20 °C.

Quantification of free cholesterol

Aliquots of 250 µL of the lung tissue extracts were transferred into a sample tube and dried down using a slight stream of nitrogen. Afterwards, 100 µL of a mixture of acetyl chloride and chloroform (1/5, v/v) was added to the dried samples. After rigorous mixing, samples were incubated for 60 min at room temperature. Then, remaining solvent in the reaction tubes was evaporated using a stream of dry nitrogen. The dry samples were stored at -20 °C until further use. For mass spectrometric analysis, the samples were dissolved in 500 µL 2-propanol/methanol/chloroform (4/2/1, v/v/v) containing 3.7 mM ammonium acetate. 50 µL aliquots were transferred into glass vials and were analysed by automated flow injection using a Q-TOF Ultima quadrupole time-of-flight mass spectrometer (Waters, Milford, USA) coupled to a

1100 Series HPLC system (Agilent, Waldbronn, Germany). As eluent choroform/methanol/2-propanol (1:2:4; v/v/v; containing 3.7 mM ammonium acetate) was used at a flow rate of 0.1 mL/min (0-10 min post injection) and 0.2 mL/min (10-20 min post injection). 20 µL sample were injected into the LC-MS^2 system.

Mass spectrometric acquisition consisted of (1) survey MS-scans from m/z 100 to 1200, (2) MS/MS scan of precursor ions *m/z* 446.4 (cholesterol acetyl ester) and (3) MS/MS scan of *m/z* 453.4 (cholesterol-d7 acetyl ester). Data were processed using LipidXplorer after converting the *.raw data files into *.MZxml files by MSconvert.[1] Quantification was performed using the transitions m/z 446->369 (cholesterol) in reference to the transition of m/z 453->376 for the deuterated internal standard (cholesterol-d7).

Shotgun lipidomics screen

High resolution spectra were recorded in the m/z range 300 to 1200 for the positive ion mode and m/z 300 to 1800 in the negative ion mode (R = 100,000 @ m/z 700). A spray voltage of 1.1 kV was applied and a back pressure of 1.1 psi was applied at the TriVersa Nanomate for both ion modes. The Apex Qe was used with a nebulizer gas flow of 4.0 L/min, a dry gas flow of 1.3 L/min and a dry gas temperature of 100 °C. Only mass spectrometric acquisitions were further processed, which fulfilled the following conditions: (1) a stable ESI process monitored by the total ion count (TIC); (2) all internals standards were detected and (3) a sufficient signal to noise ratios for known lipid main components was found (PC 34:1, PC 32:0, PG 36:2, PG 34:1, PI 38:4). Raw spectra were automatically processed with Data Analysis 4.0 (Bruker, Bremen, Germany). For each acquisition, high resolution Full-MS scans were averaged between 1 min and 4.5 min. Subsequently, the resulting averaged spectra were smoothed using Savitzky Golay method followed by automated peak picking. Finally, peak lists containing the centroid m/z and intensity were exported as *.csv-file (Supplement 9). For the positive ion mode at least two technical replicates were acquired for all samples except for ID17_A, ID32_T, ID32_T, ID39_A and ID6T. In the same fashion, at least two technical replicates were recorded in the negative ion mode expect for ID4_A, ID12_A and ID71_T (Supplement 9). The reproducibility of the

complete shotgun lipidomics workflow starting from the extraction procedures was demonstrated by correlation analysis of technical replicates (Supplementary Fig. S9).

Lipid identification and quantification

PC, LPC, PE, SM, and TAG species were quantified based on their respective internal standard. Peak intensities of endogenous lipids were divided by the intensity of the respective internal standard and multiplied with the amount of internal standard (Supplementary Table S10).

For Cer, hexosylceramide, PG, PA, PS, PI, LPG, LPA, LPS, LPI, and DAG *abundances* were determined in relation to the sum-intensity of added internal standards. Specifically, in positive ion mode the sum-intensity of LPC-IS, SM-IS, PC-IS, and TAG-IS was used for normalization; for the negative ion mode the sum-intensities of LPC-IS, PC-IS, and PE-IS were utilized (Supplementary Table S11). The utilized ionization mode for quantification of individual lipid species is indicated in Supplement 3. These normalized values were multiplied with the sum of the indicated internal standards to calculate abundances.

Quantification of haemoglobin in lung tissues

300 µL lung tissue homogenate (homogenates of tumour-free tissues were diluted with 300 µL 50 mM KCl buffer, tumour tissue homogenates were not diluted) were centrifuged for 1 min at 4,500 x *g*. One hundred microliters of the supernatant was analysed by an Infinite 2000 Pro plate reader (Tecan, Maennedorf, Switzerland). Absorbance spectra were measured from 320 to 700 nm with a step size of 2 nm. The absorption bands were integrated from 370 to 450 nm using SciDAVis software. The haemoglobin concentration in the samples was determined using a linear calibration with haemoglobin standard solutions.

Ammonium chloride concentration optimization

Five spray solutions (chloroform/methanol/2-propanol; 1:2:4; v/v/v) containing 0.5 mM, 0.25 mM, 0.1 mM, 0.05 mM and 0.01 mM ammonium chloride were prepared. Five lipid extracts from tumour-free human lung tissue and one blank control (water + internal standard) were diluted 1:10 with each spray solution and afterwards analysed by FT-ICR MS. The spectra were

processed as described for shotgun lipidomics. S/N instead of signal intensities were exported in *.csv files. These lists were imported into LipidXplorer. After lipid identification the results were filtered for lipid species, which were found in all samples. Percent change of responses were determined by summing up S/N of all species of a lipid class and normalizing it to the average S/N at 0.5 mM ammonium chloride. The same procedure was applied for the internal standards PC-IS, LPC-IS and PE-IS. The list of lipid species utilized for this analysis can be found in Supplement 2.

References

1. Chambers, M. C. *et al.* A cross-platform toolkit for mass spectrometry and proteomics. *Nat Biotech* **30**, 918-920, DOI:10.1038/nbt.2377 (2012).
