# Supplementary material for: Lipidomes of lung cancer and tumour-free lung tissues reveal distinct molecular signatures for cancer differentiation, age, inflammation, and pulmonary emphysema
Source: Sci Rep. 2017 Sep 11;7:11087. doi: 10.1038/s41598-017-11339-1 (PMC5594029; doi:10.1038/s41598-017-11339-1)
Supplement: Supplementary file 8 — Supplement 8 [file 41598_2017_11339_MOESM8_ESM.pdf]

Lipidomes of lung cancer and tumour-free lung tissues reveal distinct molecular signatures for cancer differentiation, age, inflammation, and pulmonary emphysema

Lars F. Eggers[1], Julia Müller[2], Chakravarthy Marella[1], Verena Scholz[1], Henrik Watz[3,4], Christian Kugler[5], Klaus F. Rabe[4,5], Torsten Goldmann[2,4,#] & Dominik Schwudke[1,4,#,*]

[1]Research Center Borstel, Bioanalytical Chemistry, Parkallee 1-40, 23845 Borstel, Germany.

[2]Pathology of the University Hospital of Lübeck and the Research Center Borstel, Location Borstel, Clinical and Experimental Pathology, 23845 Borstel, Germany.

[3]Pulmonary Research Institute at LungenClinic Großhansdorf, Wöhrendamm 80, 22927 Großhansdorf, Germany.

[4]Airway Research Center North, German Center for Lung Research, Wöhrendamm 80, 22927 Großhansdorf, Germany.

[5]LungenClinic Großhansdorf, Wöhrendamm 80, 22927 Großhansdorf, Germany.

[#]Shared senior authorship.

[*]Corresponding author: dschwudke@fz-borstel.de

Supplement 8
Scans of tissue slices

XYZ_A: Alveolar control tissue

XYZ_T: Tumour tissue

ID2_A

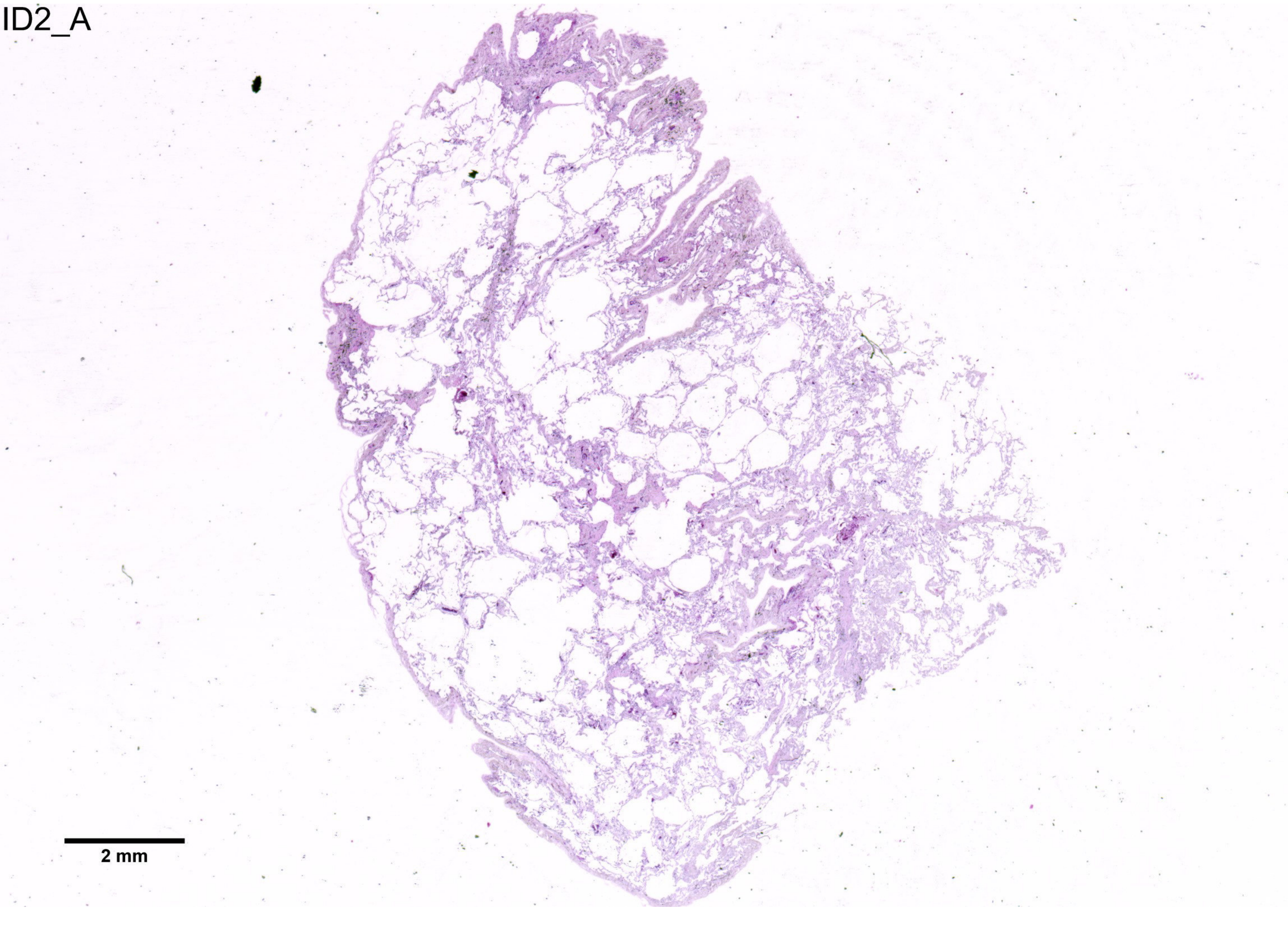

ID4_A

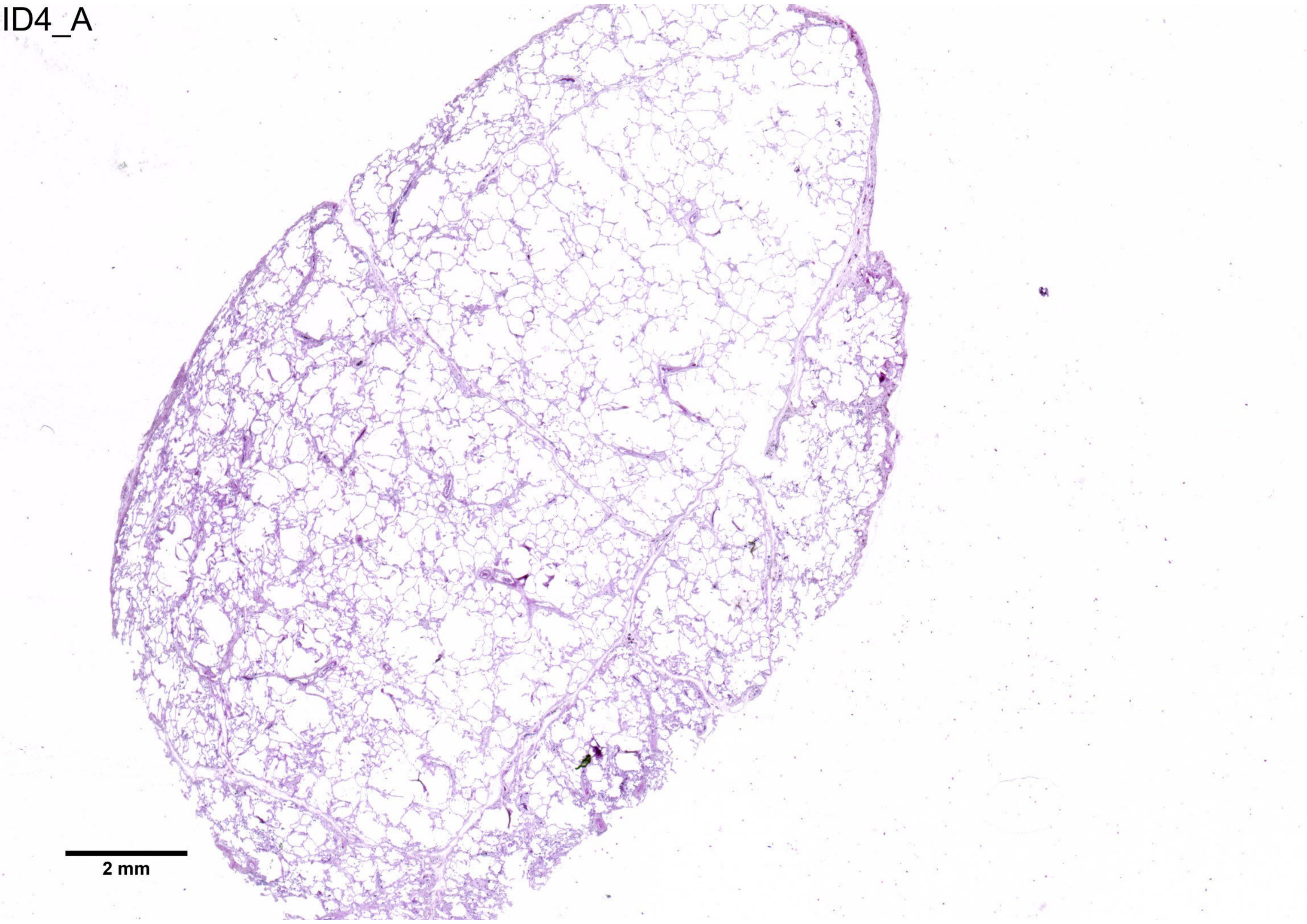

ID6_A

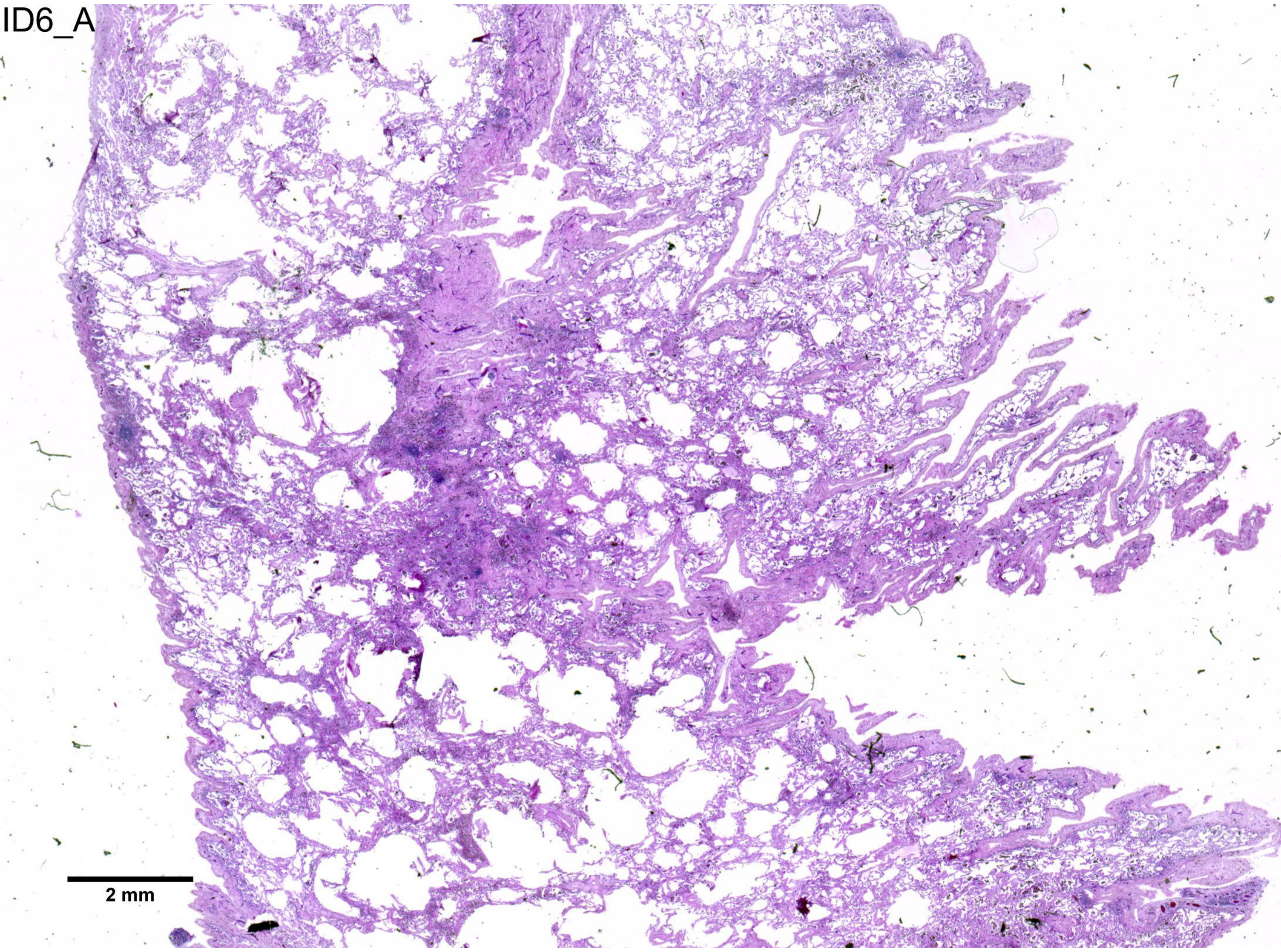

ID11_A

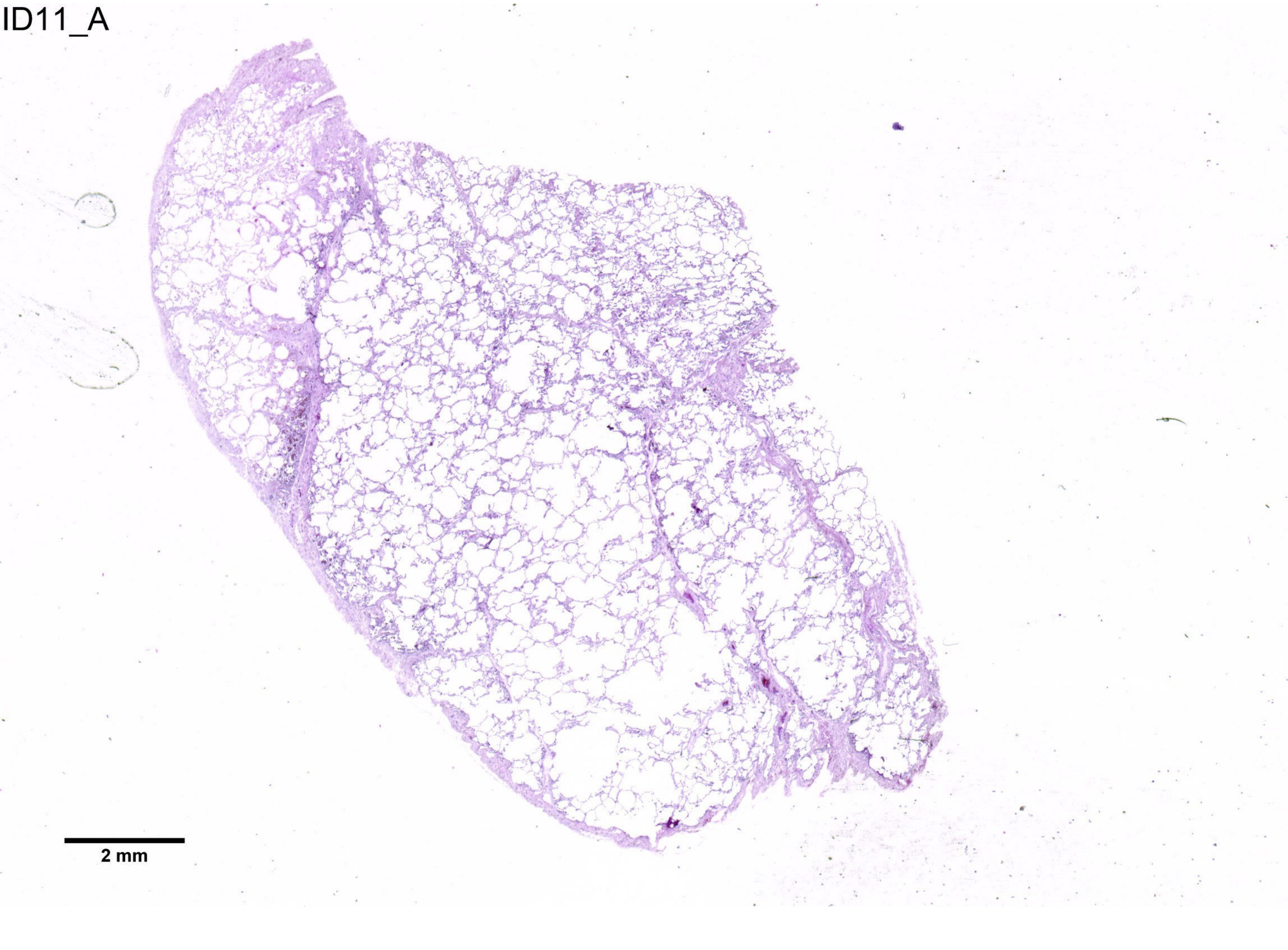

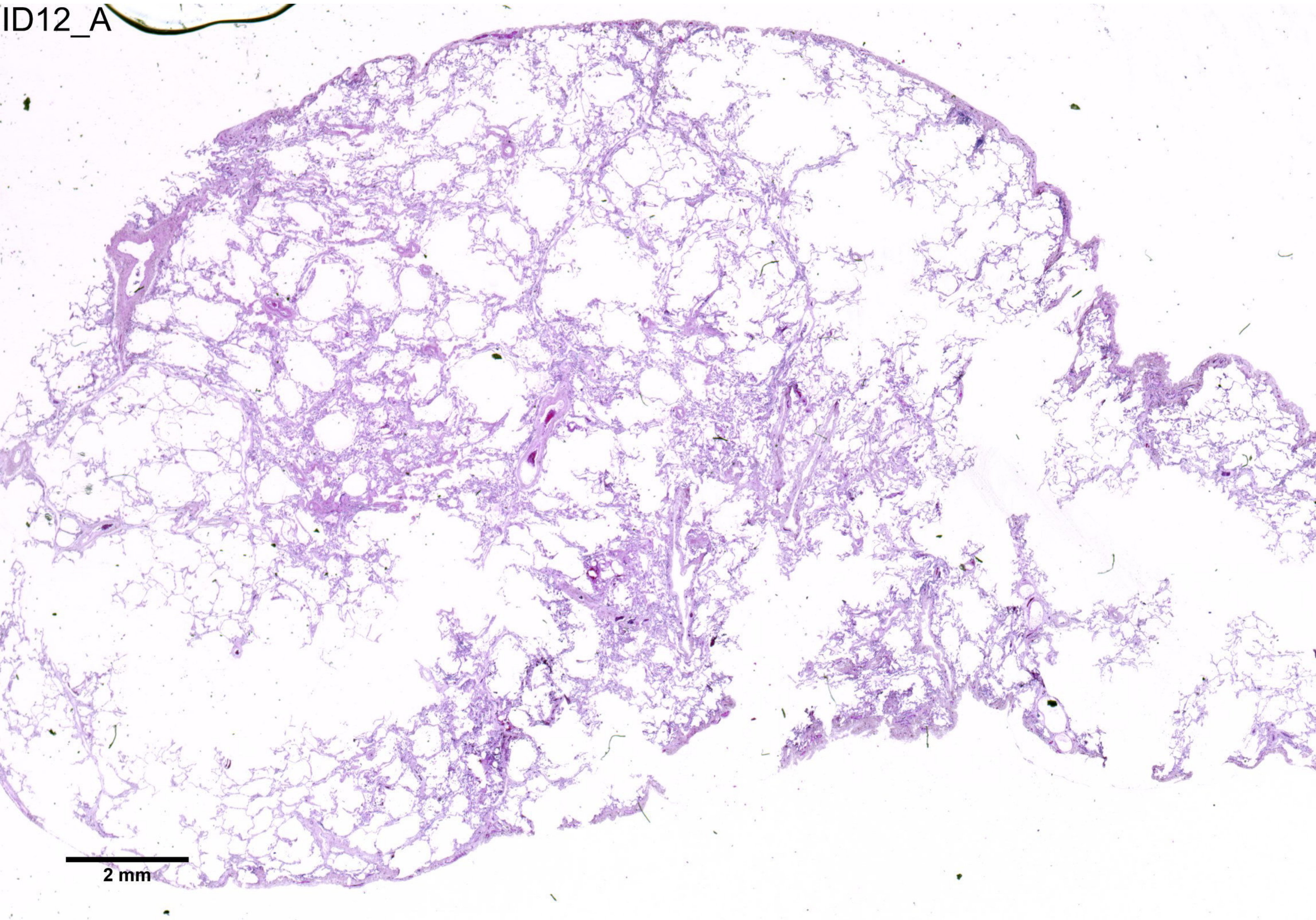
ID12_A
2 mm

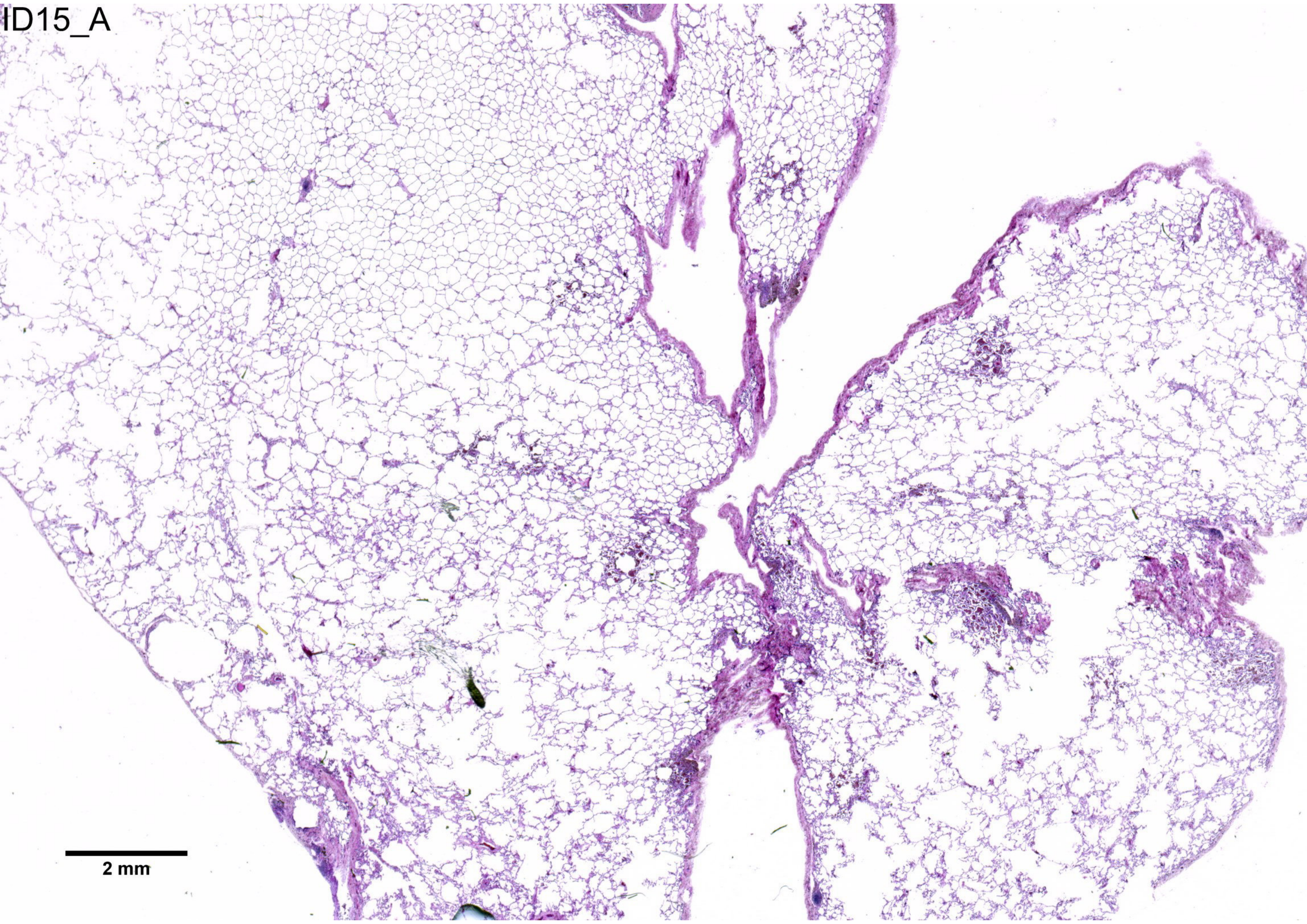
ID15_A
2 mm

ID17_A1

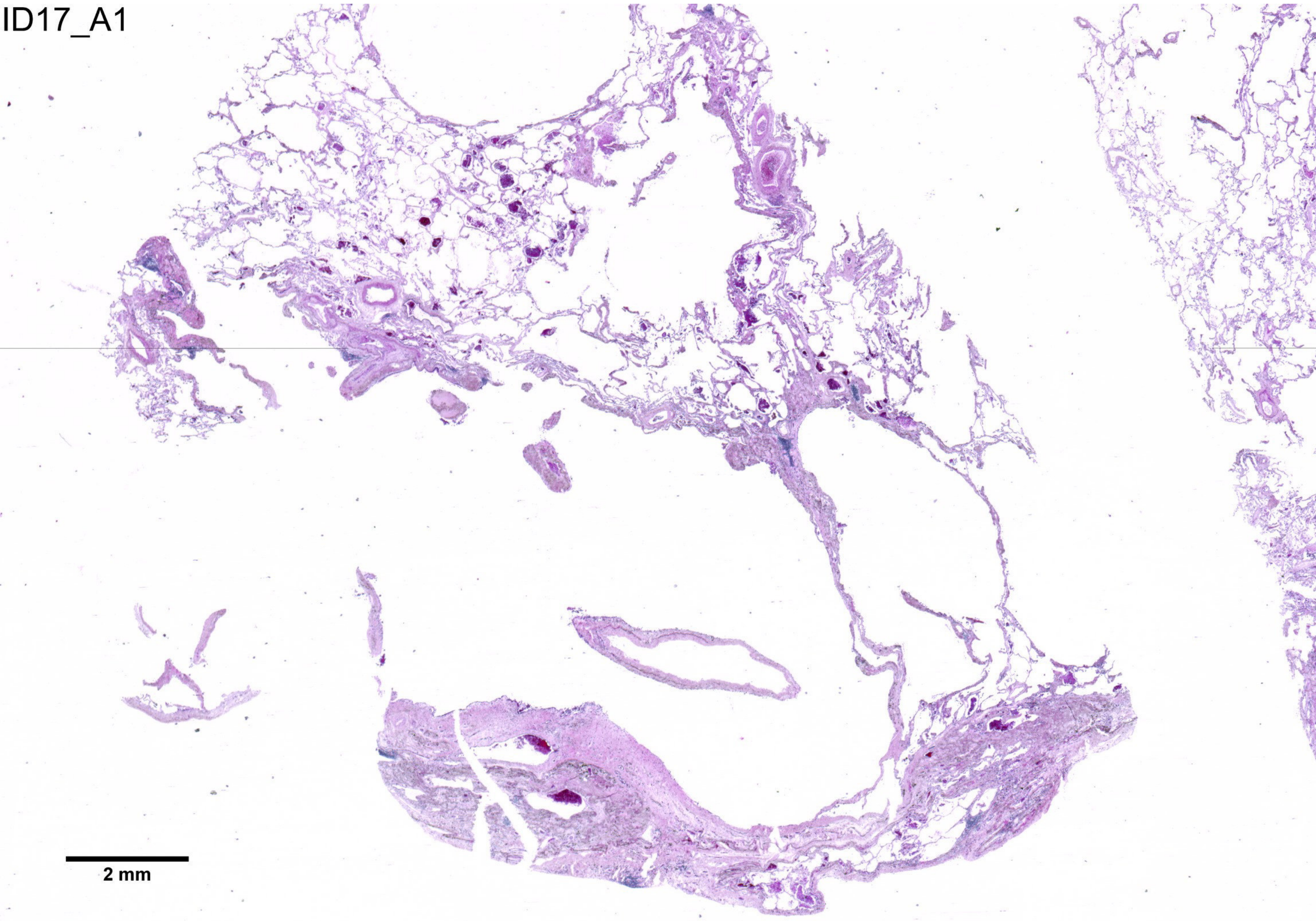

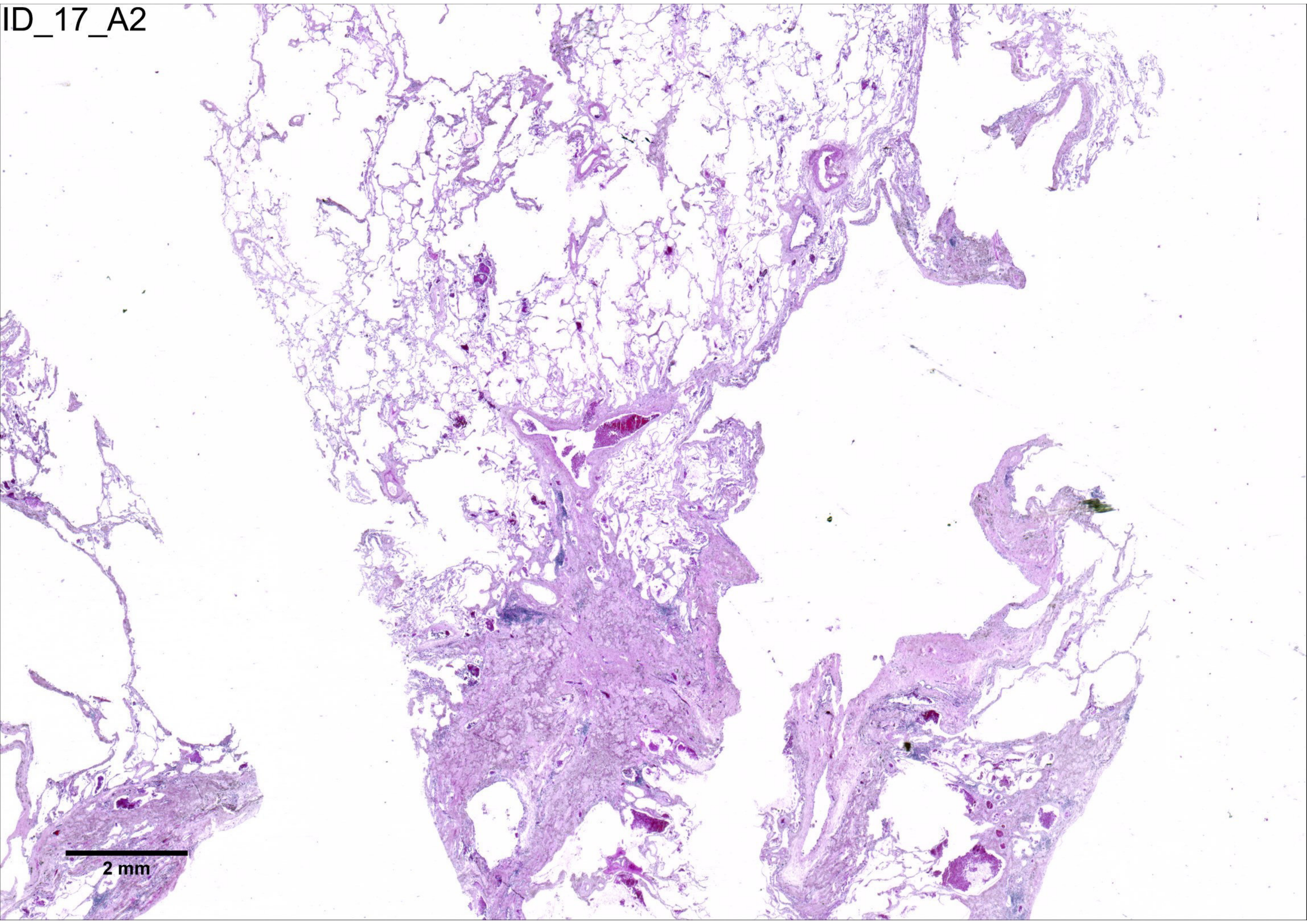
ID_17_A2
2 mm

ID18_A

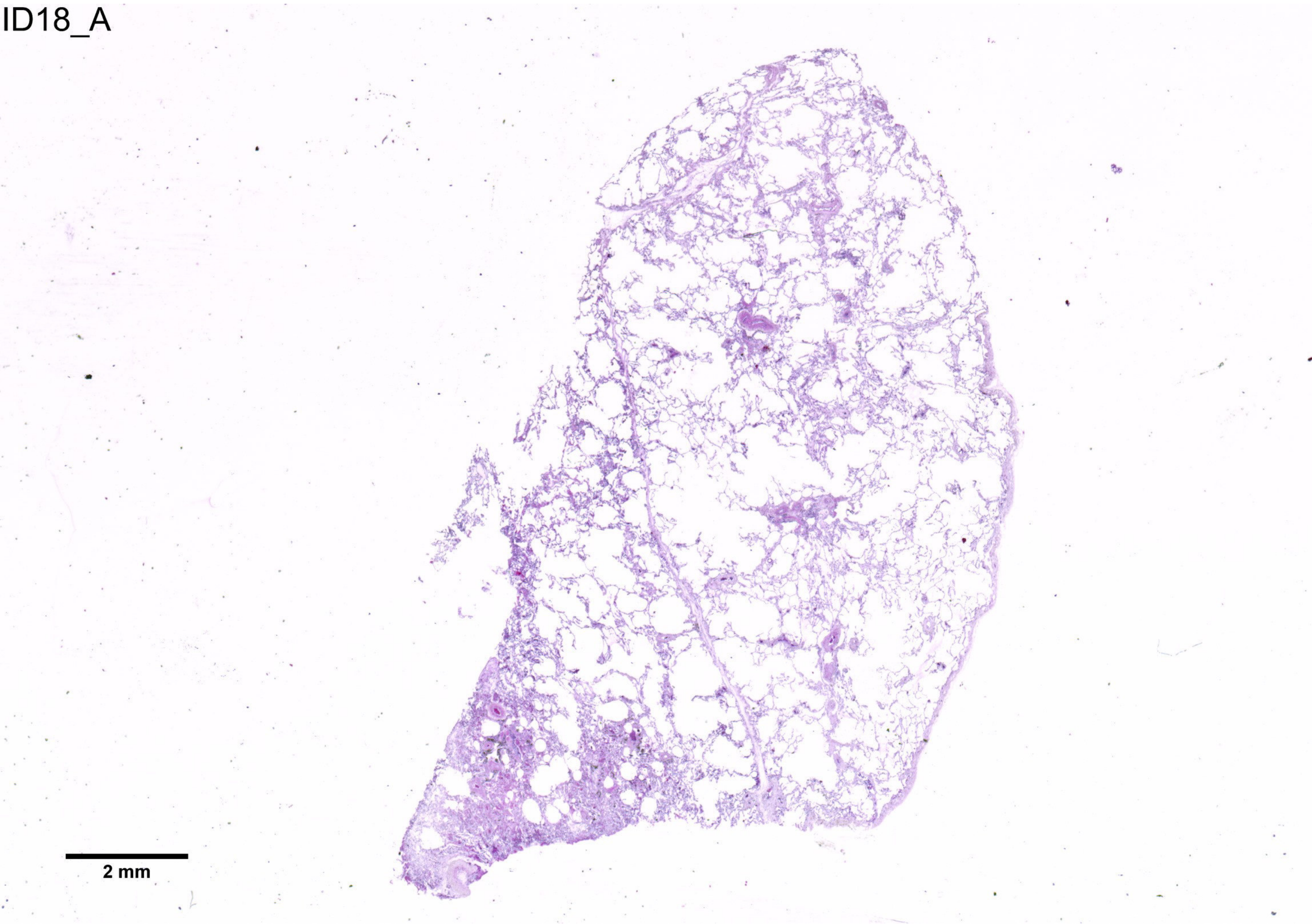

ID19_A

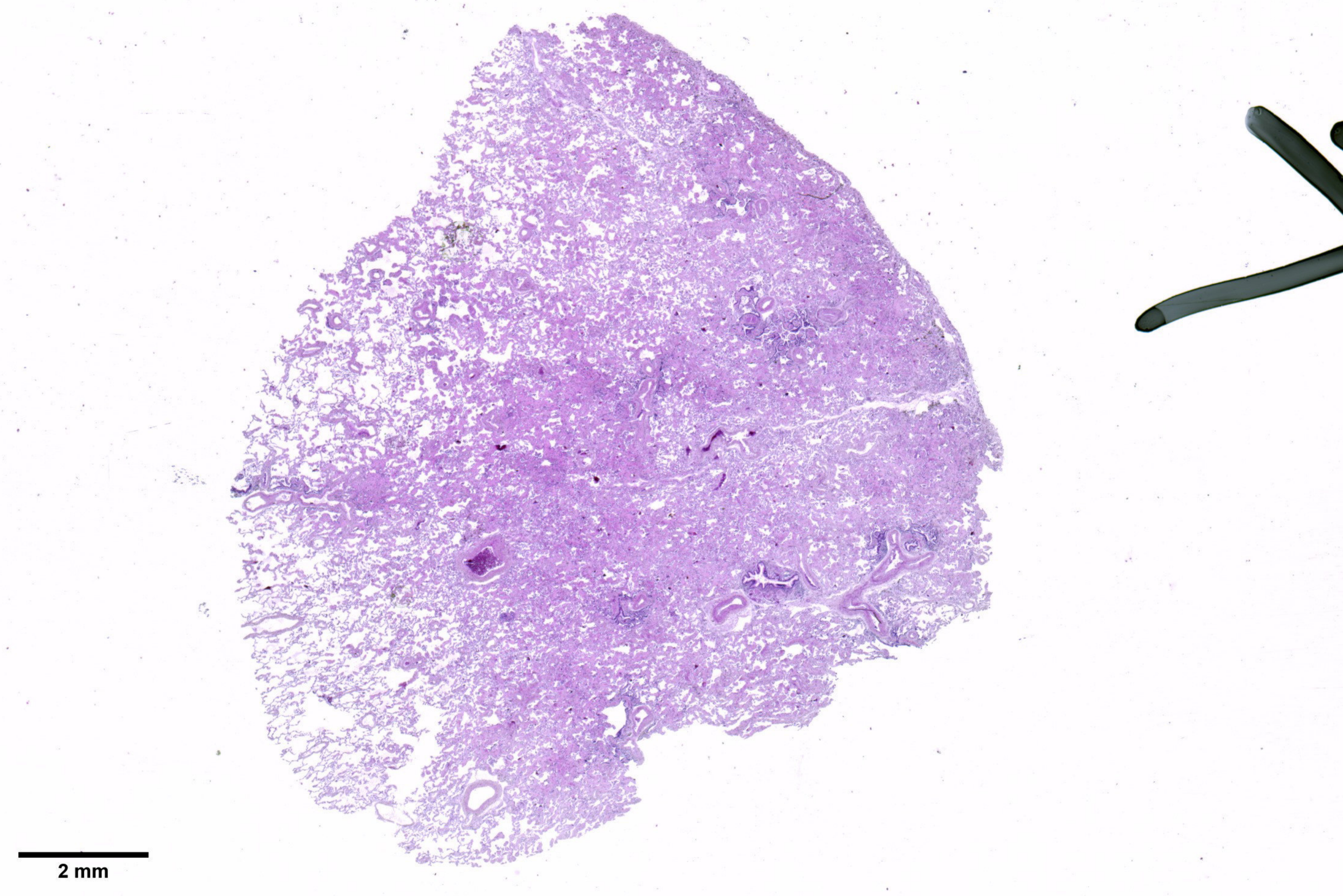

ID22_A

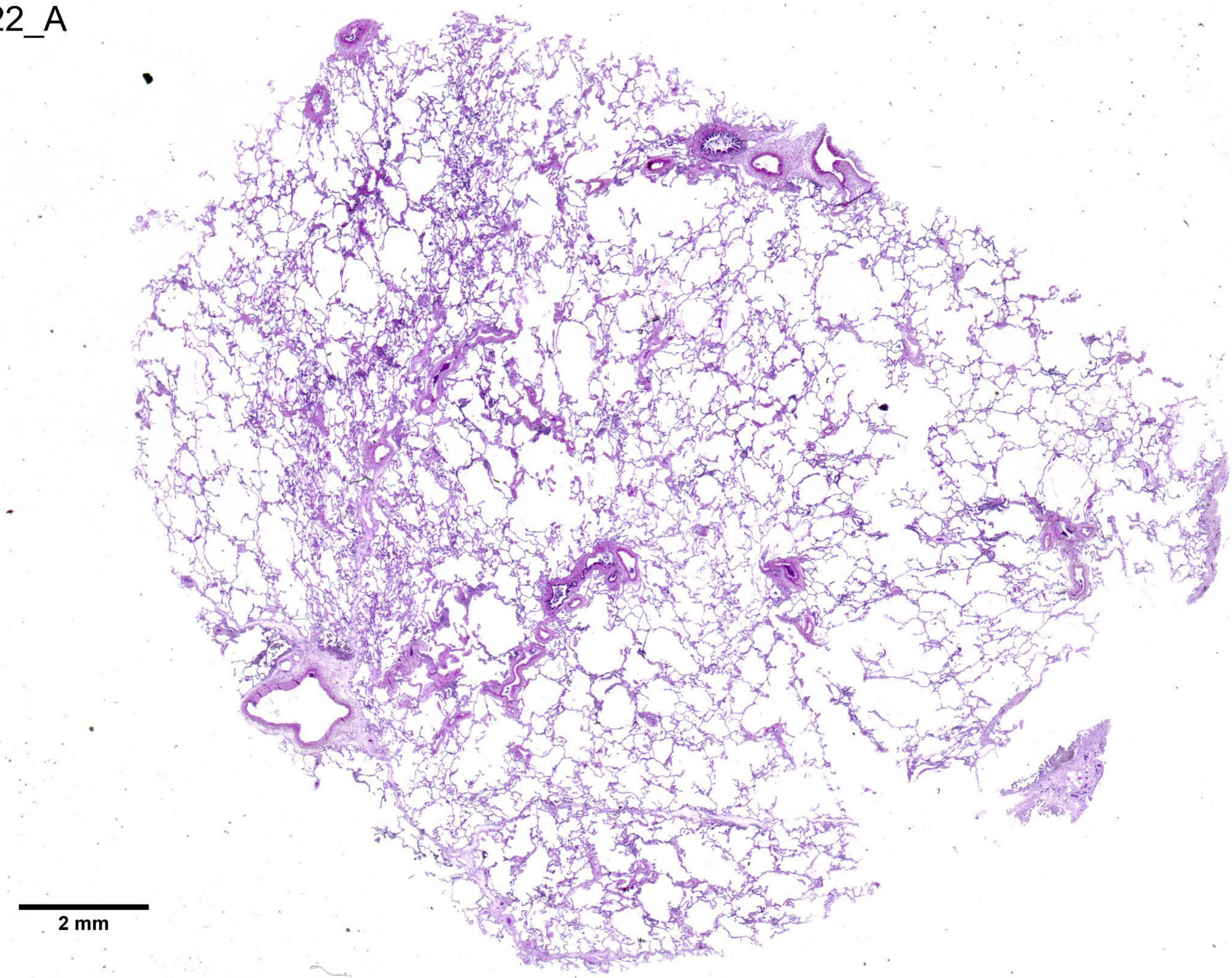

ID24_A

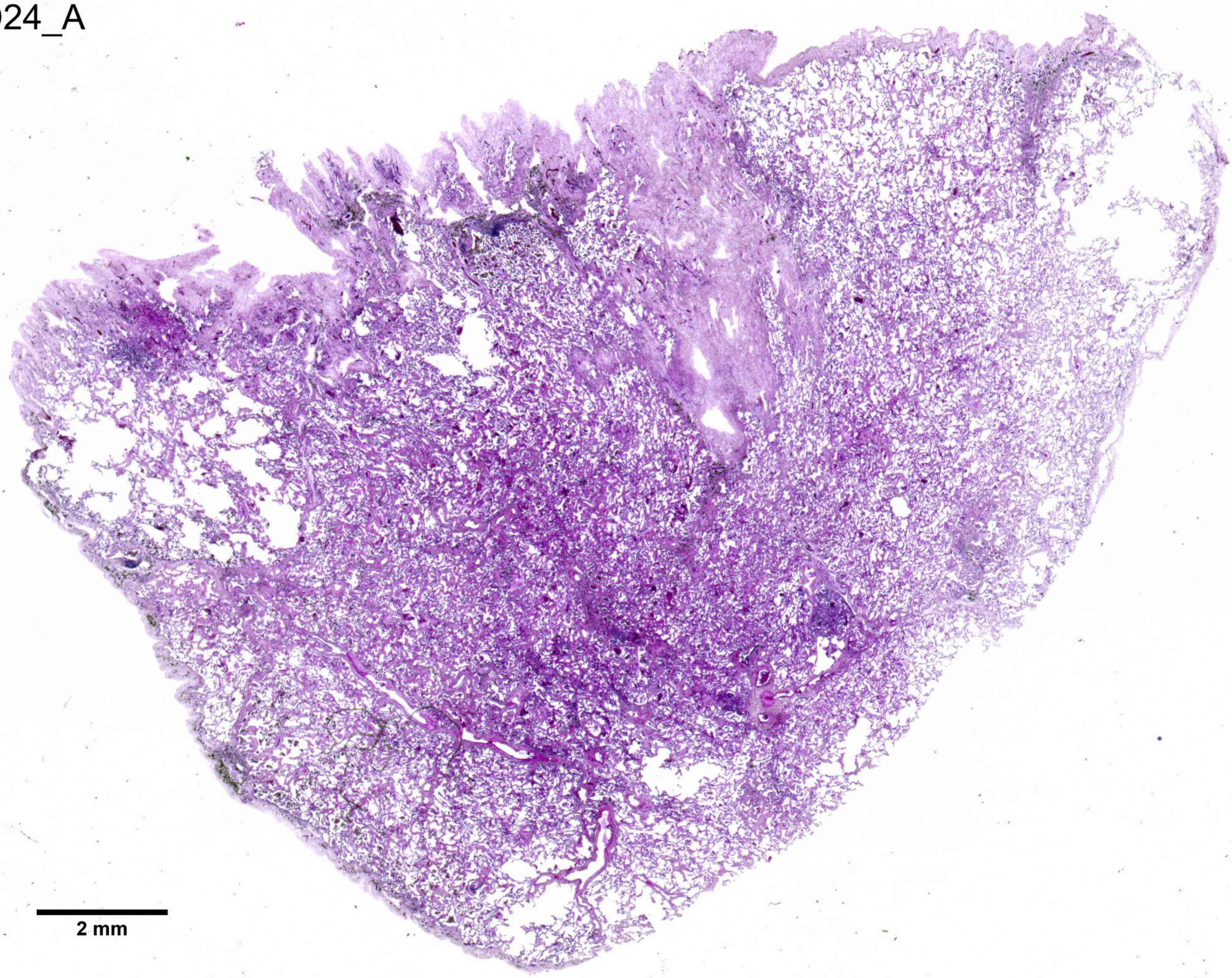

ID29_A

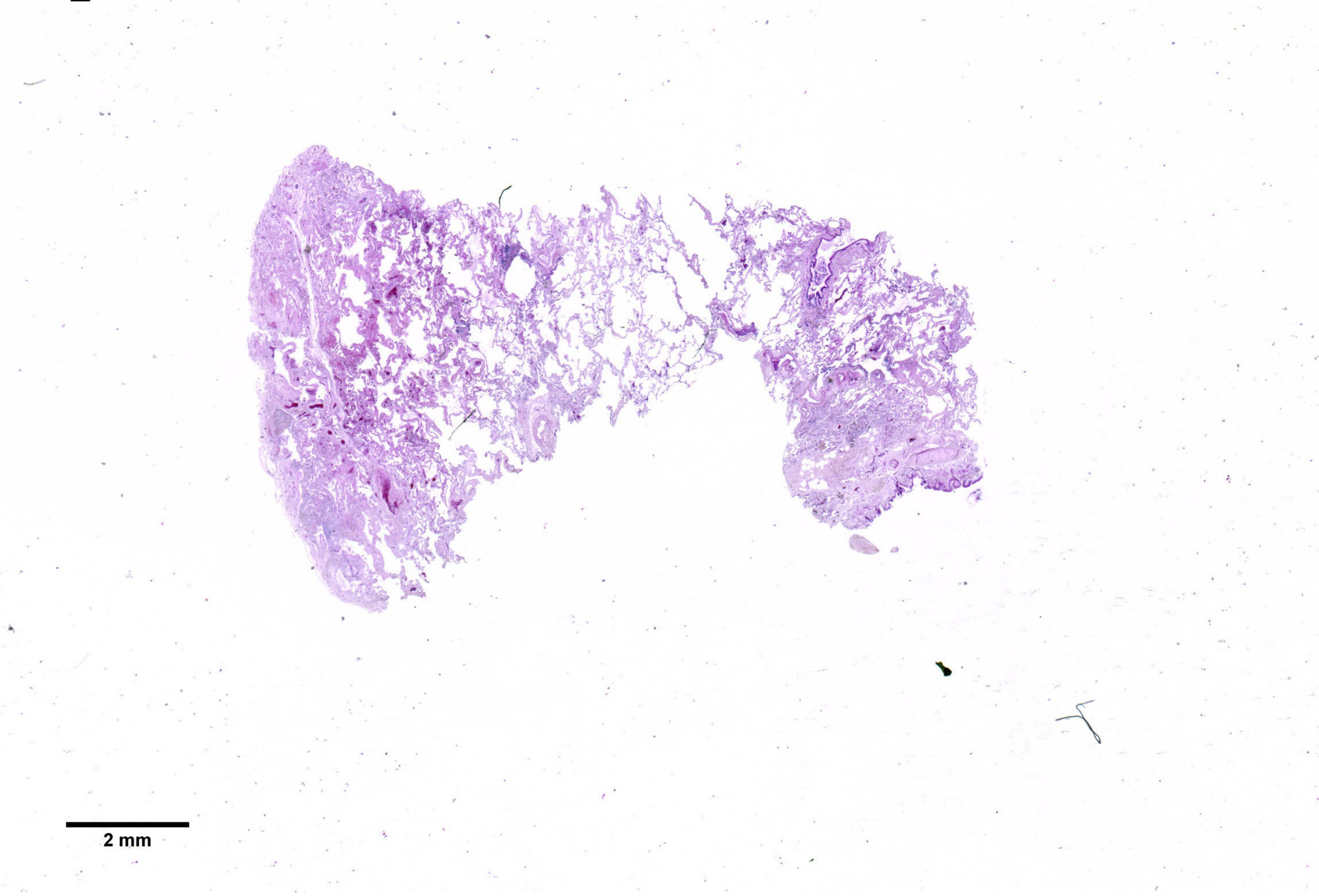

ID31_A

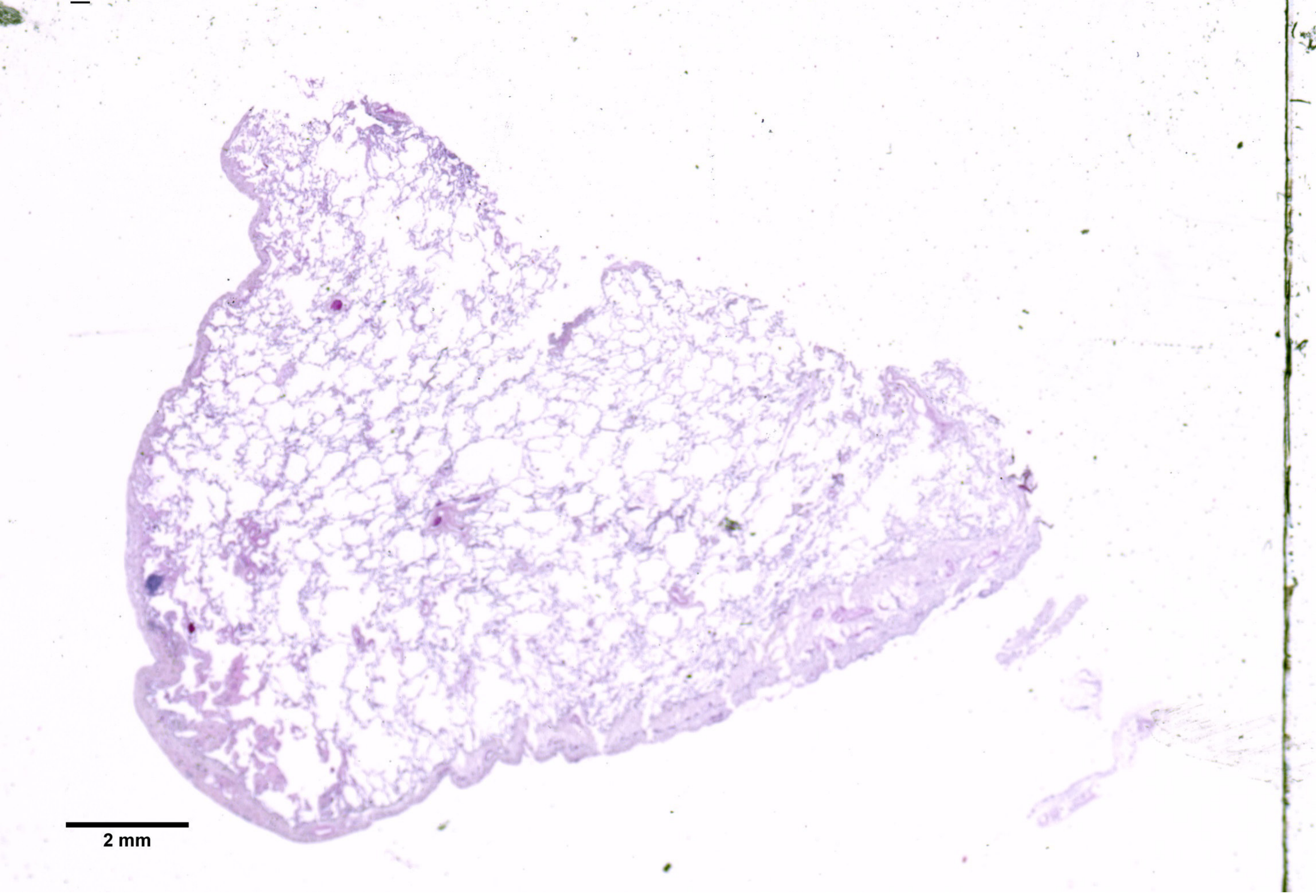

ID39_A

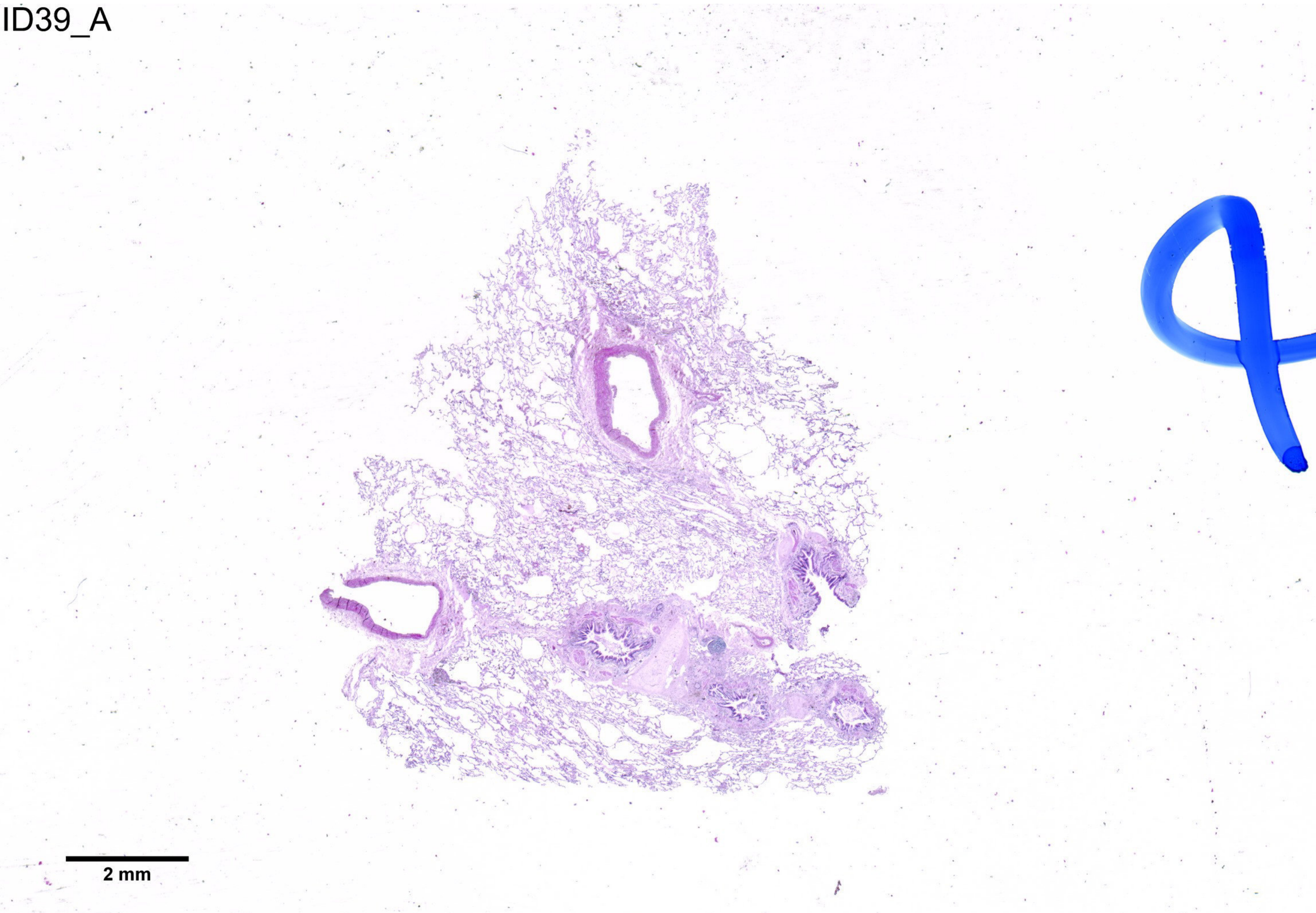

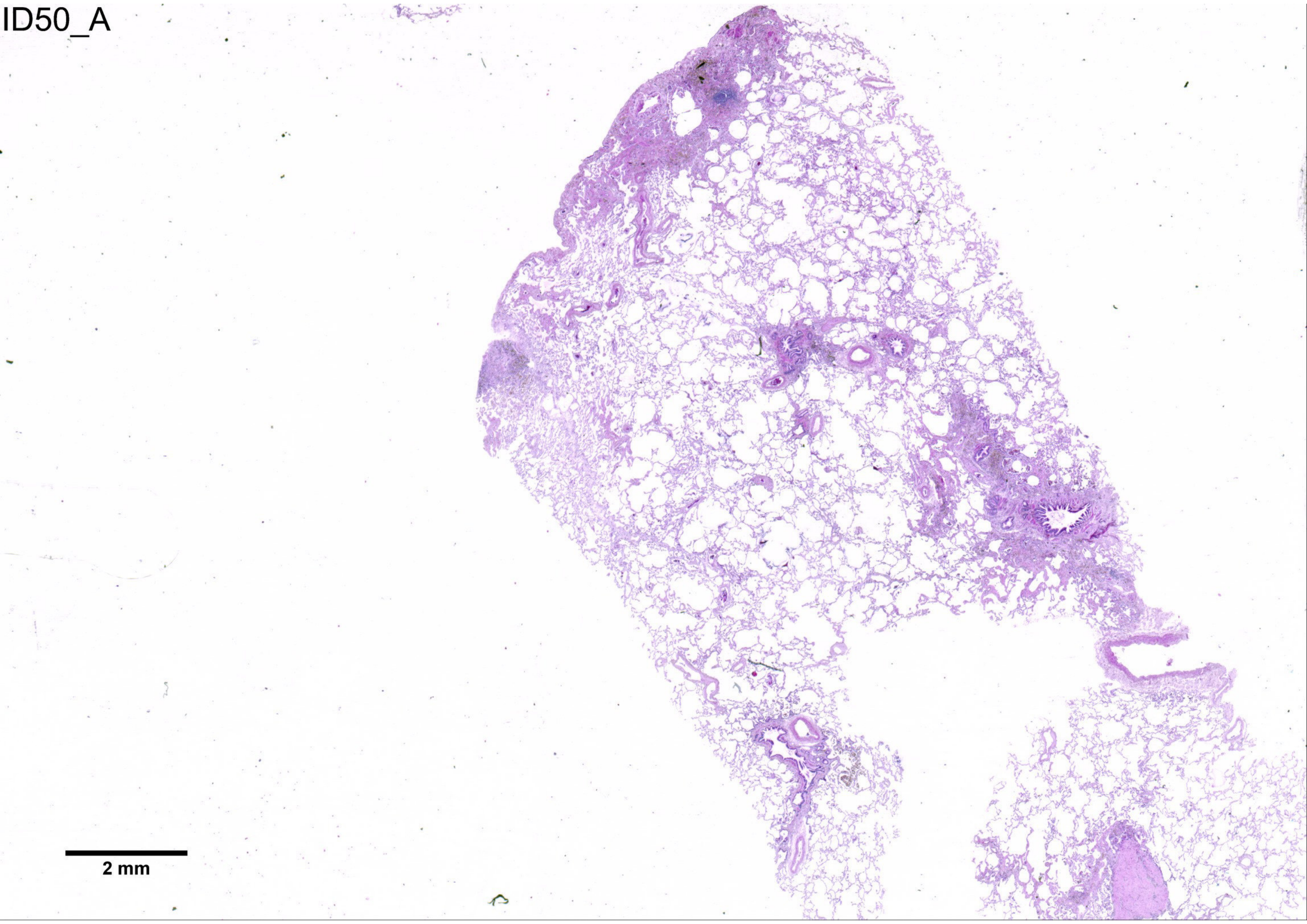
ID50_A
2 mm

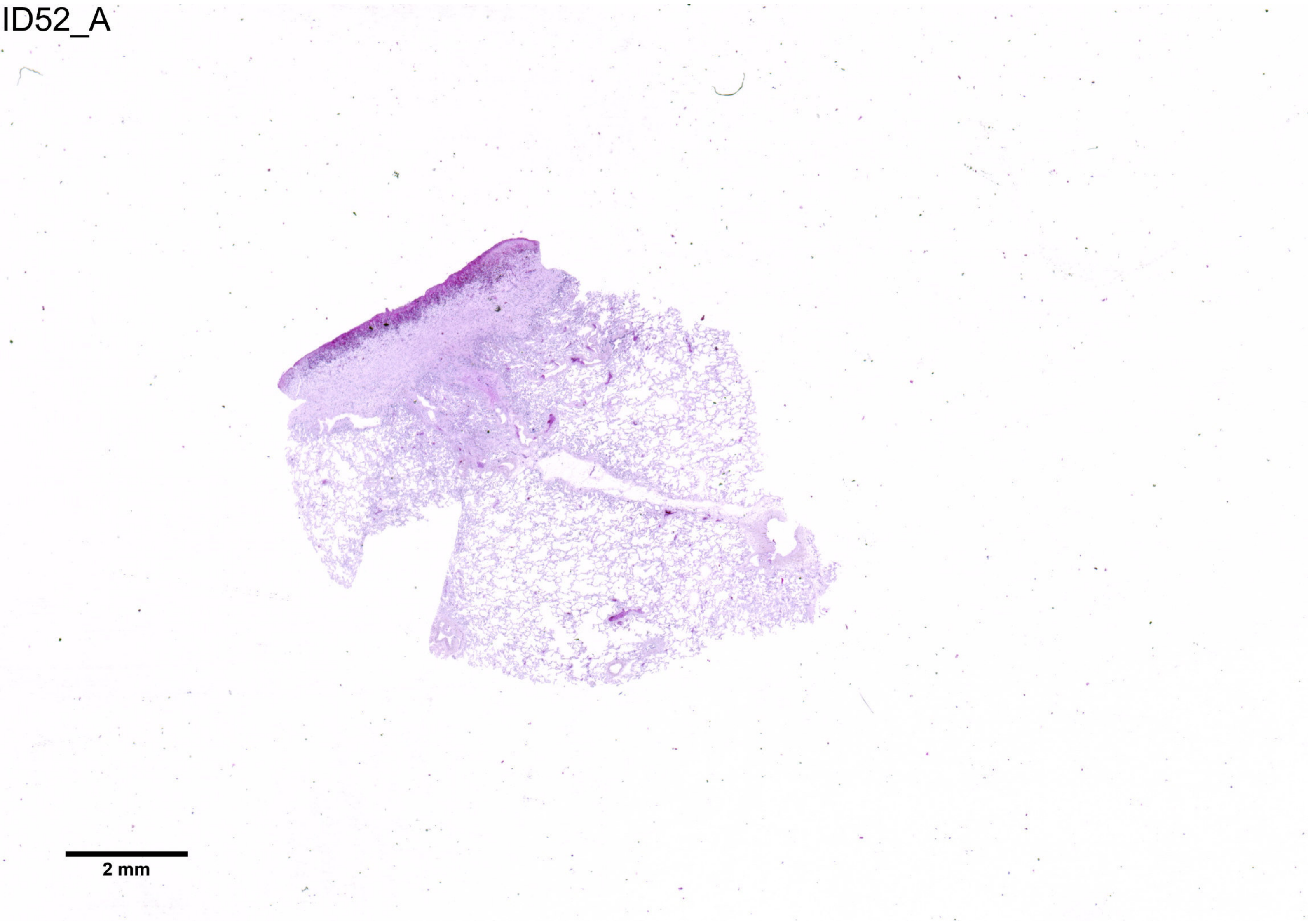
ID52_A
2 mm

ID53_A

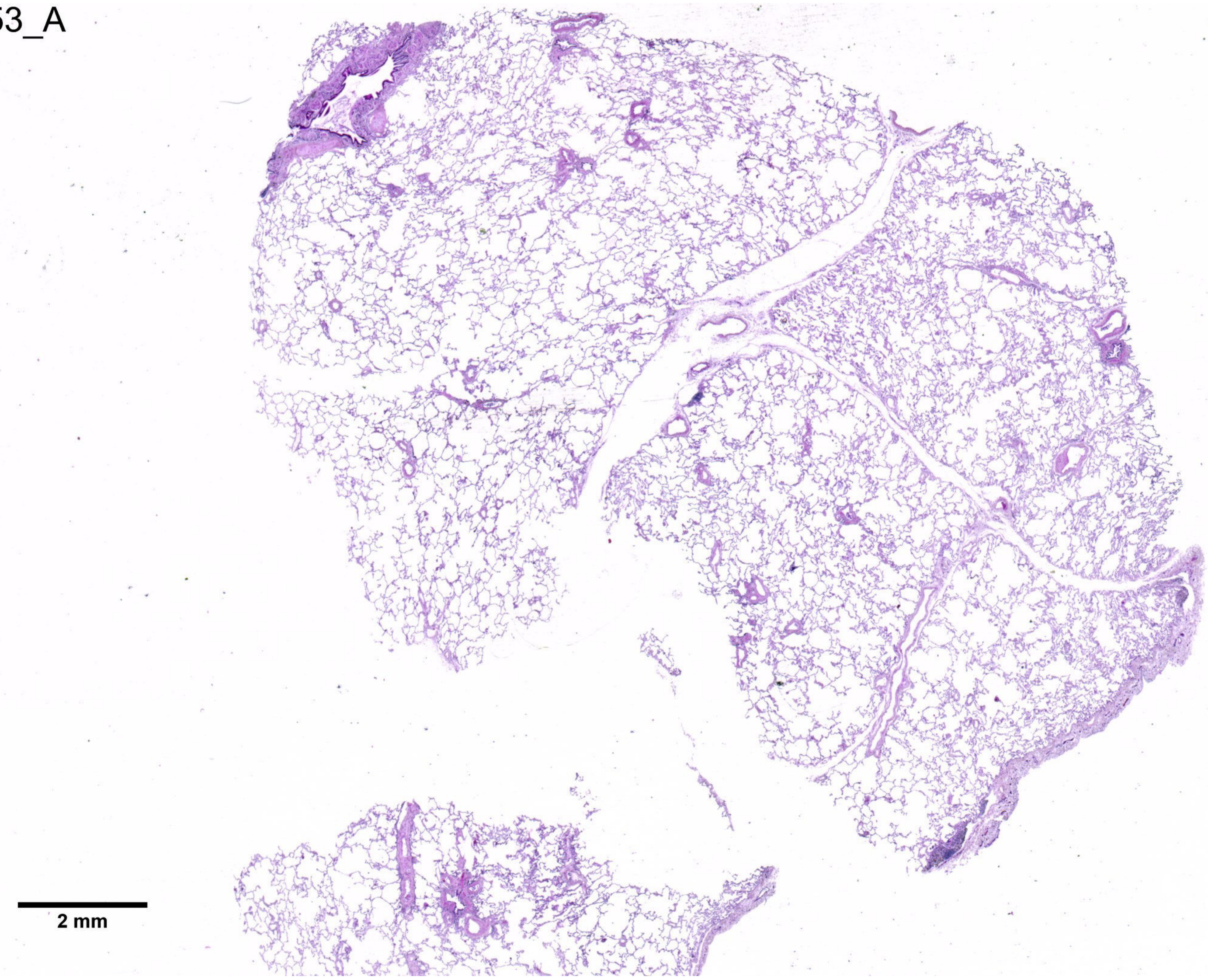

ID61_A

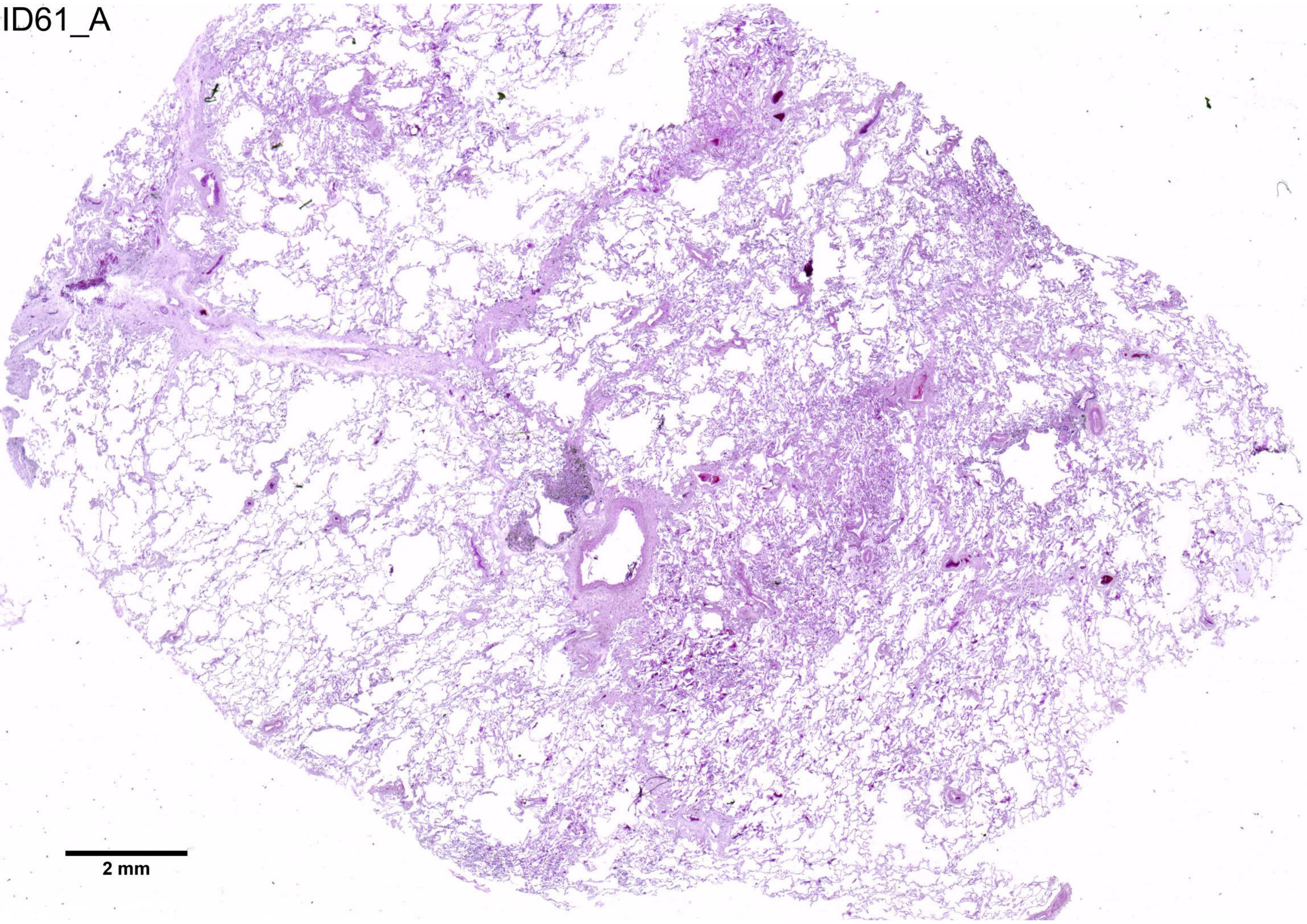

ID64_A

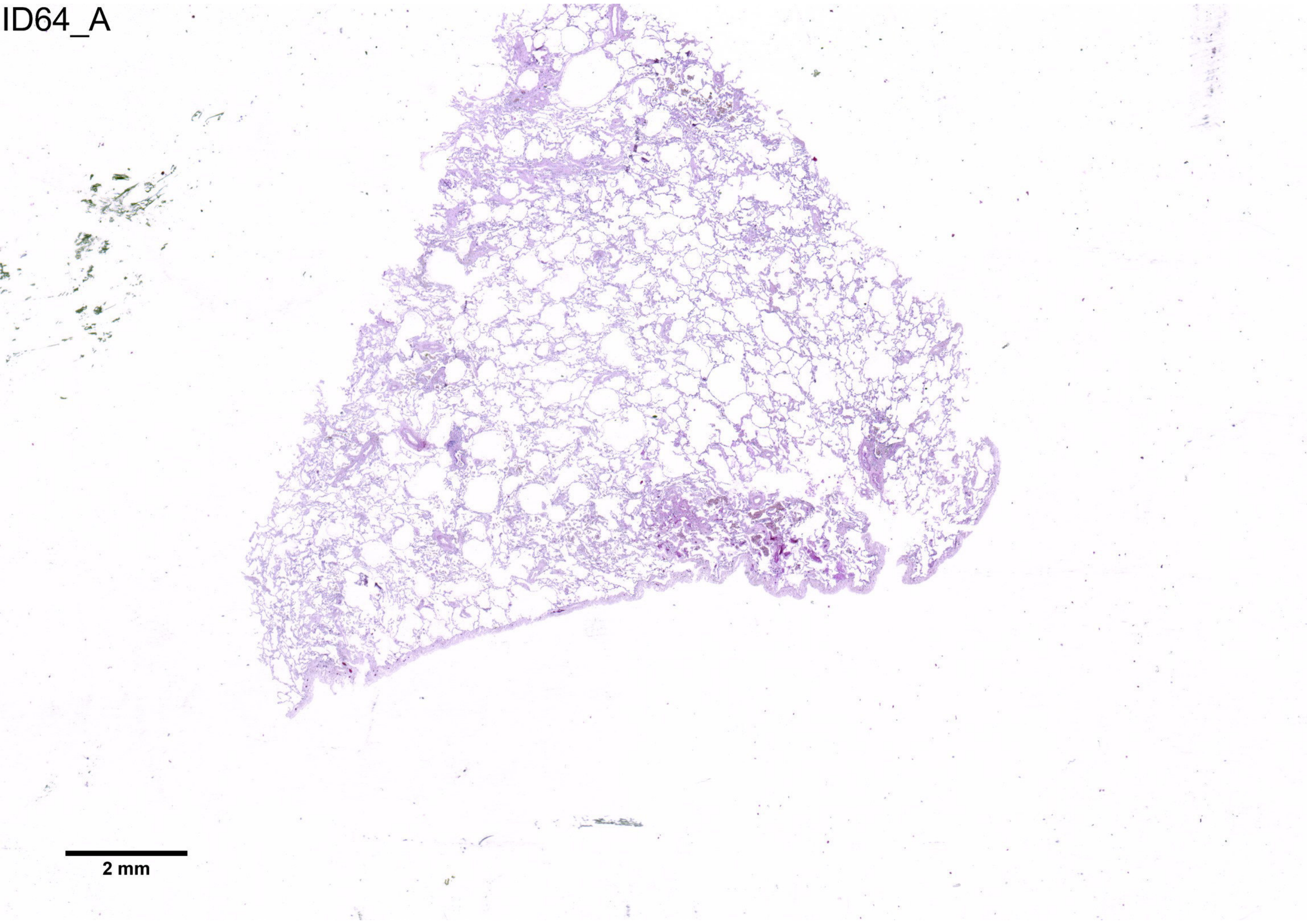

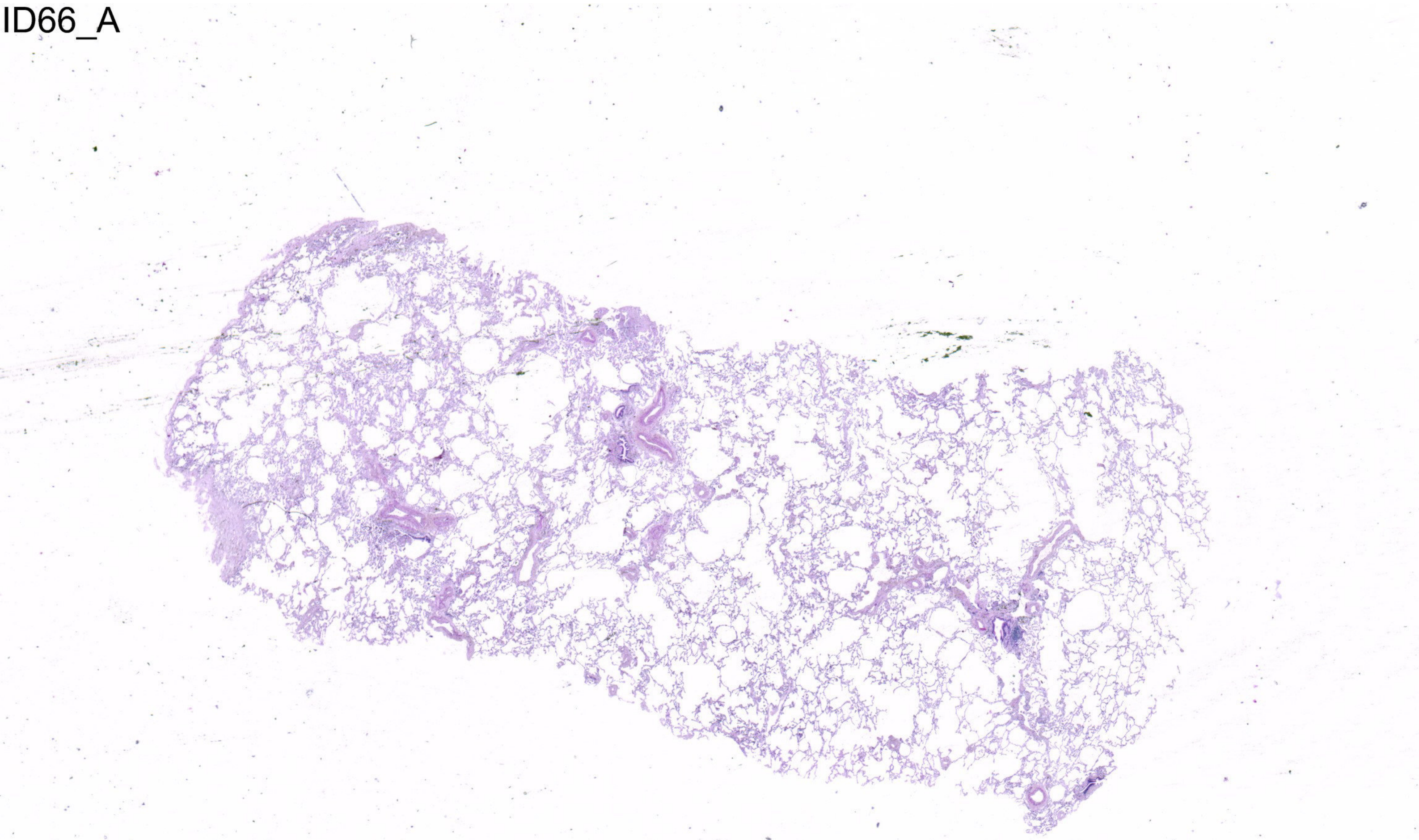
ID66_A
2 mm

ID67_A

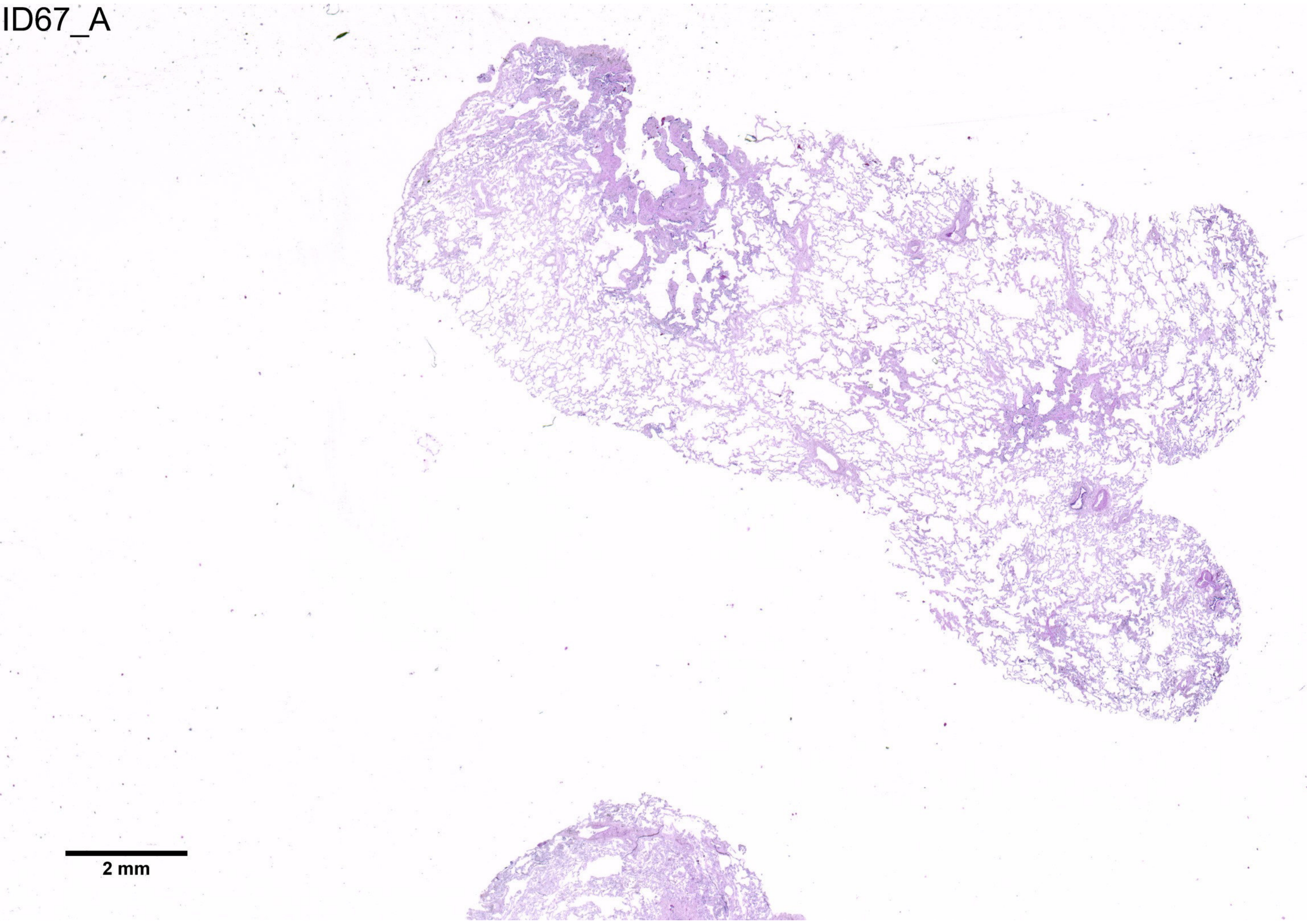

ID72_T

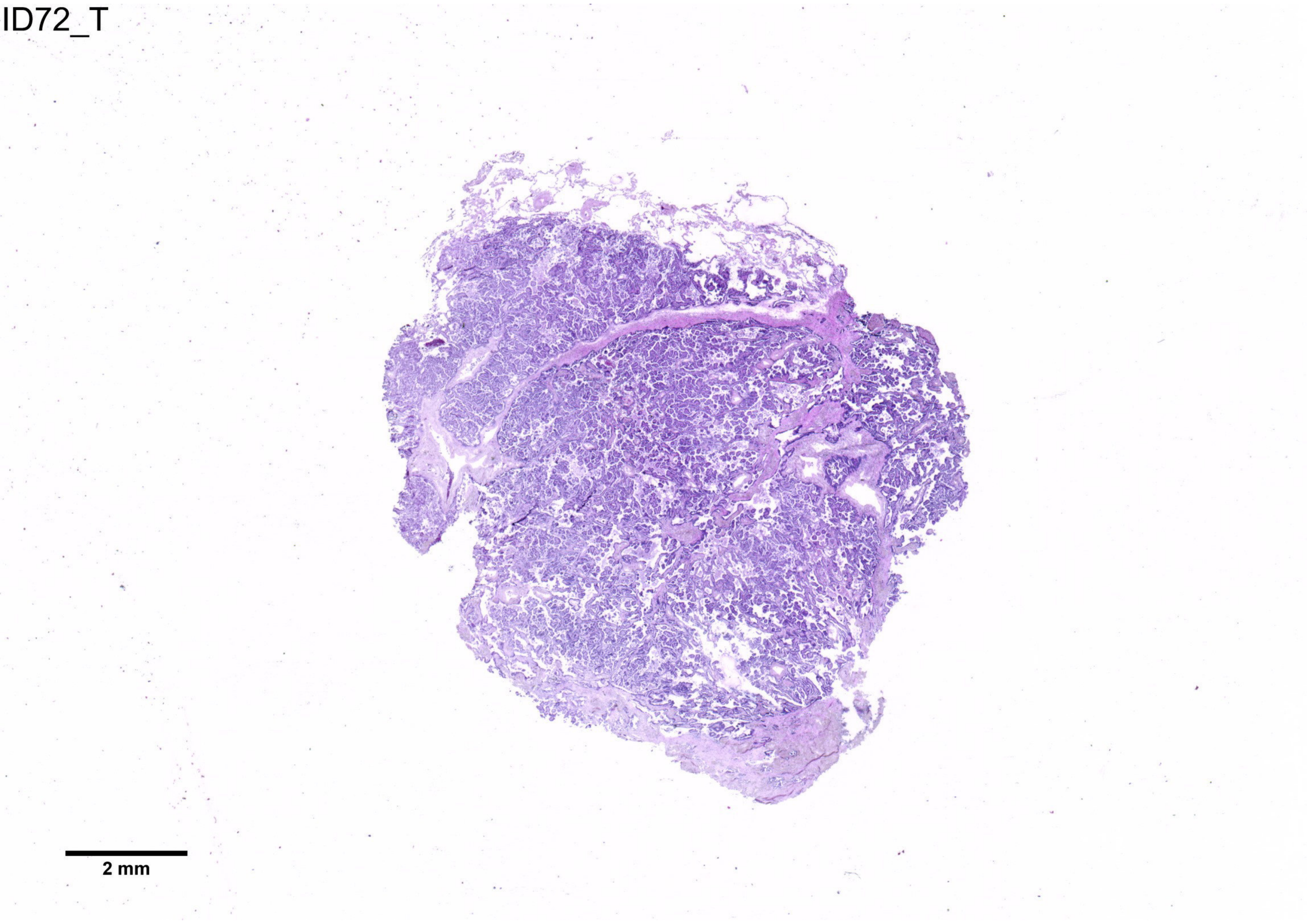

ID2_T

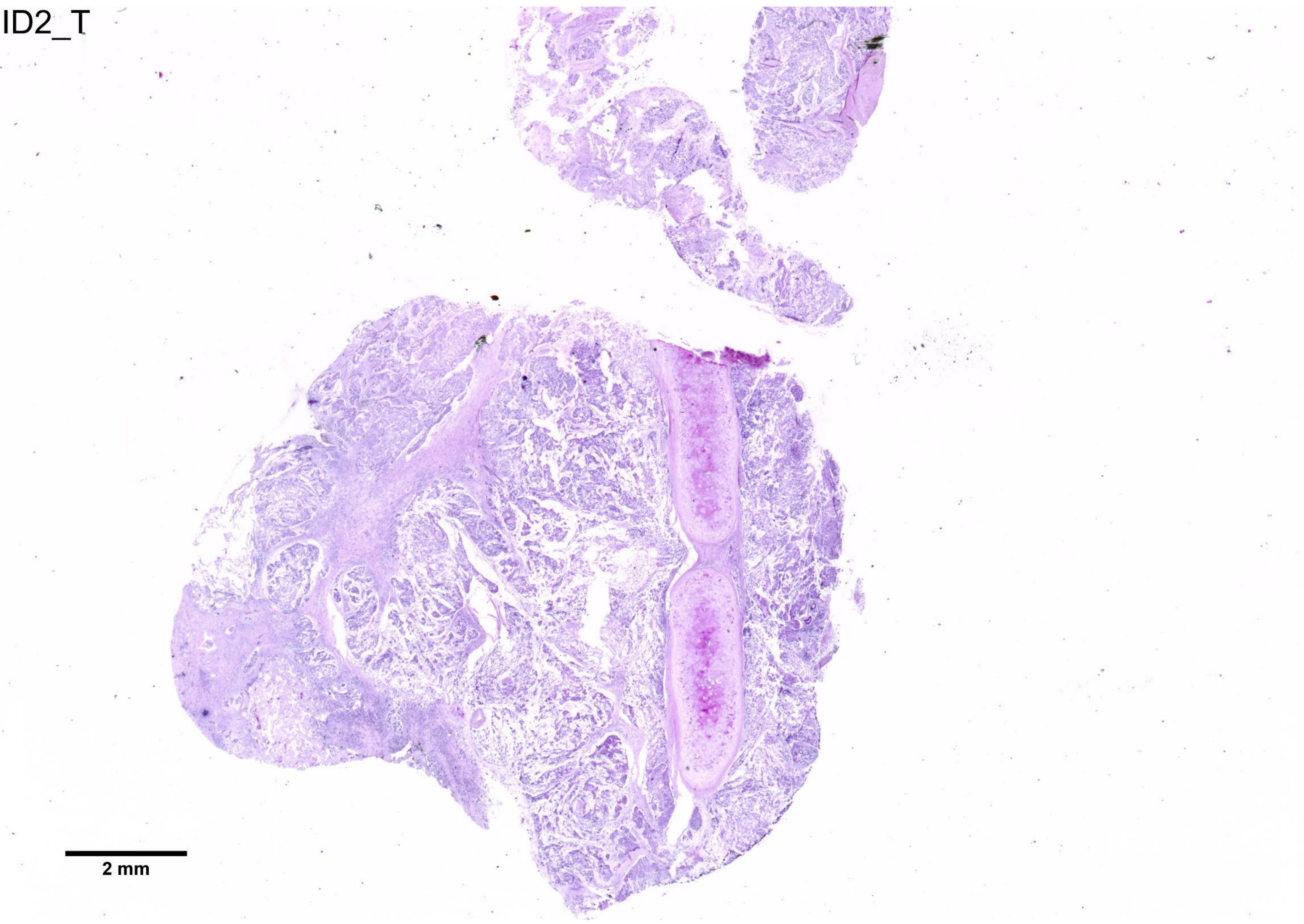

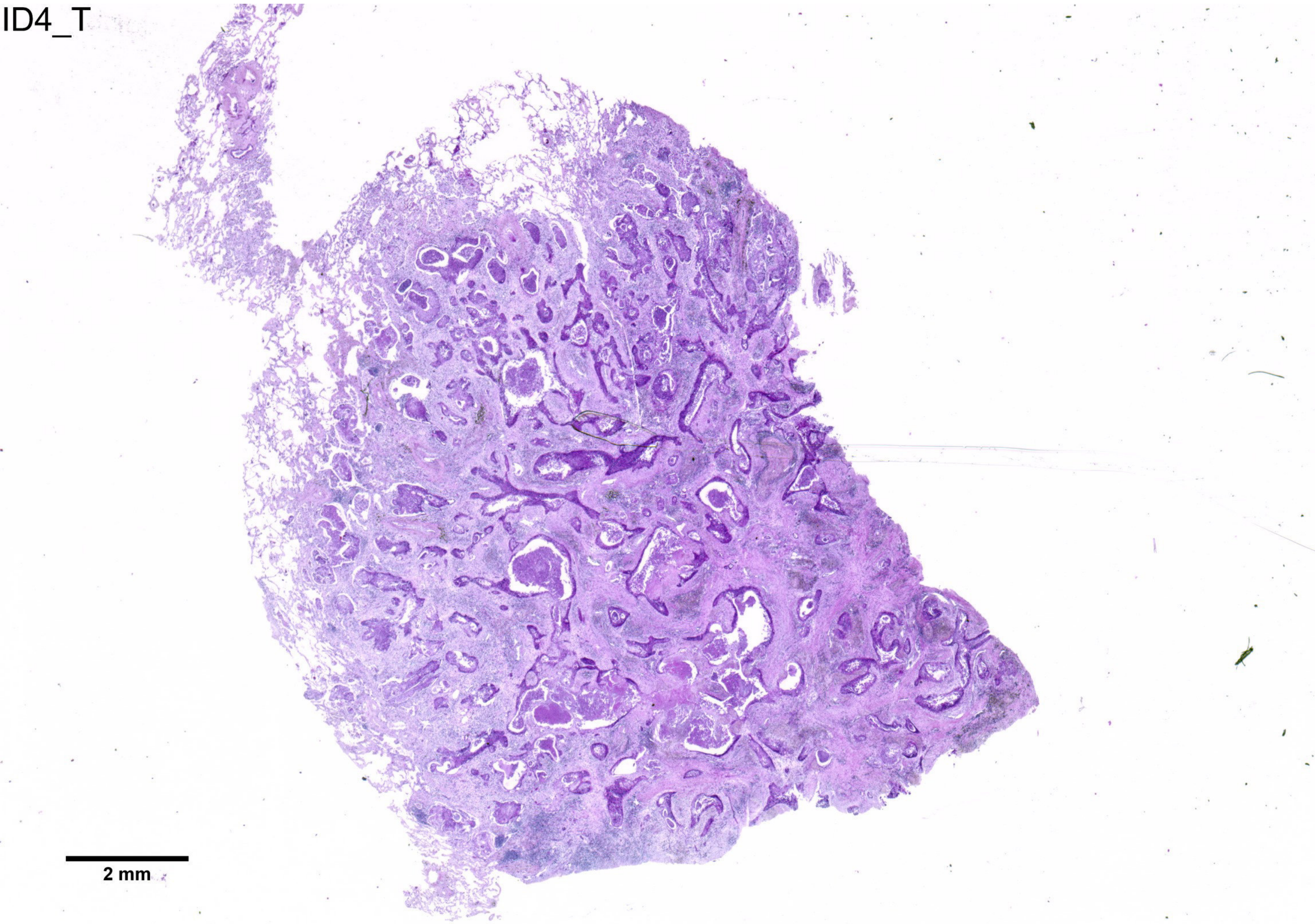

ID4_T
2 mm

ID6_T

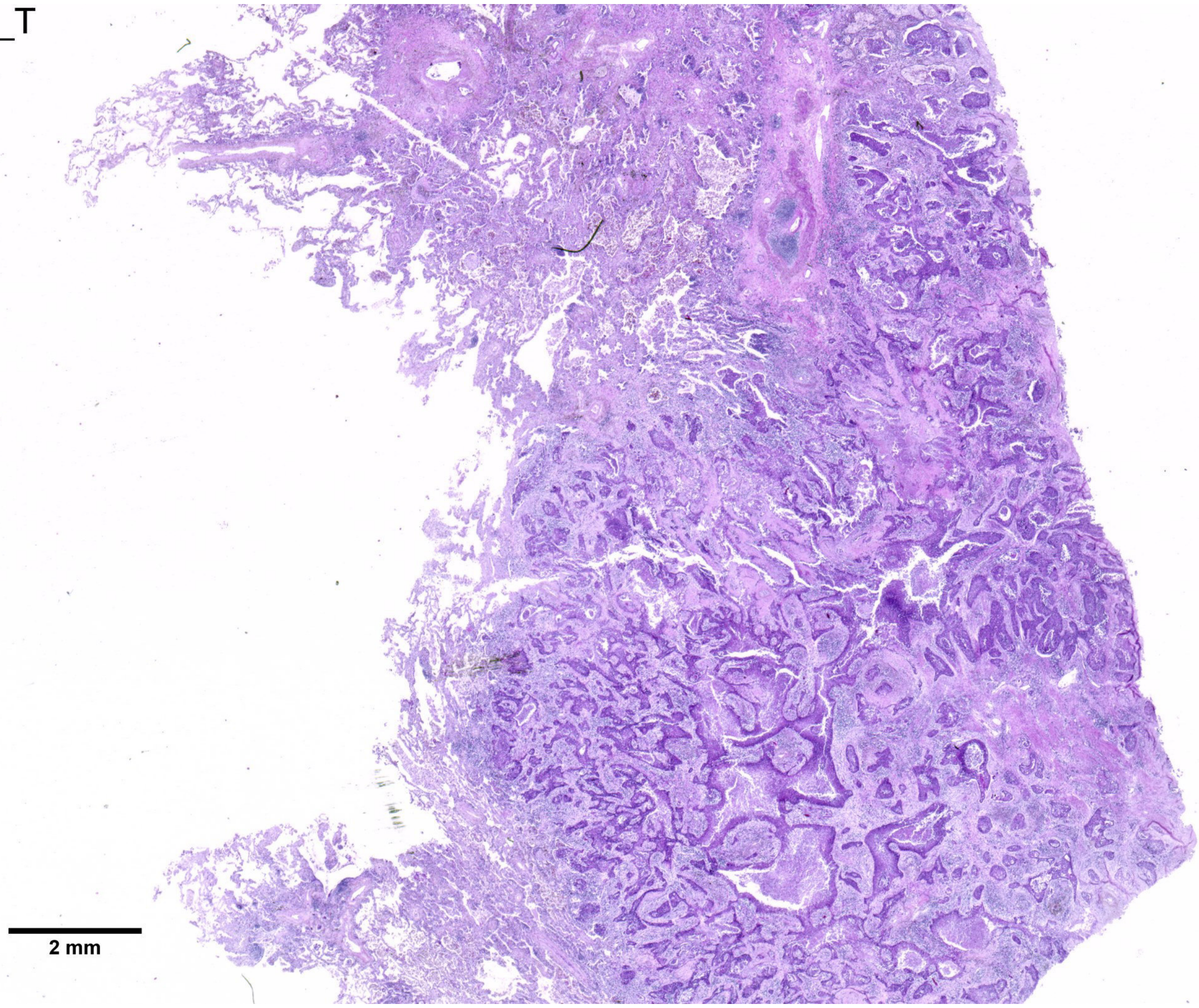

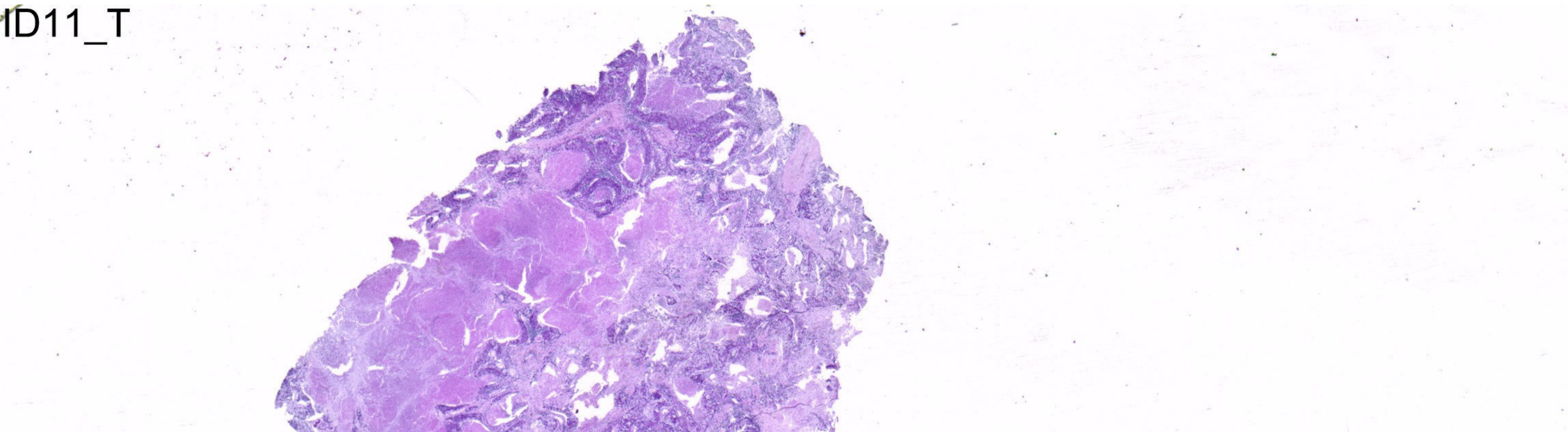
ID11_T
2 mm

ID12_T

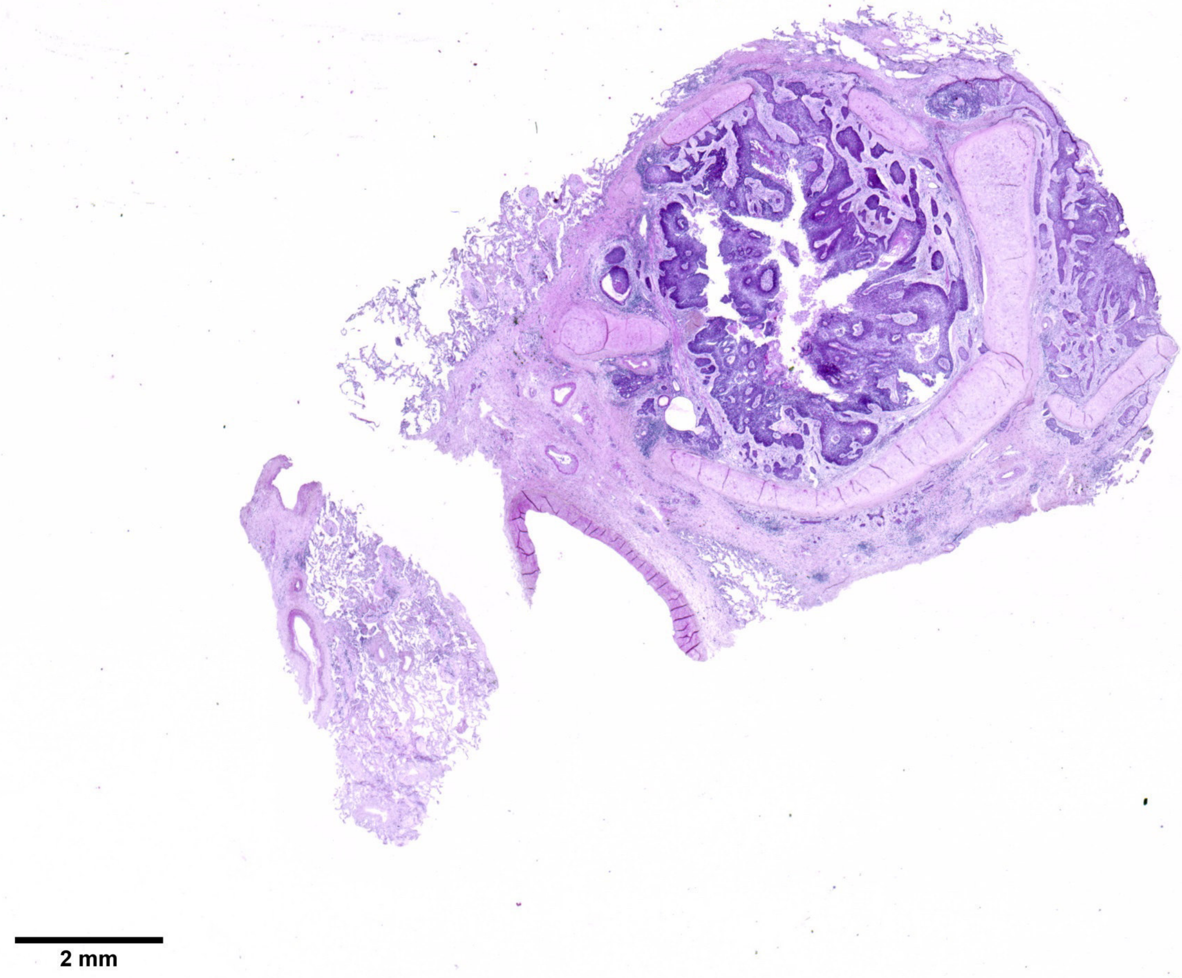

ID18_T

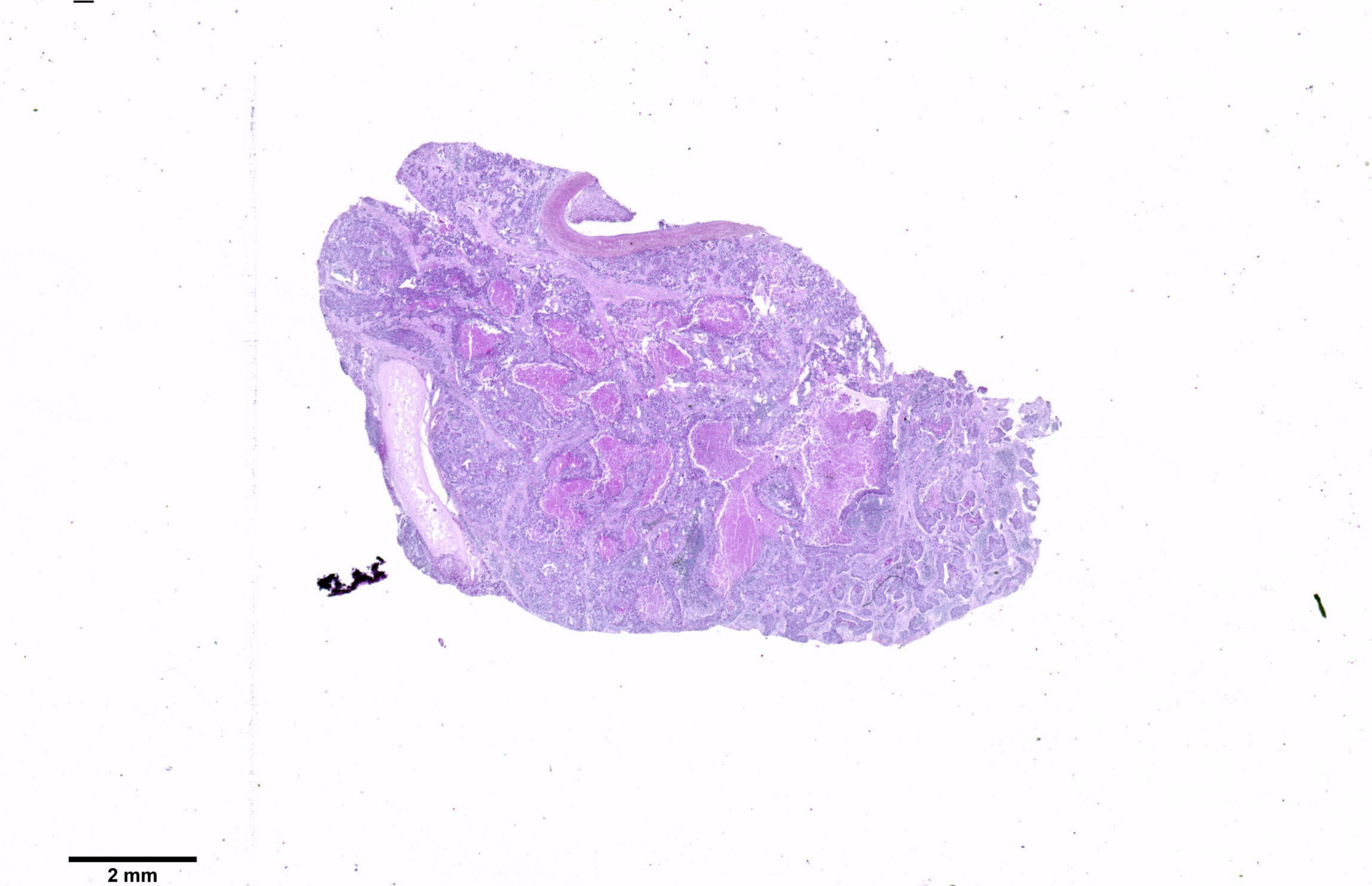

ID19_T

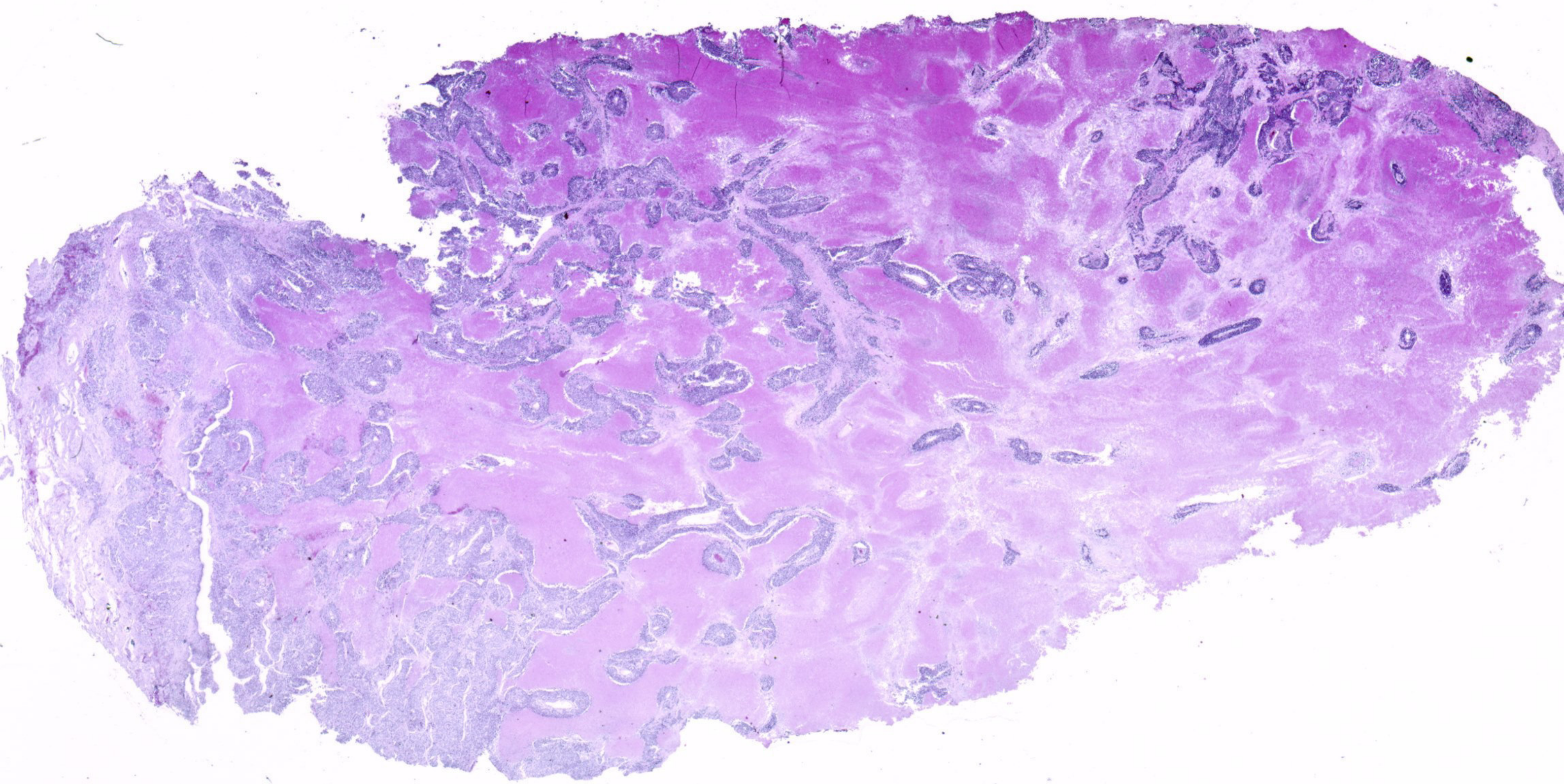

ID22_T

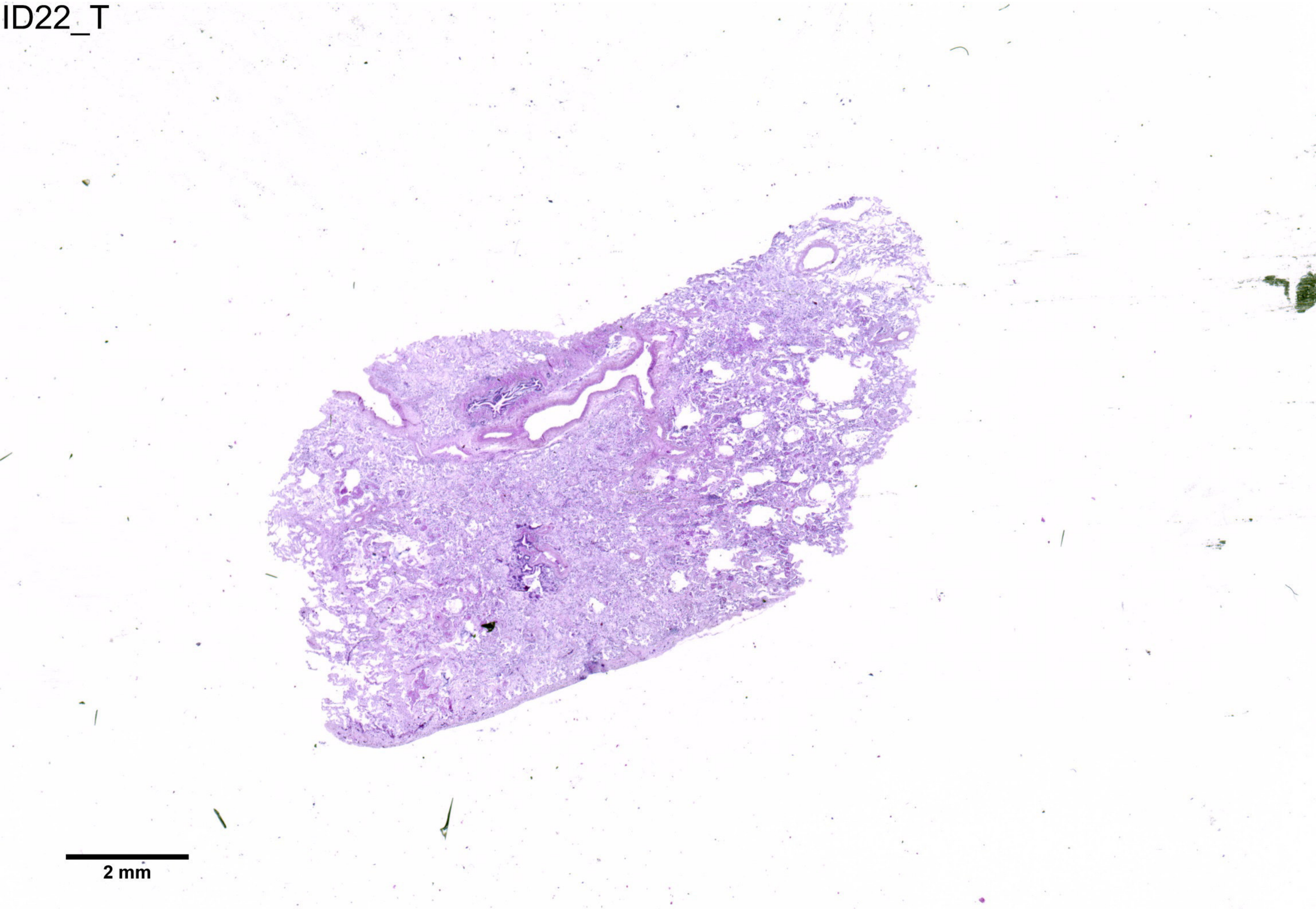

ID25_T

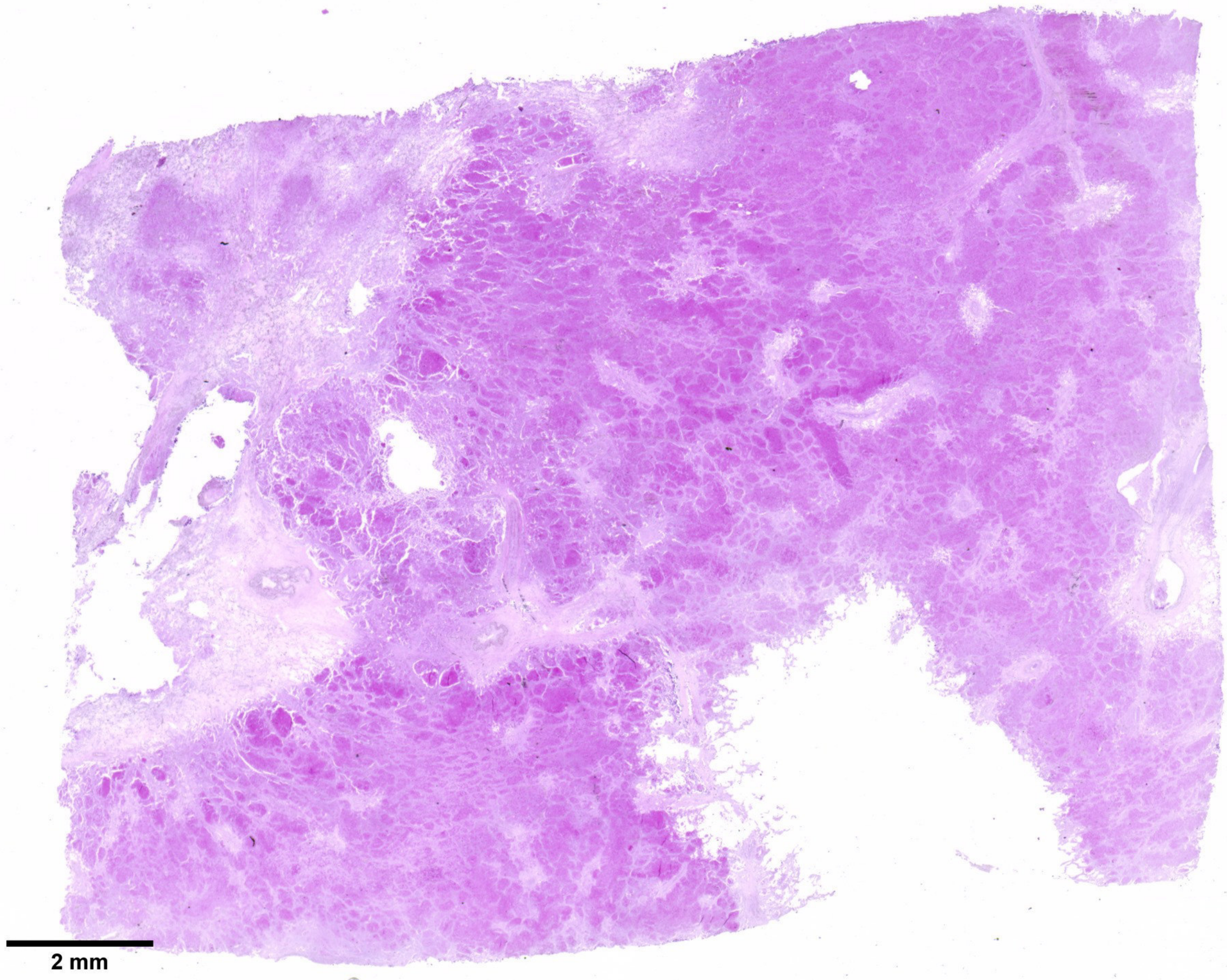

ID29_T

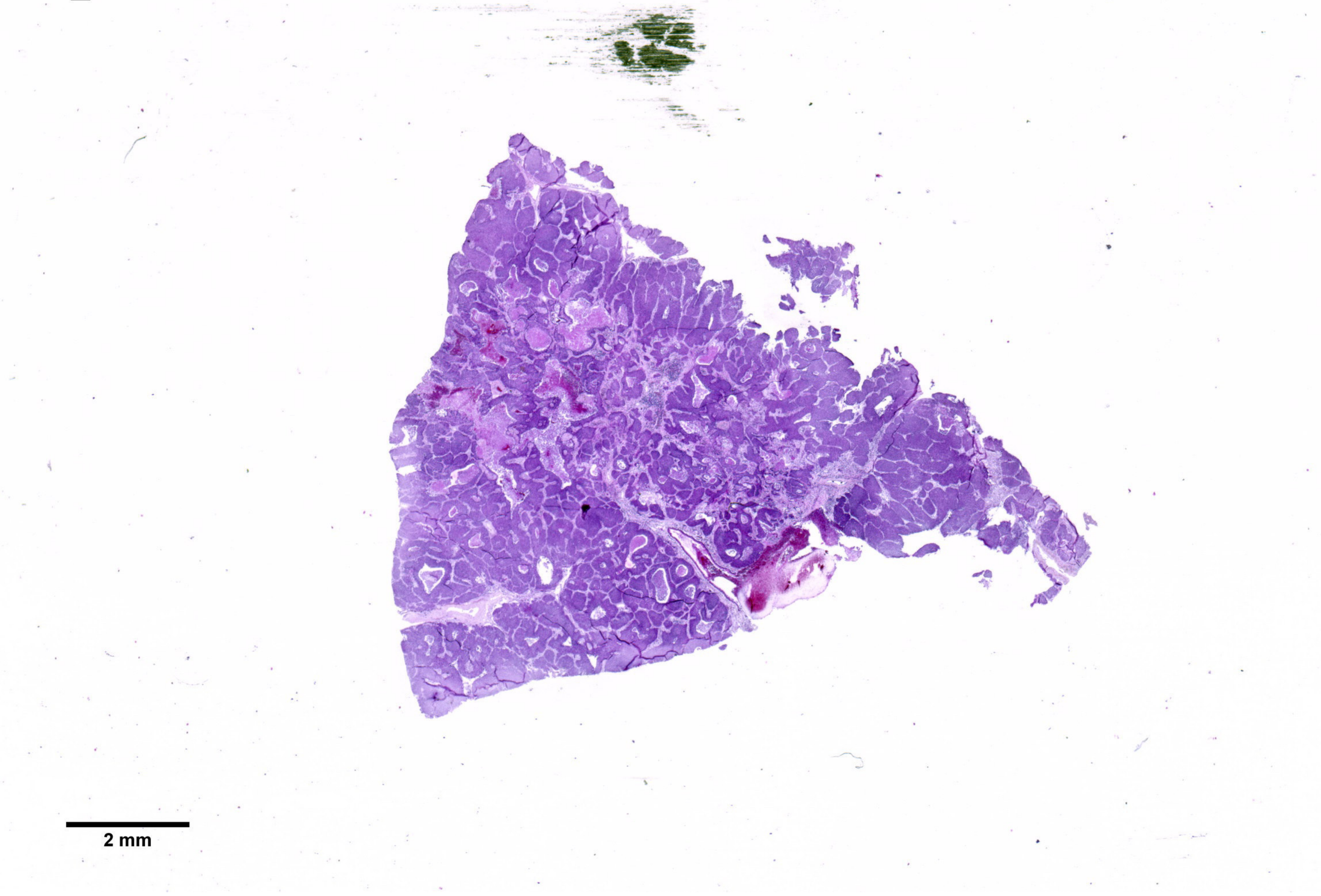

ID30_T

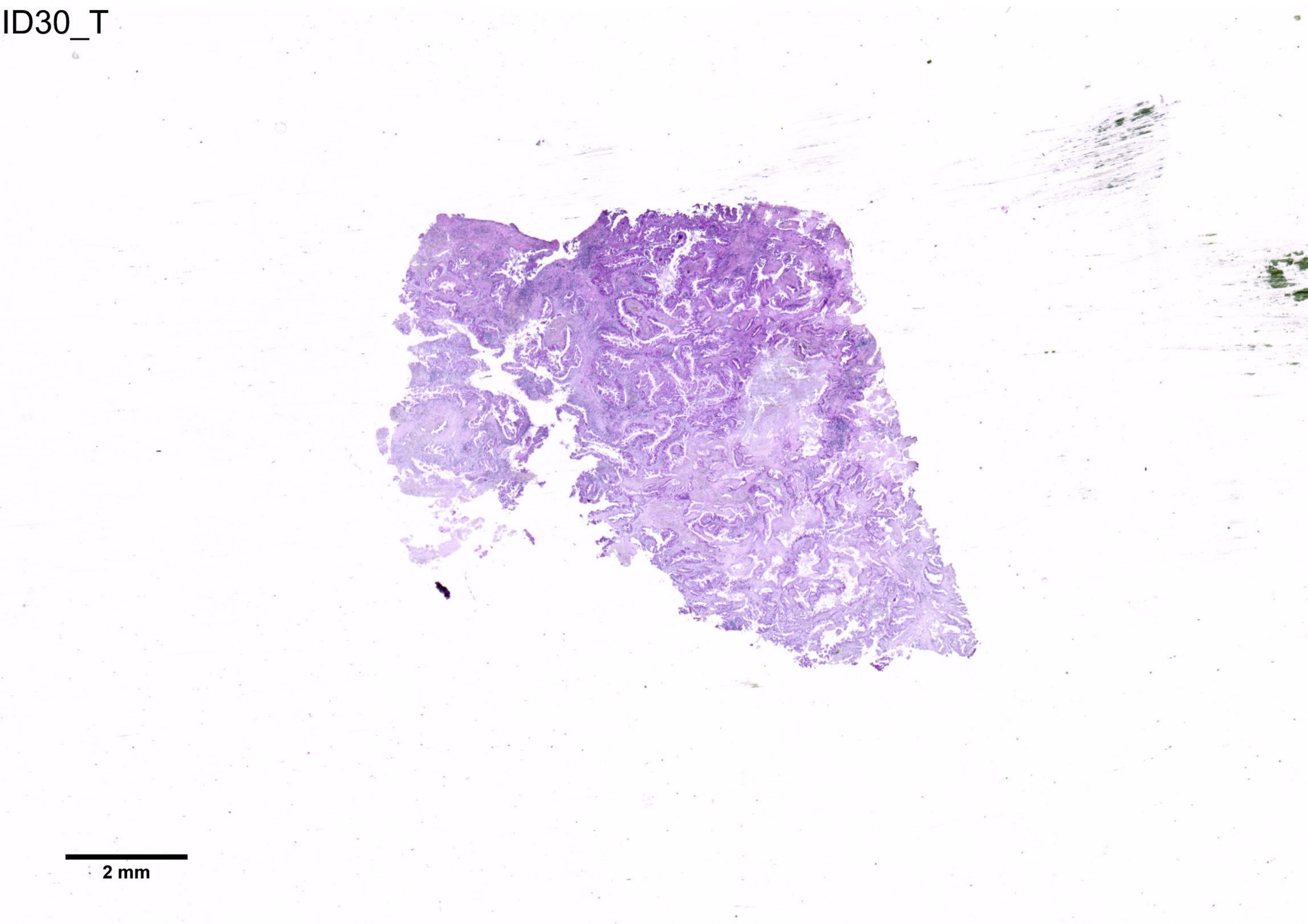

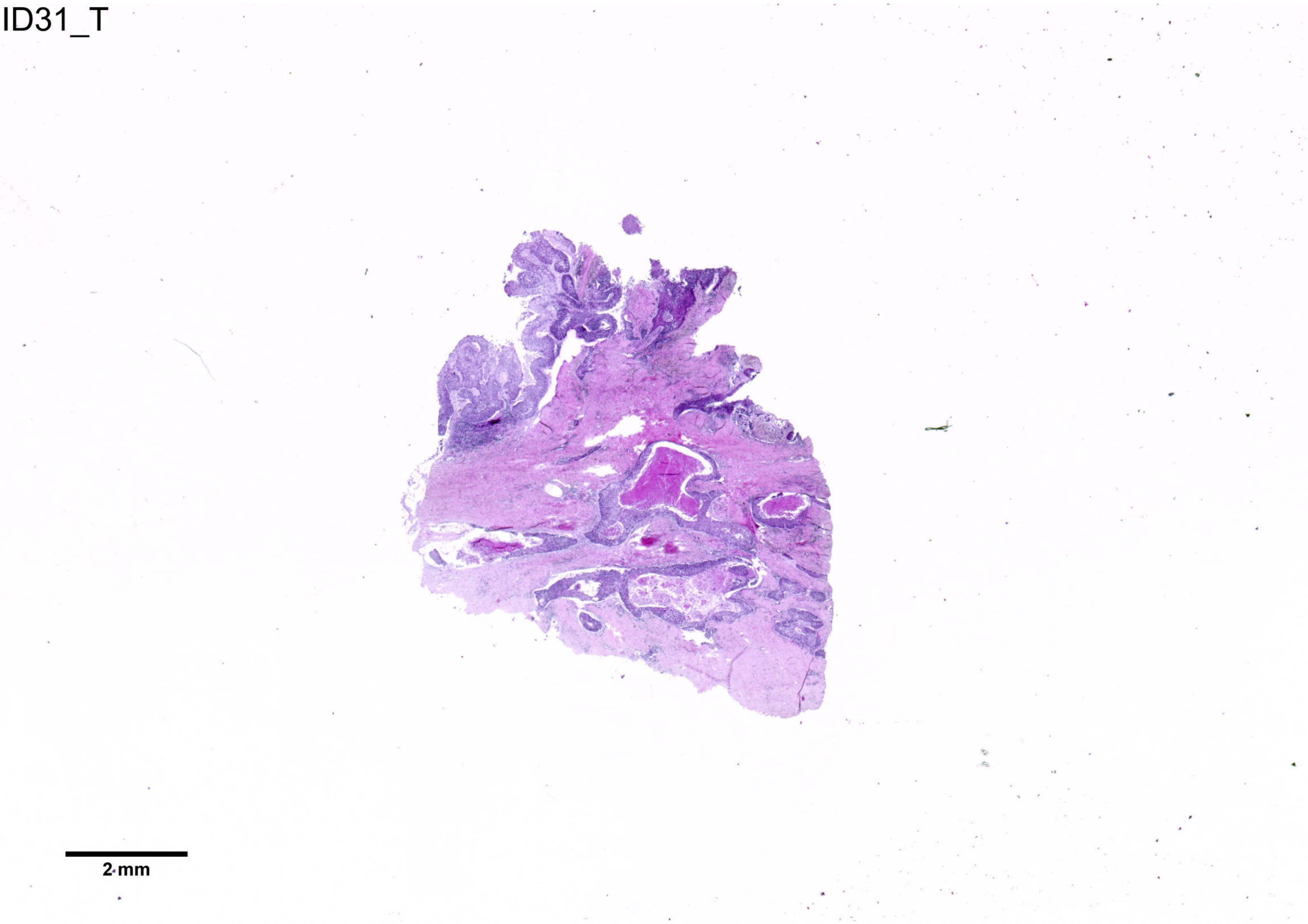
ID31_T
2 mm

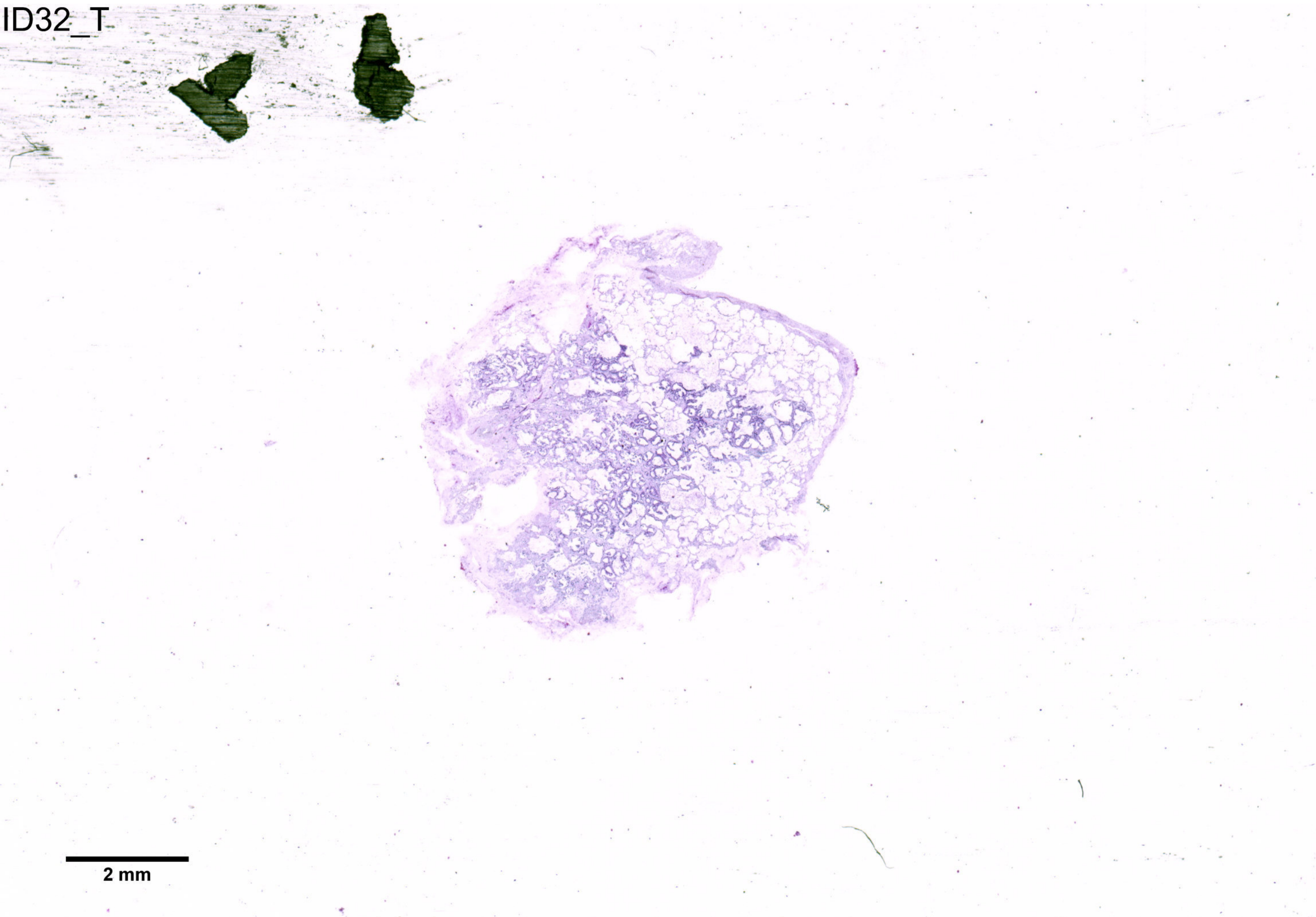
ID32_T
2 mm

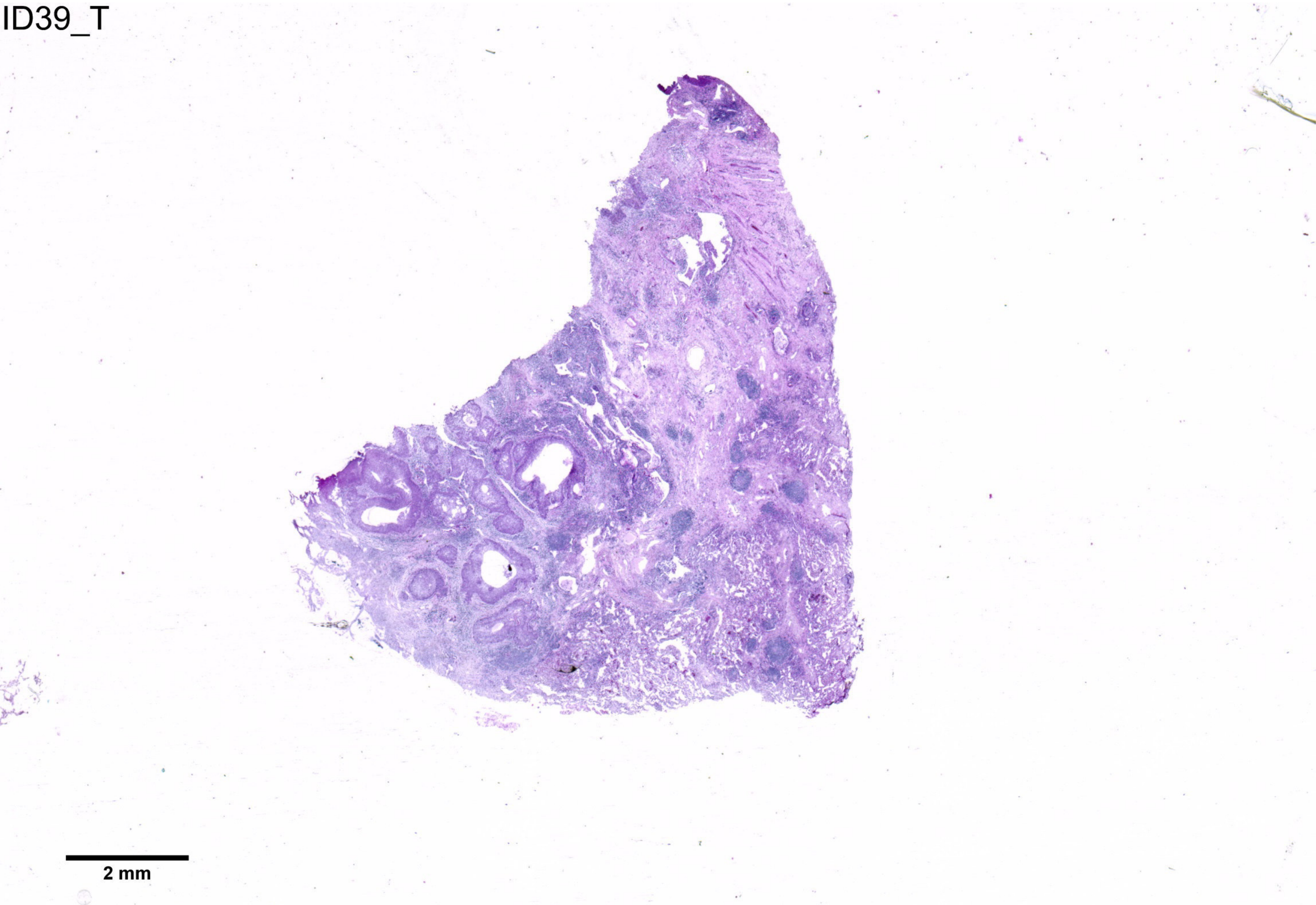
ID39_T
2 mm

ID43_A

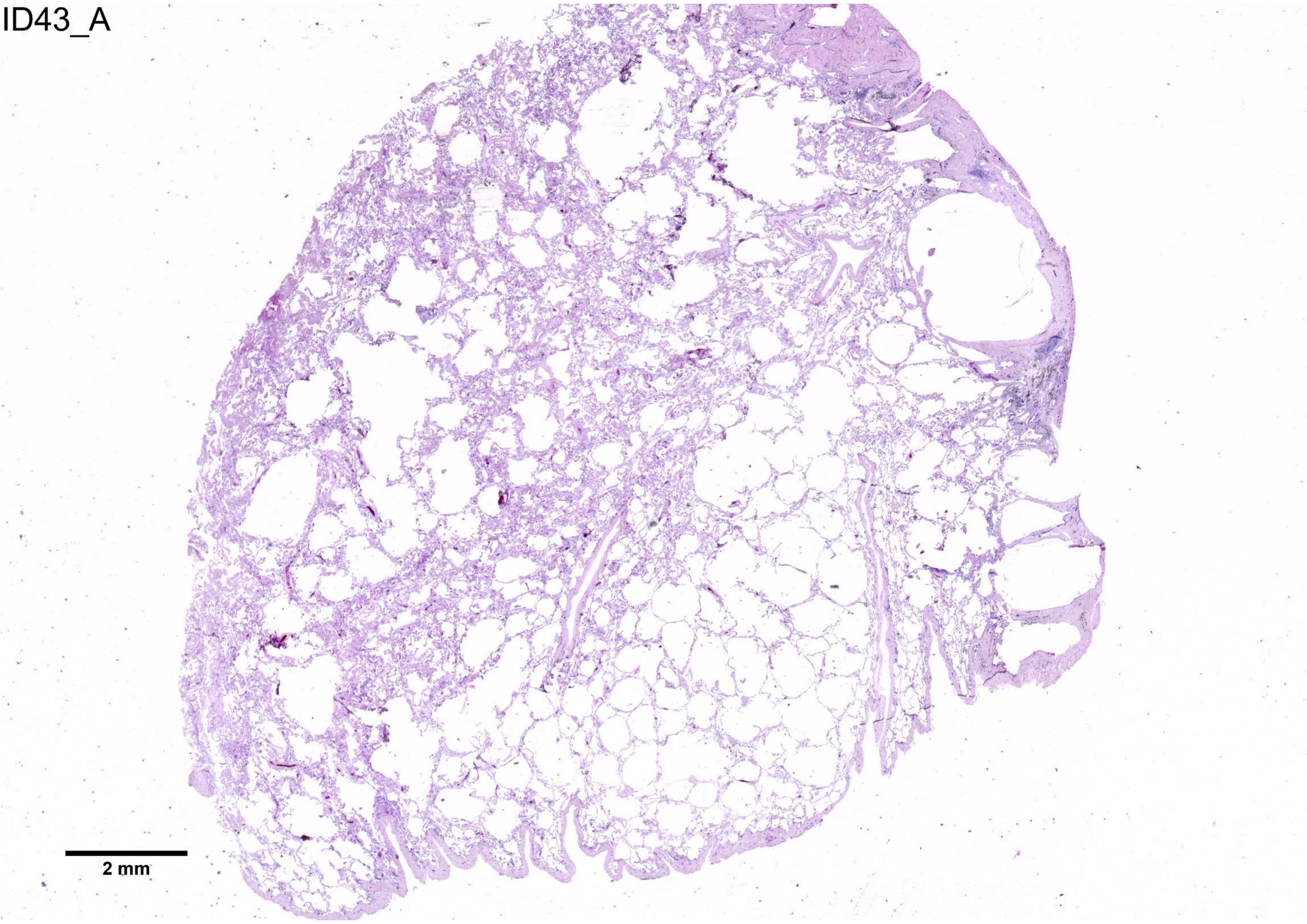

ID43_T

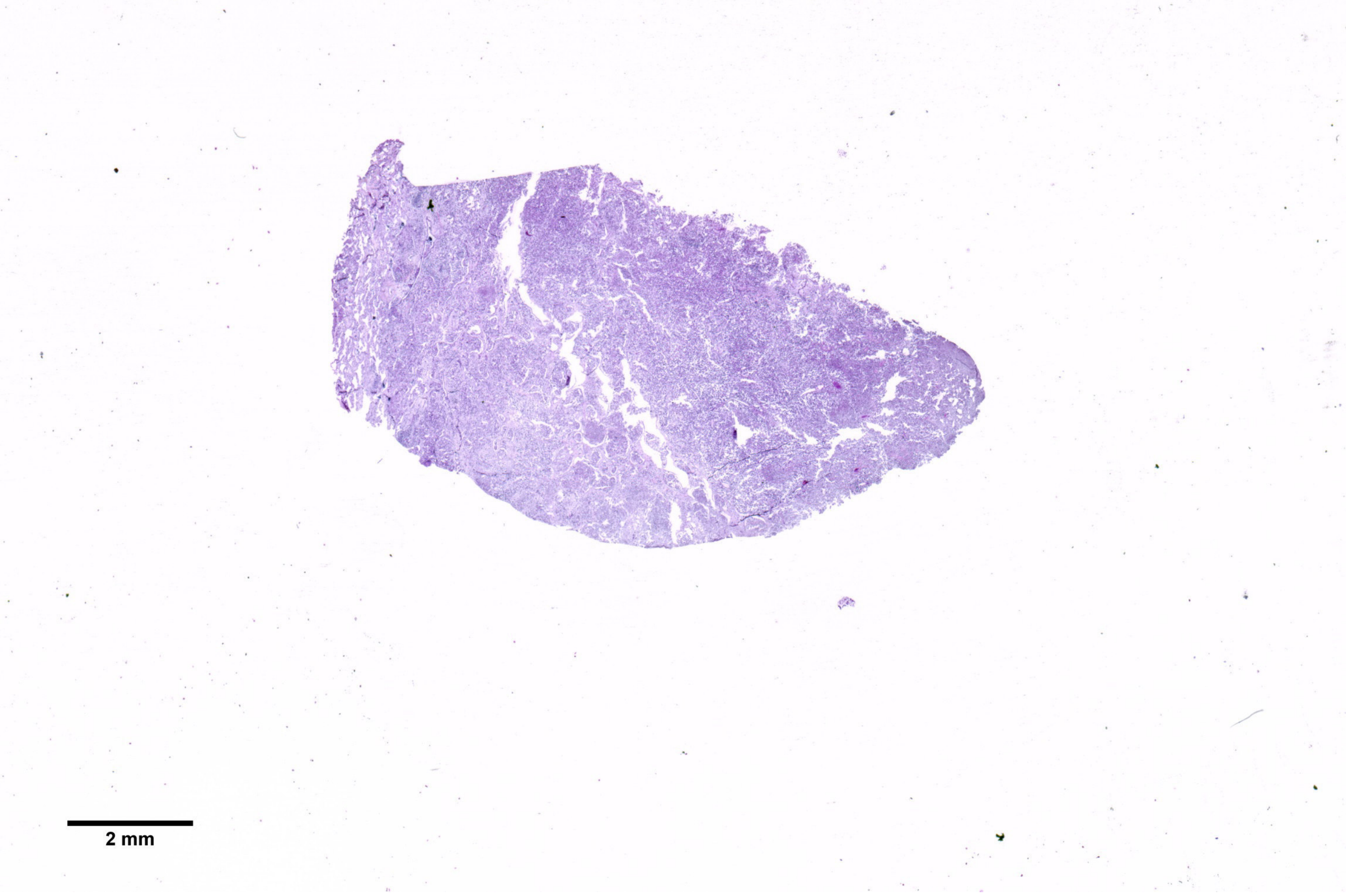

ID50_T

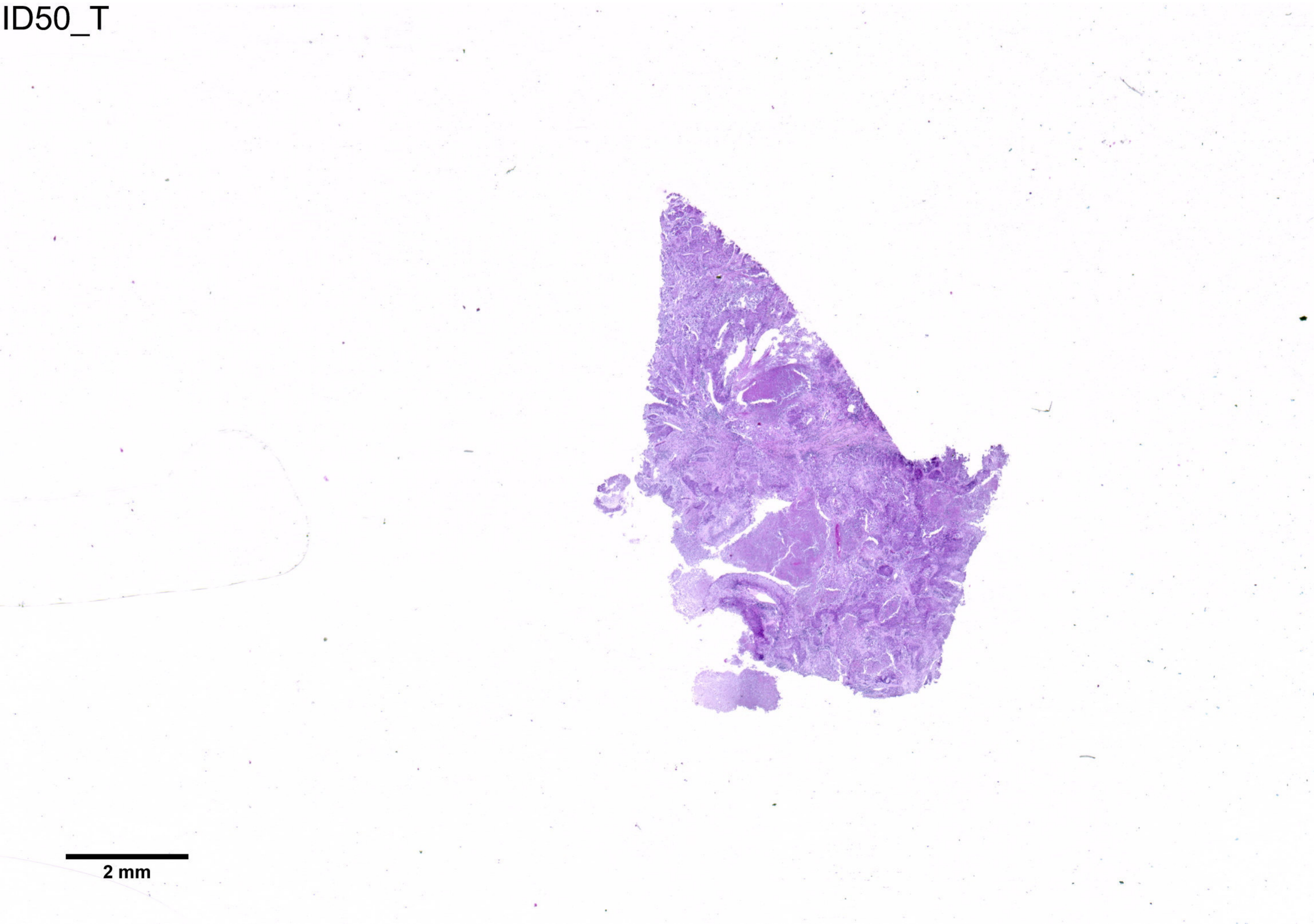

ID53_T

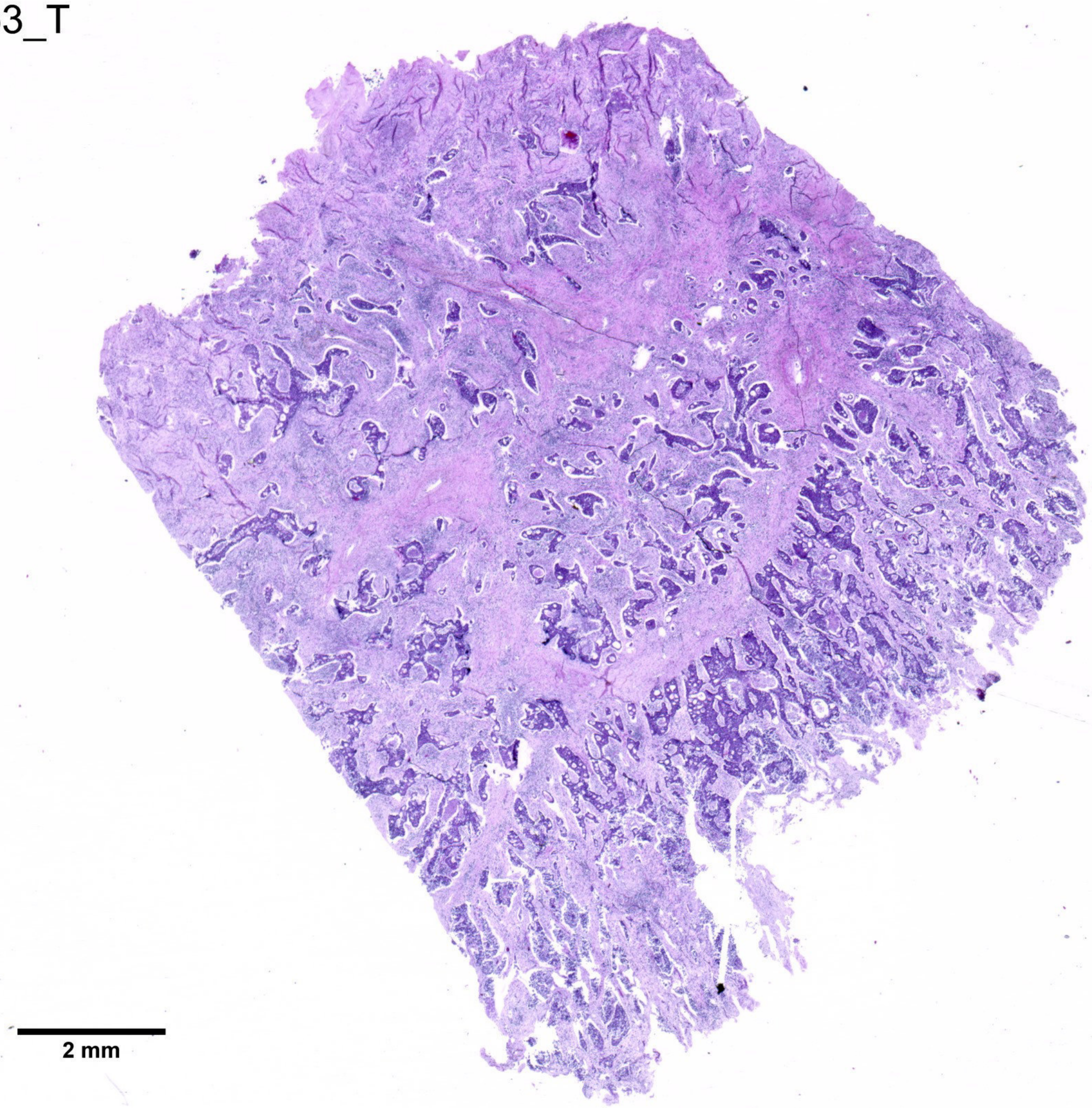

ID61_T

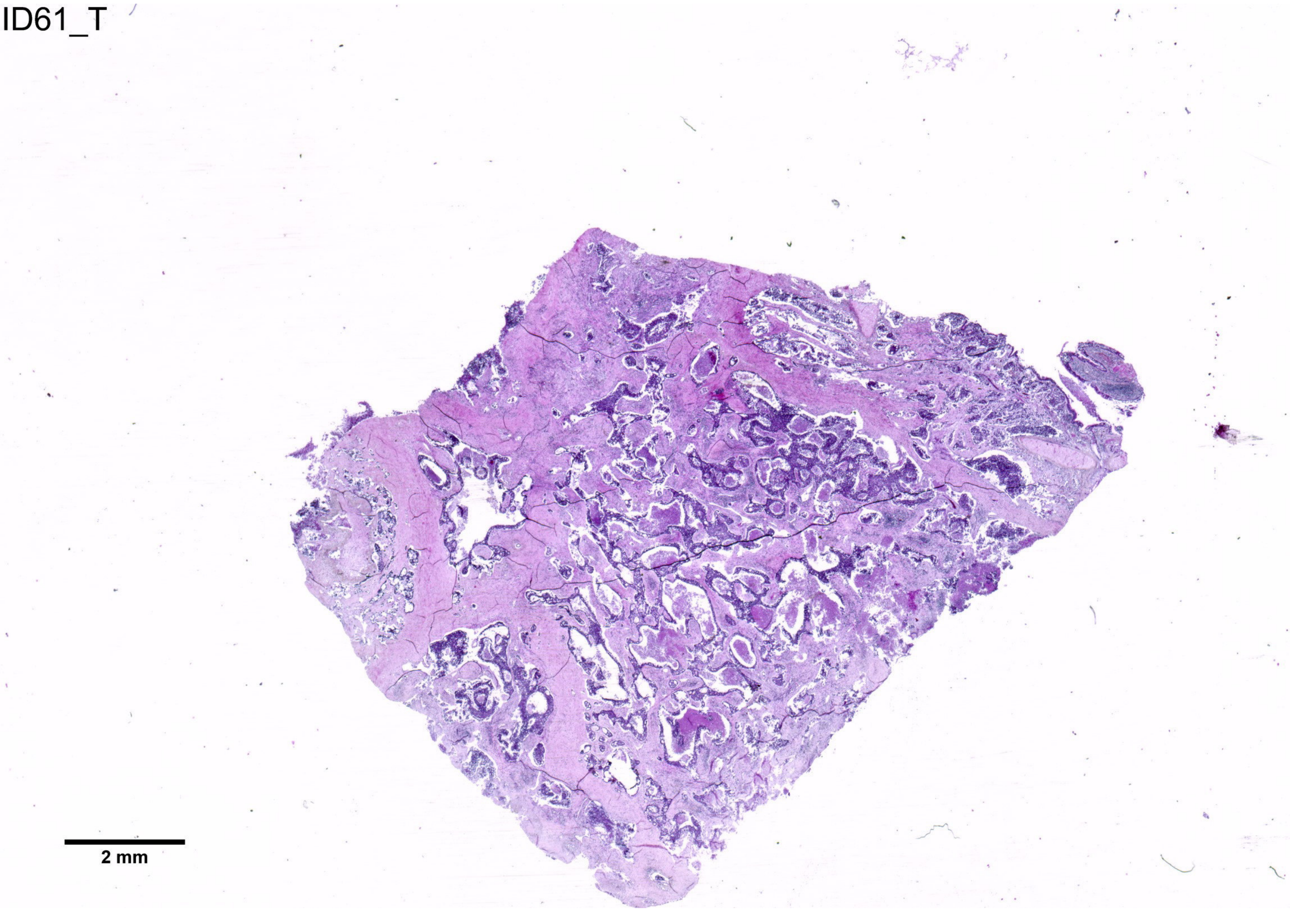

ID64_T

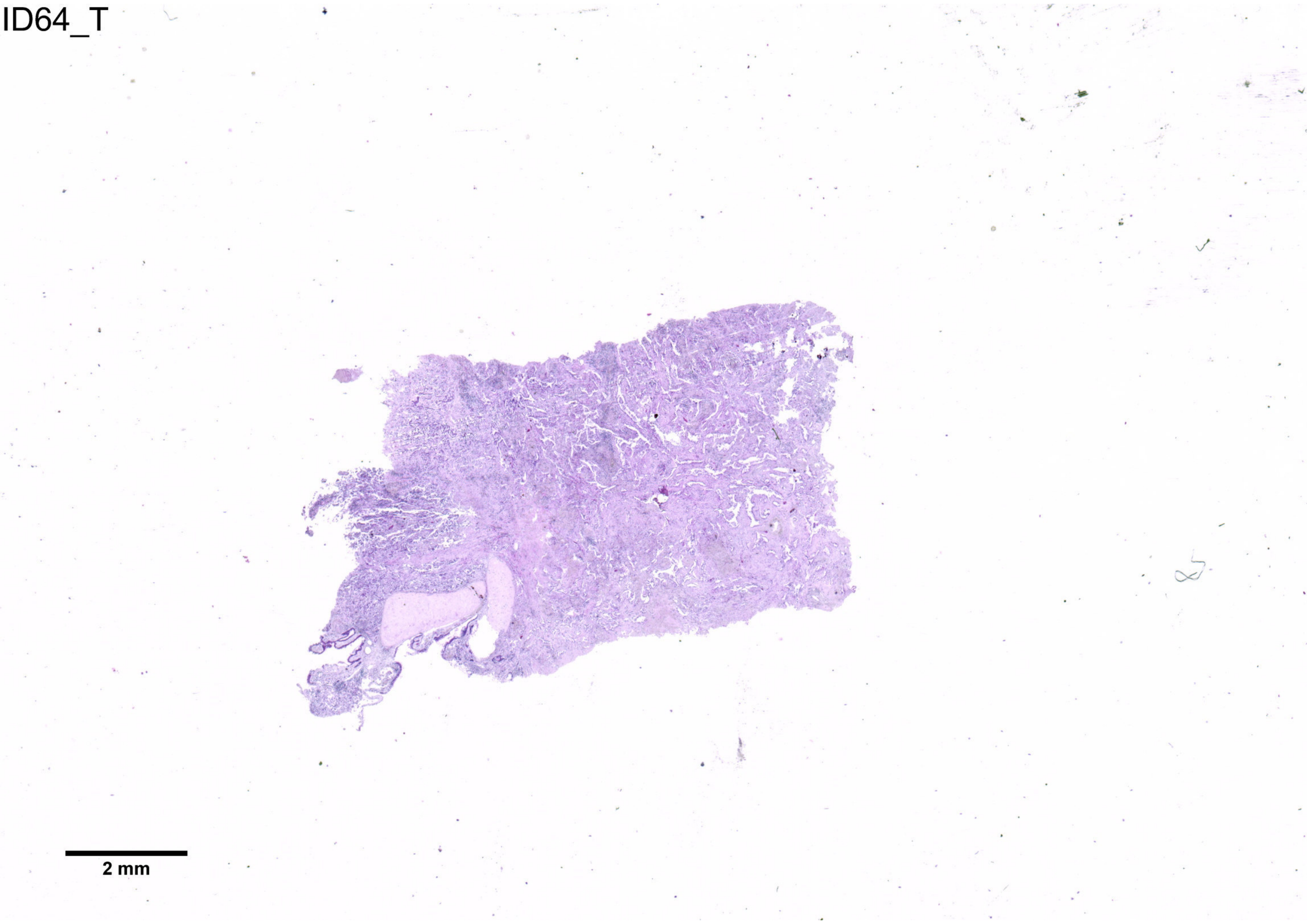

ID66_T

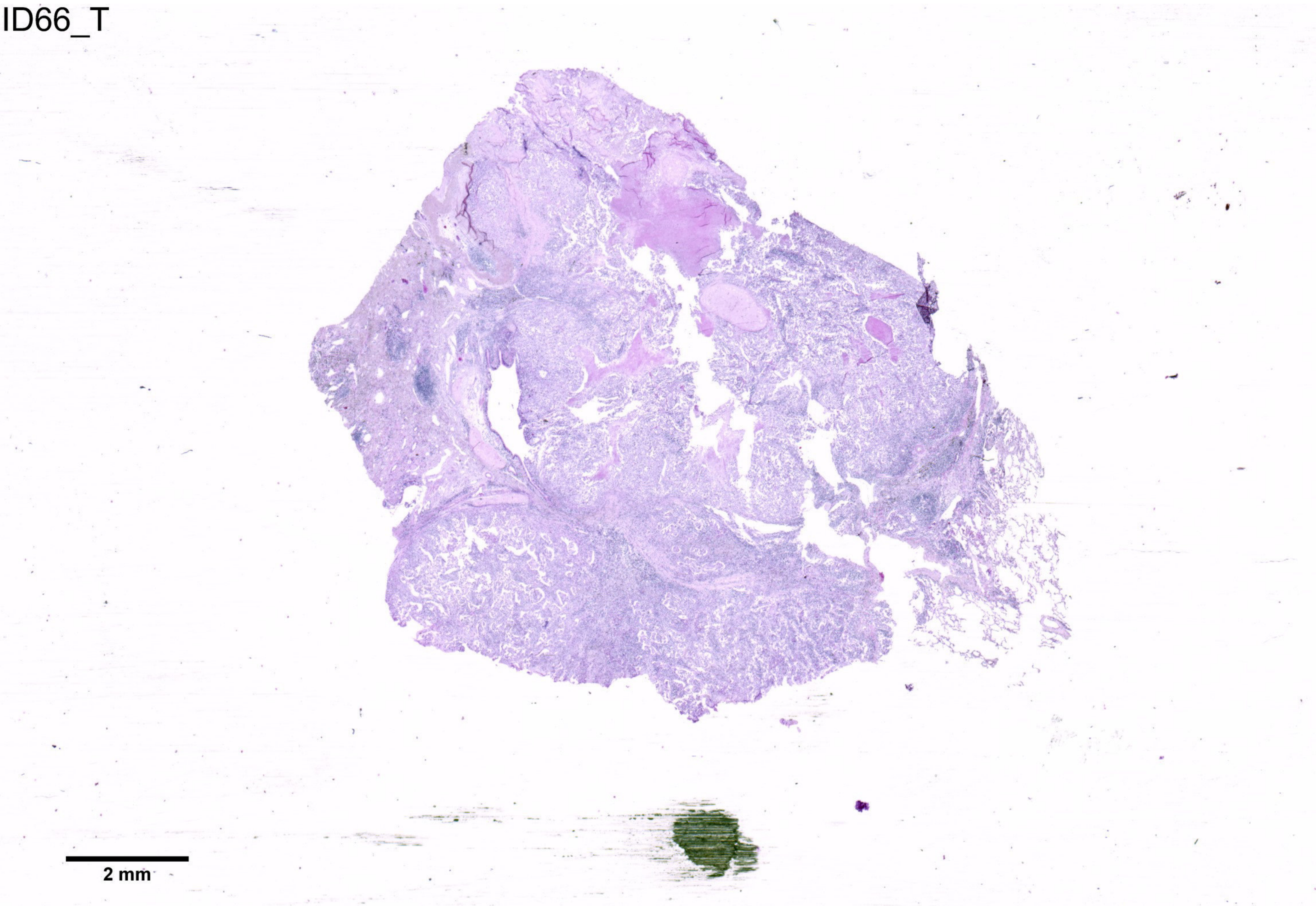

2 mm

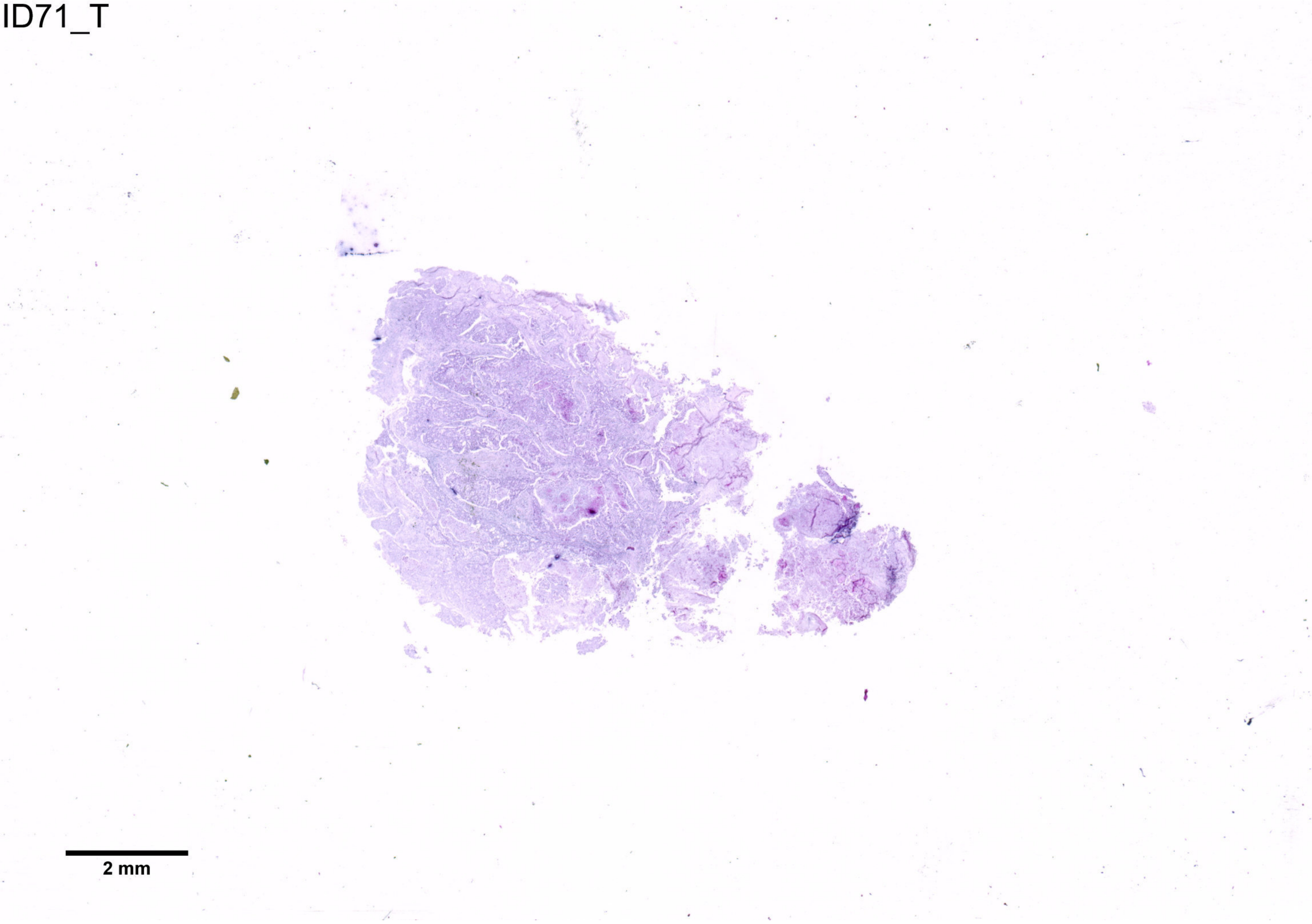
ID71_T
2 mm
