# Supplementary material for: Lipidomes of lung cancer and tumour-free lung tissues reveal distinct molecular signatures for cancer differentiation, age, inflammation, and pulmonary emphysema
Source: Sci Rep. 2017 Sep 11;7:11087. doi: 10.1038/s41598-017-11339-1 (PMC5594029; doi:10.1038/s41598-017-11339-1)
Supplement: Supplementary file 9 — Supplement 9 [file 41598_2017_11339_MOESM9_ESM.doc]

Lipidomes of lung cancer and tumour-free lung tissues reveal distinct molecular signatures for cancer differentiation, age, inflammation, and pulmonary emphysema

Lars F. Eggers1, Julia Müller2, Chakravarthy Marella1, Verena Scholz1, Henrik Watz3,4, Christian Kugler5, Klaus F. Rabe4,5, Torsten Goldmann2,4,# & Dominik Schwudke1,4,#,*

1Research Center Borstel, Bioanalytical Chemistry, Parkallee 1-40, 23845 Borstel, Germany.

2Pathology of the University Hospital of Lübeck and the Research Center Borstel, Location Borstel, Clinical and Experimental Pathology, 23845 Borstel, Germany.

3Pulmonary Research Institute at LungenClinic Großhansdorf, Wöhrendamm 80, 22927 Großhansdorf, Germany.

4Airway Research Center North, German Center for Lung Research, Wöhrendamm 80, 22927 Großhansdorf, Germany.

5LungenClinic Großhansdorf, Wöhrendamm 80, 22927 Großhansdorf, Germany.

#Shared senior authorship.

*Corresponding author: dschwudke@fz-borstel.de

Supplement 9

LipidXplorer data import settings and MFQL scripts for lipid identification

### Import Settings

[FTMS_100000_LE_1]

precursormassshift = 0

precursormassshiftorbi = 0

timerange = (0,1500)

selectionwindow = 0.5

msresolution = 120000

msmsresolution = 2000

mstolerance = 10 ppm

msmstolerance = 10 ppm

msmassrange = (400,1000)

msmsmassrange = (200,2000)

msthreshold = 500

msmsthreshold = 2000

msthresholdtype = absolute

msmsthresholdtype = absolute

msminoccupation = 0.10

msmsminoccupation = 0.5

msresolutiondelta = -150

msmsresolutiondelta = 1

mscalibration = 510.355416,818.736103

msmscalibration =

[FTMS_100000_NEGMS_LE]

precursormassshift = 0

precursormassshiftorbi = 0

timerange = (0,1500)

selectionwindow = 0.5

msresolution = 150000

msmsresolution = 2000

mstolerance = 10 ppm

msmstolerance = 10 ppm

msmassrange = (400,1800)

msmsmassrange = (200,2000)

msthreshold = 500

msmsthreshold = 2000

msthresholdtype = absolute

msmsthresholdtype = absolute

msminoccupation = 0.1

msmsminoccupation = 0.5

msresolutiondelta = -100

msmsresolutiondelta = 1

mscalibration = 609.376211,774.673502

msmscalibration =

### MFQL negative ion mode

QUERYNAME = Ceramides;

DEFINE PR = 'C[24..42] H[30..200] N[1] O[3..4] Cl[1]' WITH DBR = (0.5,8.5), CHG = -1;

IDENTIFY

# marking

PR IN MS1-

REPORT

MASS = "PR.mass;

CHEMSC = PR.chemsc;

ERRppm = "%2.2f" % "(PR.errppm)";

SPECIESUM = "Cer [%d:%d;%d]" % "((PR.chemsc)[C], (PR.chemsc)[db] - 0.5, (PR.chemsc)[O]-3,)";

INTENS = PR.intensity;

;

################ end script ##################

##########################################################

# Identify CL with checking the precursor mass -2 charge #

##########################################################

QUERYNAME = Cardiolipin;

DEFINE PR = 'C[65..87] H[90..180] O[17] P[2]' WITH DBR = (5,16), CHG = -2;

IDENTIFY

# marking

PR IN MS1-

REPORT

MASS = "PR.mass;

CHEMSC = PR.chemsc;

ERRppm = "%2.2f" % "(PR.errppm)";

SPECIESUM = "CL [%d:%d]" % "((PR.chemsc)[C] - 9, (PR.chemsc)[db] - 5)";

INTENS = PR.intensity;;

################ end script ##################

QUERYNAME = Diacylgylcerol;

DEFINE prDAG = 'C[27..51] H[10..130] O[5] Cl[1]' WITH DBR = (1.5,13.5), CHG = -1;

IDENTIFY

# marking

prDAG IN MS1-

REPORT

MASS = "prDAG.mass;

CHEMSC = prDAG.chemsc;

ERRppm = "%2.2f" % "(prDAG.errppm)";

SPECIESUM = "DAG [%d:%d]" % "((prDAG.chemsc)[C] - 3, (prDAG.chemsc)[db] - 1.5)";

INTENS = prDAG.intensity;;

################ end script ##################

QUERYNAME = HexosylCeramides;

DEFINE PR = 'C[24..48] H[30..200] N[1] O[8..9] Cl[1]' WITH DBR = (1.5,5.5), CHG = -1;

IDENTIFY

# marking

PR IN MS1-

REPORT

MASS = "PR.mass;

CHEMSC = PR.chemsc;

ERRppm = "%2.2f" % "(PR.errppm)";

SPECIESUM = "HexCer [%d:%d;%d]" % "((PR.chemsc)[C]-6, (PR.chemsc)[db] - 1.5, (PR.chemsc)[O]-8)";

INTENS = PR.intensity;

;

################ end script ##################

QUERYNAME = Lysophosphatidylcholine;

DEFINE prLPC = 'C[20..32] H[15..90] O[7] N[1] P[1] Cl[1]' WITH DBR = (0.5,6.5), CHG = -1;

IDENTIFY

# marking

prLPC IN MS1-

REPORT

MASS = "prLPC.mass;

CHEMSC = prLPC.chemsc;

ERRppm = "%2.2f" % "(prLPC.errppm)";

SPECIESUM = "LPC [%d:%d]" % "((prLPC.chemsc)[C] - 8, (prLPC.chemsc)[db] - 0.5)";

INTENS = prLPC.intensity;;

################ end script ##################

QUERYNAME = Lysophosphatidylethanolamine;

DEFINE prLPE = 'C[17..29] H[15..100] O[7] N[1] P[1]' WITH DBR = (1.5,7.5), CHG = -1;

IDENTIFY

# marking

prLPE IN MS1-

REPORT

MASS = "prLPE.mass;

CHEMSC = prLPE.chemsc;

ERRppm = "%2.2f" % "(prLPE.errppm)";

SPECIESUM = "LPE [%d:%d]" % "((prLPE.chemsc)[C] - 5, (prLPE.chemsc)[db] - 1.5)";

INTENS = prLPE.intensity;;

################ end script ##################

QUERYNAME = LysoPhosphatidylglycerols;

DEFINE prLPG = 'C[18..30] H[20..200] O[9] P[1]' WITH DBR = (1.5,13.5), CHG = -1;

IDENTIFY

# marking

prLPG IN MS1-

REPORT

MASS = "prLPG.mass;

CHEMSC = prLPG.chemsc;

ERRppm = "%2.2f" % "(prLPG.errppm)";

SPECIESUM = "LPG [%d:%d]" % "((prLPG.chemsc)[C] - 6, (prLPG.chemsc)[db] - 1.5)";

INTENS = prLPG.intensity;;

################ end script ##################

QUERYNAME = LysoPhosphatidylinositol;

DEFINE prLPI = 'C[21..33] H[20..140] O[13] P[1]' WITH DBR = (2.5,14.5), CHG = -1;

IDENTIFY

# marking

prLPI IN MS1-

REPORT

MASS = "prLPI.mass;

CHEMSC = prLPI.chemsc;

ERRppm = "%2.2f" % "(prLPI.errppm)";

SPECIESUM = "LPI [%d:%d]" % "((prLPI.chemsc)[C] - 9, (prLPI.chemsc)[db] - 2.5)";

INTENS = prLPI.intensity;;

################ end script ##################

QUERYNAME = LysoPhosphatidylserine;

DEFINE prLPS = 'C[18..30] H[30..130] O[9] N[1] P[1]' WITH DBR = (2.5,14.5), CHG = -1;

IDENTIFY

# marking

prLPS IN MS1-

REPORT

MASS = "prLPS.mass;

CHEMSC = prLPS.chemsc;

ERRppm = "%2.2f" % "(prLPS.errppm)";

SPECIESUM = "LPS [%d:%d]" % "((prLPS.chemsc)[C] - 6, (prLPS.chemsc)[db] - 2.5)";

INTENS = prLPS.intensity;;

################ end script ##################

QUERYNAME = PhosphaticAcid;

DEFINE prPA = 'C[27..51] H[30..130] O[8] P[1]' WITH DBR = (2.5,14.5), CHG = -1;

IDENTIFY

# marking

prPA IN MS1-

REPORT

MASS = "prPA.mass;

CHEMSC = prPA.chemsc;

ERRppm = "%2.2f" % "(prPA.errppm)";

SPECIESUM = "PA [%d:%d]" % "((prPA.chemsc)[C] - 3, (prPA.chemsc)[db] - 2.5)";

INTENS = prPA.intensity;;

################ end script ##################

QUERYNAME = Phosphatidylcholine;

DEFINE prPC = 'C[32..56] H[30..130] O[8] N[1] P[1] Cl[1]' WITH DBR = (1.5,13.5), CHG = -1;

IDENTIFY

# marking

prPC IN MS1-

REPORT

MASS = "prPC.mass;

CHEMSC = prPC.chemsc;

ERRppm = "%2.2f" % "(prPC.errppm)";

SPECIESUM = "PC [%d:%d]" % "((prPC.chemsc)[C] - 8, (prPC.chemsc)[db] - 1.5)";

INTENS = prPC.intensity;;

################ end script ##################

QUERYNAME = PCOO;

DEFINE PR = 'C[48] H[100] O[6] N[1] P[1] Cl[1]' WITH DBR = (0.5,0.5), CHG = -1;

IDENTIFY

# marking

PR IN MS1-

SUCHTHAT

isEven(PR.chemsc[C])

REPORT

MASS = "PR.mass;

CHEMSC = PR.chemsc;

ERRppm = "%2.2f" % "(PR.errppm)";

SPECIESUM = "PC-IS";

INTENS = PR.intensity;;

################ end script ##################

QUERYNAME = Phosphatidylcholineether;

DEFINE prPCO = 'C[32..56] H[30..130] O[7] N[1] P[1] Cl[1]' WITH DBR = (0.5,12.5), CHG = -1;

IDENTIFY

# marking

prPCO IN MS1-

REPORT

MASS = "prPCO.mass;

CHEMSC = prPCO.chemsc;

ERRppm = "%2.2f" % "(prPCO.errppm)";

SPECIESUM = "PC-O [%d:%d]" % "((prPCO.chemsc)[C] - 8, (prPCO.chemsc)[db] - 0.5)";

INTENS = prPCO.intensity;;

################ end script ##################

QUERYNAME = Phosphatidylethanolamine;

DEFINE prPE = 'C[29..53] H[30..130] O[8] N[1] P[1]' WITH DBR = (2.5,14.5), CHG = -1;

IDENTIFY

# marking

prPE IN MS1-

REPORT

MASS = "prPE.mass;

CHEMSC = prPE.chemsc;

ERRppm = "%2.2f" % "(prPE.errppm)";

SPECIESUM= "PE [%d:%d]" % "((prPE.chemsc)[C] - 5, (prPE.chemsc)[db] - 2.5)";

INTENS = prPE.intensity;;

################ end script ##################

QUERYNAME = PEOO;

DEFINE PR = 'C[45] H[93] O[6] N[1] P[1]' WITH DBR = (0.5,0.5), CHG = -1;

IDENTIFY

# marking

PR IN MS1-

SUCHTHAT

isOdd(PR.chemsc[C])

REPORT

MASS = "PR.mass;

CHEMSC = PR.chemsc;

ERRppm = "%2.2f" % "(PR.errppm)";

SPECIESUM = "PE-IS";

INTENS = PR.intensity;;

################ end script ##################

QUERYNAME = Phosphatidylethanolamineether;

DEFINE prPEO = 'C[29..53] H[30..150] O[7] N[1] P[1]' WITH DBR = (1.5,13.5), CHG = -1;

IDENTIFY

# marking

prPEO IN MS1-

REPORT

MASS = "prPEO.mass;

CHEMSC = prPEO.chemsc;

ERRppm = "%2.2f" % "(prPEO.errppm)";

SPECIESUM = "PE-O [%d:%d]" % "((prPEO.chemsc)[C] - 5, (prPEO.chemsc)[db] - 1.5)";

INTENS = prPEO.intensity;;

################ end script ##################

QUERYNAME = Phosphatidylglycerols;

DEFINE prPG = 'C[20..54] H[20..200] O[10] P[1]' WITH DBR = (2.5,14.5), CHG = -1;

IDENTIFY

# marking

prPG IN MS1-

REPORT

MASS = "prPG.mass;

CHEMSC = prPG.chemsc;

ERRppm = "%2.2f" % "(prPG.errppm)";

SPECIESUM = "PG [%d:%d]" % "((prPG.chemsc)[C] - 6, (prPG.chemsc)[db] - 2.5)";

INTENS = prPG.intensity;;

################ end script ##################

QUERYNAME = Phosphatidylinositol;

DEFINE prPI = 'C[33..57] H[30..140] O[13] P[1]' WITH DBR = (3.5,15.5), CHG = -1;

IDENTIFY

# marking

prPI IN MS1-

REPORT

MASS = "prPI.mass;

CHEMSC = prPI.chemsc;

ERRppm = "%2.2f" % "(prPI.errppm)";

SPECIESUM = "PI [%d:%d]" % "((prPI.chemsc)[C] - 9, (prPI.chemsc)[db] - 3.5)";

INTENS = prPI.intensity;;

################ end script ##################

QUERYNAME = Phosphatidylserine;

DEFINE prPS = 'C[30..54] H[30..130] O[10] N[1] P[1]' WITH DBR = (3.5,15.5), CHG = -1;

IDENTIFY

# marking

prPS IN MS1-

REPORT

MASS = "prPS.mass;

CHEMSC = prPS.chemsc;

ERRppm = "%2.2f" % "(prPS.errppm)";

SPECIESUM = "PS [%d:%d]" % "((prPS.chemsc)[C] - 6, (prPS.chemsc)[db] - 3.5)";

INTENS = prPS.intensity;;

################ end script ##################

QUERYNAME = Sphingomyelin;

DEFINE prSM = 'C[29..42] H[30..130] O[6..7] N[2] P[1] Cl[1]' WITH DBR = (0.5,3.5), CHG = -1;

IDENTIFY

# marking

prSM IN MS1-

REPORT

MASS = "prSM.mass;

CHEMSC = prSM.chemsc;

ERRppm = "%2.2f" % "(prSM.errppm)";

SPECIESUM = "SM [%d:%d;%d]" % "((prSM.chemsc)[C] - 5, (prSM.chemsc)[db] - 0.5, (prSM.chemsc)[O] - 6)";

INTENS = prSM.intensity;;

################ end script ##################

QUERYNAME = Triacylglycerol;

DEFINE prTAG = 'C[39..75] H[20..200] O[6] Cl[1]' WITH DBR = (2.5,20.5), CHG = -1;

IDENTIFY

# marking

prTAG IN MS1-

REPORT

MASS = "prTAG.mass;

CHEMSC = prTAG.chemsc;

ERRppm = "%2.2f" % "(prTAG.errppm)";

SPECIESUM = "TAG [%d:%d]" % "((prTAG.chemsc)[C] - 3, (prTAG.chemsc)[db] - 2.5)";

INTENS = prTAG.intensity;;

################ end script ##################

### MFQL positive ion mode

QUERYNAME = cholesterylester;

DEFINE PR = 'C[39..50] H[30..200] N[1] O[2]' WITH DBR = (4.5,10.5), CHG = 1;

IDENTIFY

# marking

PR IN MS1+

REPORT

MASS = "PR.mass;

CHEMSC = PR.chemsc;

ERRppm = "%2.2f" % "(PR.errppm)";

SPECIESUM = "CE [%d:%d]" % "((PR.chemsc)[C] - 27, (PR.chemsc)[db] - 4.5)";

INTENS = PR.intensity;;

################ end script ##################

QUERYNAME = ceramides;

DEFINE PR = 'C[24..50] H[30..200] N[1] O[3..5] Na[0..1]' WITH DBR = (0.5,7.5), CHG = +1;

IDENTIFY

# marking

PR IN MS1+

SUCHTHAT

isEven(PR.chemsc[C])

REPORT

MASS = "PR.mass;

CHEMSC = PR.chemsc;

ERRppm = "%2.2f" % "(PR.errppm)";

SPECIESUM = "Cer [%d:%d;%d]" % "((PR.chemsc)[C], (PR.chemsc)[db] - 0.5, (PR.chemsc)[O]-3)";

INTENS = PR.intensity;

;

################ end script ##################

QUERYNAME = hexosylceramides;

DEFINE PR = 'C[24..70] H[30..200] N[1] O[8..9] Na[0..1]' WITH DBR = (1.5,19.5), CHG = +1;

IDENTIFY

# marking

PR IN MS1+

SUCHTHAT

isEven(PR.chemsc[C])

REPORT

MASS = "PR.mass;

CHEMSC = PR.chemsc;

ERRppm = "%2.2f" % "(PR.errppm)";

SPECIESUM = "HexCer [%d:%d;%d]" % "((PR.chemsc)[C]-6, (PR.chemsc)[db] - 1.5, (PR.chemsc)[O]-8)";

INTENS = PR.intensity;

;

################ end script ##################

QUERYNAME = Lysophosphatidylcholine;

DEFINE prLPC = 'C[20..32] H[30..130] O[7] N[1] P[1] Na[0..1]' WITH DBR = (0.5,6.5), CHG = +1;

IDENTIFY

# marking

prLPC IN MS1+

SUCHTHAT

isEven(prLPC.chemsc[C])

REPORT

MASS = "prLPC.mass;

CHEMSC = prLPC.chemsc;

ERRppm = "%2.2f" % "(prLPC.errppm)";

SPECIESUM = "LPC [%d:%d]" % "((prLPC.chemsc)[C] - 8, (prLPC.chemsc)[db] - 0.5)";

INTENS = prLPC.intensity;;

################ end script ##################

QUERYNAME = LysoPhosphatidylethanolamine;

DEFINE prLPE = 'C[17..29] H[15..80] O[7] N[1] P[1] Na[0..1]' WITH DBR = (0.5,6.5), CHG = +1;

IDENTIFY

# marking

prLPE IN MS1+

SUCHTHAT

isOdd(prLPE.chemsc[C])

REPORT

MASS = "prLPE.mass;

CHEMSC = prLPE.chemsc;

ERRppm = "%2.2f" % "(prLPE.errppm)";

SPECIESUM = "LPE [%d:%d]" % "((prLPE.chemsc)[C] - 5, (prLPE.chemsc)[db] - 0.5)";

INTENS = prLPE.intensity;;

################ end script ##################

QUERYNAME = PhosphaticAcid;

DEFINE prPA = 'C[27..51] H[30..130] O[8] N[1] P[1]' WITH DBR = (1.5,13.5), CHG = +1;

IDENTIFY

# marking

prPA IN MS1+

SUCHTHAT

isOdd(prPA.chemsc[C])

REPORT

MASS = "prPA.mass;

CHEMSC = prPA.chemsc;

ERRppm = "%2.2f" % "(prPA.errppm)";

SPECIESUM = "PA [%d:%d]" % "((prPA.chemsc)[C] - 3, (prPA.chemsc)[db] - 1.5)";

INTENS = prPA.intensity;;

################ end script ##################

QUERYNAME = Phosphatidylcholine;

DEFINE prPC = 'C[32..60] H[30..130] O[8] N[1] P[1] Na[0..1]' WITH DBR = (1.5,13.5), CHG = +1;

IDENTIFY

# marking

prPC IN MS1+

SUCHTHAT

isEven(prPC.chemsc[C])

REPORT

MASS = "prPC.mass;

CHEMSC = prPC.chemsc;

ERRppm = "%2.2f" % "(prPC.errppm)";

SPECIESUM = "PC [%d:%d]" % "((prPC.chemsc)[C] - 8, (prPC.chemsc)[db] - 1.5)";

INTENS = prPC.intensity;;

################ end script ##################

QUERYNAME = PCIS;

DEFINE prPCOO= 'C[48] H[100..101] O[6] N[1] P[1] Na[0..1]' WITH DBR = (-0.5,0), CHG = +1;

IDENTIFY

# marking

prPCOO IN MS1+

SUCHTHAT

isEven(prPCOO.chemsc[C])

REPORT

MASS = "prPCOO.mass;

CHEMSC = prPCOO.chemsc;

ERRppm = "%2.2f" % "(prPCOO.errppm)";

SPECIESUM = "PC-IS";

INTENS = prPCOO.intensity;;

################ end script ##################

QUERYNAME = Phosphatidylcholineether;

DEFINE prPCO = 'C[32..60] H[30..130] O[7] N[1] P[1] Na[0..1]' WITH DBR = (0.5,12.5), CHG = +1;

IDENTIFY

# marking

prPCO IN MS1+

SUCHTHAT

isEven(prPCO.chemsc[C])

REPORT

MASS = "prPCO.mass;

CHEMSC = prPCO.chemsc;

ERRppm = "%2.2f" % "(prPCO.errppm)";

SPECIESUM = "PC-O [%d:%d]" % "((prPCO.chemsc)[C] - 8, (prPCO.chemsc)[db] - 0.5)";

INTENS = prPCO.intensity;;

################ end script ##################

QUERYNAME = Phosphatidylethanolamine;

DEFINE prPE = 'C[29..53] H[30..130] O[8] N[1] P[1] Na[0..1]' WITH DBR = (1.5,13.5), CHG = +1;

IDENTIFY

# marking

prPE IN MS1+

SUCHTHAT

isOdd(prPE.chemsc[C])

REPORT

MASS = "prPE.mass;

CHEMSC = prPE.chemsc;

ERRppm = "%2.2f" % "(prPE.errppm)";

SPECIESUM = "PE [%d:%d]" % "((prPE.chemsc)[C] - 5, (prPE.chemsc)[db] - 1.5)";

INTENS = prPE.intensity;;

################ end script ##################

QUERYNAME = PEIS;

DEFINE prPEOO= 'C[45] H[94..95] O[6] N[1] P[1] Na[0..1]' WITH DBR = (-0.5,0), CHG = +1;

IDENTIFY

# marking

prPEOO IN MS1+

SUCHTHAT

isOdd(prPEOO.chemsc[C])

REPORT

MASS = "prPEOO.mass;

CHEMSC = prPEOO.chemsc;

ERRppm = "%2.2f" % "(prPEOO.errppm)";

SPECIESUM = "PE-IS";

INTENS = prPEOO.intensity;;

################ end script ##################

QUERYNAME = Phosphatidylethanolamineether;

DEFINE prPEO = 'C[29..53] H[30..130] O[7] N[1] P[1]' WITH DBR = (0.5,12.5), CHG = +1;

IDENTIFY

# marking

prPEO IN MS1+

SUCHTHAT

isOdd(prPEO.chemsc[C])

REPORT

MASS = "prPEO.mass;

CHEMSC = prPEO.chemsc;

ERRppm = "%2.2f" % "(prPEO.errppm)";

SPECIESUM = "PE-O [%d:%d]" % "((prPEO.chemsc)[C] - 5, (prPEO.chemsc)[db] - 0.5)";

INTENS = prPEO.intensity;;

################ end script ##################

QUERYNAME = Phosphatidylglycerole;

DEFINE prPG = 'C[30..54] H[30..130] O[10] N[1] P[1]' WITH DBR = (1.5,13.5), CHG = +1;

IDENTIFY

# marking

prPG IN MS1+

SUCHTHAT

isEven(prPG.chemsc[C])

REPORT

MASS = "prPG.mass;

CHEMSC = prPG.chemsc;

ERRppm = "%2.2f" % "(prPG.errppm)";

SPECIESUM = "PG [%d:%d]" % "((prPG.chemsc)[C] - 6, (prPG.chemsc)[db] - 1.5)";

INTENS = prPG.intensity;;

################ end script ##################

QUERYNAME = Phosphatidylinositol;

DEFINE prPI = 'C[33..57] H[30..150] O[13] N[1] P[1]' WITH DBR = (2.5,14.5), CHG = +1;

IDENTIFY

# marking

prPI IN MS1+

SUCHTHAT

isOdd(prPI.chemsc[C])

REPORT

MASS = "prPI.mass;

CHEMSC = prPI.chemsc;

ERRppm = "%2.2f" % "(prPI.errppm)";

SPECIESUM = "PI [%d:%d]" % "((prPI.chemsc)[C] - 9, (prPI.chemsc)[db] - 2.5)";

INTENS = prPI.intensity;;

################ end script ##################

QUERYNAME = Phosphatidylserine;

DEFINE prPS = 'C[30..54] H[30..130] O[10] N[1] P[1] Na[0..1]' WITH DBR = (2.5,14.5), CHG = +1;

IDENTIFY

# marking

prPS IN MS1+

SUCHTHAT

isEven(prPS.chemsc[C])

REPORT

MASS = "prPS.mass;

CHEMSC = prPS.chemsc;

ERRppm = "%2.2f" % "(prPS.errppm)";

SPECIESUM = "PS [%d:%d]" % "((prPS.chemsc)[C] - 6, (prPS.chemsc)[db] - 2.5)";

INTENS = prPS.intensity;;

################ end script ##################

QUERYNAME = Sphingomyelin;

DEFINE prSM = 'C[35..47] H[30..130] O[6..7] N[2] P[1] Na[0..1]' WITH DBR = (0.5,7.5), CHG = +1;

IDENTIFY

# marking

prSM IN MS1+

REPORT

MASS = "prSM.mass;

CHEMSC = prSM.chemsc;

ERRppm = "%2.2f" % "(prSM.errppm)";

SPECIESUM = "SM [%d:%d;%d]" % "((prSM.chemsc)[C] - 5, (prSM.chemsc)[db] - 0.5, (prSM.chemsc)[O] - 6)";

INTENS = prSM.intensity;;

################ end script ##################

QUERYNAME = Triacylglycererols;

DEFINE prTAG= 'C[39..75] H[30..150] O[6] N[1]' WITH DBR = (1.5,19.5), CHG = +1;

IDENTIFY

# marking

prTAG IN MS1+

REPORT

MASS = "prTAG.mass;

CHEMSC = prTAG.chemsc;

ERRppm = "%2.2f" % "(prTAG.errppm)";

SPECIESUM = "TAG [%d:%d]" % "((prTAG.chemsc)[C] - 3, (prTAG.chemsc)[db] - 1.5)";

INTENS = prTAG.intensity;;

################ end script ##################
